# Structural basis for continued antibody evasion by the SARS-CoV-2 receptor binding domain

**DOI:** 10.1126/science.abl6251

**Published:** 2021-12-02

**Authors:** Katherine G. Nabel, Sarah A. Clark, Sundaresh Shankar, Junhua Pan, Lars E. Clark, Pan Yang, Adrian Coscia, Lindsay G. A. McKay, Haley H. Varnum, Vesna Brusic, Nicole V. Tolan, Guohai Zhou, Michaël Desjardins, Sarah E. Turbett, Sanjat Kanjilal, Amy C. Sherman, Anand Dighe, Regina C. LaRocque, Edward T. Ryan, Casey Tylek, Joel F. Cohen-Solal, Anhdao T. Darcy, Davide Tavella, Anca Clabbers, Yao Fan, Anthony Griffiths, Ivan R. Correia, Jane Seagal, Lindsey R. Baden, Richelle C. Charles, Jonathan Abraham

**Affiliations:** 1 Department of Microbiology, Blavatnik Institute, Harvard Medical School, Boston, MA 02115, USA.; 2 Department of Microbiology and National Emerging Infectious Diseases Laboratories, Boston University School of Medicine, Boston, MA 02118, USA.; 3 Department of Pathology, Brigham and Women’s Hospital, Boston, MA 02115, USA.; 4 Center for Clinical Investigation, Brigham and Women’s Hospital, Boston, MA 02115, USA.; 5 Division of Infectious Diseases, Department of Medicine, Brigham and Women’s Hospital, Boston, MA 02115, USA.; 6 Division of Infectious Diseases, Department of Medicine, Centre Hospitalier de l’Université de Montréal, Montreal QC H2X 0C1, Canada.; 7 Division of Infectious Diseases, Department of Medicine, Massachusetts General Hospital, Boston, MA 02114, USA.; 8 Department of Pathology, Massachusetts General Hospital, Boston, MA 02114, USA.; 9 Department of Population Medicine, Harvard Pilgrim Health Care Institute and Harvard Medical School, Boston, MA 02215, USA.; 10 Department of Immunology and Infectious Diseases, Harvard T.H. Chan School of Public Health, Boston, MA 02215, USA.; 11 AbbVie Bioresearch Center, Worcester, MA 01605, USA.; 12 Massachusetts Consortium on Pathogen Readiness, Boston, MA, USA.; 13 Broad Institute of Harvard and MIT, Cambridge, MA 02142, USA.

## Abstract

Many studies have examined the impact of severe acute respiratory syndrome coronavirus 2 (SARS-CoV-2) variants on neutralizing antibody activity after they have become dominant strains. Here, we evaluate the consequences of further viral evolution. We demonstrate mechanisms through which the SARS-CoV-2 receptor binding domain (RBD) can tolerate large numbers of simultaneous antibody escape mutations and show that pseudotypes containing up to seven mutations, as opposed to the one to three found in previously studied variants of concern, are more resistant to neutralization by therapeutic antibodies and serum from vaccine recipients. We identify an antibody that binds the RBD core to neutralize pseudotypes for all tested variants but show that the RBD can acquire an N-linked glycan to escape neutralization. Our findings portend continued emergence of escape variants as SARS-CoV-2 adapts to humans.

## Evasive maneuvers by the spike protein

Throughout the course of the COVID-19 pandemic, variants have arisen in the severe acute respiratory syndrome coronavirus 2 (SARS-CoV-2) virus that increase infectivity or reduce its susceptibility to existing antibodies. Nabel *et al*. focus on mutations in the spike protein, which is found on the viral surface and is responsible for binding and entering host cells, and show that the structure exhibits plasticity in resisting neutralizing antibodies. Working with pseudoviruses, the authors identify combinatorial mutations that might lead to resistance. They identify an antibody that neutralizes a pseudotype with a highly mutated spike, but also show that SARS-CoV-2 can acquire a glycan to escape this neutralization. Understanding the consequences of changes in the antigenic landscape of the spike protein is important if we are to rapidly respond to new variants of concern. —VV

## Abstract (structured)

**INTRODUCTION:**

As severe acute respiratory syndrome coronavirus 2 (SARS-CoV-2) replicates under selective pressure from natural and vaccine-induced immunity, variants of concern (VOCs) continue to emerge. Through adaptative evolution, these variants acquire mutations in the spike protein receptor binding domain (RBD) that binds the cellular receptor angiotensin-converting enzyme 2 (ACE2). The effects of spike protein mutations on immune responses make it important to monitor viral variants. While previously studied VOCs contain one to three RBD mutations that at times overlap, the potential for composite variants that contain larger numbers of mutations is being closely monitored.

**RATIONALE:**

As parts of the world continue to face waves of infection and mitigation strategies are relaxed, viral replication in human hosts under antibody selective pressure continues to shape the antigenic landscape of the SARS-CoV-2 spike protein. As variants containing composite mutations begin to emerge, proactive approaches examining the impact of variants before they become dominant strains are critical.

**RESULTS:**

We determined the x-ray crystal structure of human ACE2 in complex with a SARS-CoV-2 RBD that contains six substitutions that arose during persistent infection of an immunocompromised individual. We found that structural plasticity at the RBD–ACE2 interface allowed the RBD to tolerate a large number of mutations while retaining ACE2 affinity. We generated a panel of pseudotypes bearing composite RBD mutations (up to seven) from immunocompromised host-derived sequences and VOCs. Composite variants more adeptly evaded therapeutic antibody neutralization than did previously studied VOCs. After first immunization but before the second dose of an mRNA vaccine, we observed a loss in vaccine recipient serum neutralizing activity for all variants tested, although the severity differed depending on the variant. However, sampling after the second immunization revealed detectable neutralizing activity against all variants in the serum of vaccine recipients, including against a pseudotype that contains seven composite RBD mutations [denoted receptor binding mutant-2 (RBM-2)]. To identify evolutionary barriers that restrict neutralization breadth, we used the SARS-CoV spike protein to isolate a neutralizing antibody from a COVID-19 convalescent donor. Through structural analysis and functional assays, we show that N-linked glycan acquisition by the SARS-CoV-2 RBD confers pseudotype resistance to neutralization by the isolated cross-reactive antibody and at least one other antibody that binds a similar, otherwise highly conserved epitope. Therefore, acquisition of an N-linked glycan on the SARS-CoV-2 RBD is an additional means through which the virus could continue to evade immune responses.

**CONCLUSION:**

We find that accumulation of large numbers of RBD mutations is facilitated by structural plasticity at the RBD–ACE2 interface and further erodes the activity of therapeutic antibodies and serum from vaccine recipients. Furthermore, acquisition of an N-linked glycan on the SARS-CoV-2 RBD is an additional neutralization escape pathway that should be closely monitored during viral antigenic drift.

## Teaser

As it evades antibodies, the SARS-CoV-2 spike protein receptor-binding domain can acquire multiple mutations and a glycan.

## Graphical abstract

**Figure:**
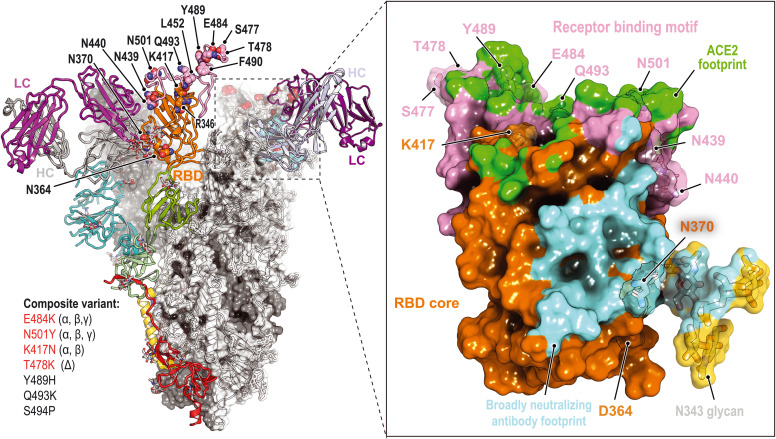
Immune escape at the SARS-CoV-2 spike protein RBD. Structural plasticity accommodates the accumulation of composite substitutions in the RBD ACE2 binding site and allows the RBD to adeptly escape therapeutic antibodies. Cross-neutralizing antibodies bind the RBD core, but acquisition of an N-linked glycan at RBD residue Asn^370^ (N370) drives further neutralization escape. Single-letter abbreviations for the amino acid residues are as follows: D, Asp; E, Glu; F, Phe; H, His; K, Lys; L, Leu; N, Asn; P, Pro; Q, Gln; R, Arg; S, Ser; T, Thr; and Y, Tyr. LC, light chain; HC, heavy chain.

As severe acute respiratory syndrome coronavirus 2 (SARS-CoV-2) continues to replicate in humans under selective pressure from natural and vaccine-induced immunity, variants of concern (VOCs) with increased transmissibility or virulence continue to emerge (*1*). Through adaptive evolution, these variants acquire mutations in the spike (S) protein receptor binding domain (RBD) that binds the cellular receptor human angiotensin-converting enzyme 2 (ACE2) (*1*–*3*). Many of these mutations are within the RBD receptor binding motif (RBM), a hypervariable loop that mediates most of the ACE2 contacts (*2*, *3*). The RBD is the primary target of neutralizing antibodies in naturally acquired or vaccine-elicited humoral immunity (*4*, *5*). The spike protein N-terminal domain (NTD) is also a target of neutralizing antibodies, and VOCs have NTD mutations that include deletions at an antigenic supersite for neutralizing antibody binding (*6*, *7*). The effects of spike protein mutations on immune responses (*8*–*13*) make it important to monitor viral variants.

While previously studied VOCs contain one to three RBD mutations that at times overlap (*1*), the potential for composite variants is being closely monitored. For example, the B.1.617.2 (Delta) variant can acquire the K417N_RBD_ mutation (Lys^417^→Asn) found in the B.1.351 (Beta) variant, generating the Delta AY.2 variant, for a total of three RBD mutations (Fig. 1A). Similarly, as shown in recently deposited sequences from samples collected in Angola, the Beta variant can acquire the L452R_RBD_ mutation found in the Delta and B.1.429/427 (Epsilon) variants, for a total of four RBD mutations (Fig. 1A and table S1). Further complicating matters, variant monitoring efforts are still undersampling viral evolution. For example, a virus recently sequenced from travelers returning from Tanzania contained a previously undocumented combination of RBD mutations (E484K_RBD_, T478R_RBD_, and R346K_RBD_) with NTD deletions that would likely alter the spike protein antigenic surface and result in antibody escape (table S1).

**Fig. 1.**
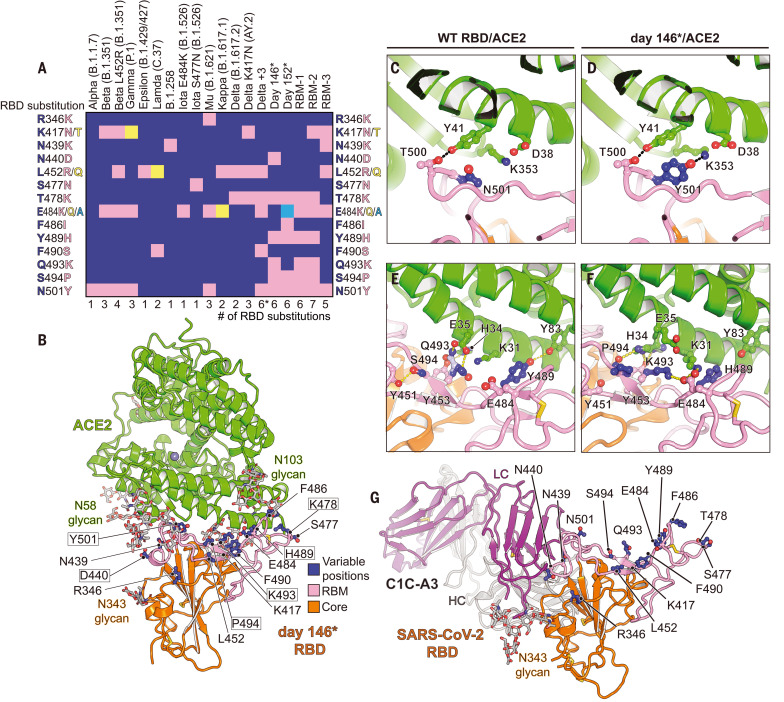
Structure of intrahost evolved RBD bound to human ACE2. (**A**) Key RBD substitutions discussed in the text and the SARS-CoV-2 variants that contain them. Single-letter abbreviations for the amino acid residues are as follows: A, Ala; C, Cys; D, Asp; E, Glu; F, Phe; G, Gly; H, His; I, Ile; K, Lys; L, Leu; M, Met; N, Asn; P, Pro; Q, Gln; R, Arg; S, Ser; T, Thr; V, Val; W, Trp; and Y, Tyr. (**B**) Day 146* RBD–ACE2 ectodomain x-ray crystal structure. RBD residues that are mutated in variants discussed in the text are shown. Boxed residues are mutated in the day 146* RBD as compared with the Wuhan-Hu-1 (wild-type) SARS-CoV-2 RBD. The Delta +3 variant contains an additional RBD mutation that is not shown in the schematic diagram (see table S2). (**C**) Wild-type RBD–ACE2 contacts near N501_RBD_ [Protein Data Bank (PDB) ID 6M0J] (*2*). (**D**) Day 146* RBD contacts near Y501_RBD_. (**E**) Wild-type SARS-CoV-2 RBD–ACE2 interactions near Q493_RBD_. (**F**) Day 146* RBD interactions near K493_RBD_. (**G**) Cryo-EM structure of the SARS-CoV-2 RBD bound to the C1C-A3 antibody Fab. RBD residues discussed in the text are labeled. LC, light chain; HC, heavy chain.

Here, we investigate the structural plasticity of the SARS-CoV-2 spike protein RBD and its capacity to evade neutralizing antibodies.

## Results

### Structure of an evolved receptor binding domain–ACE2 complex

We previously generated two SARS-CoV-2 spike proteins that each contain six RBD changes that were detected during persistent infection of an immunocompromised individual infected with a SARS-CoV-2 strain containing the D614G_S_ mutation (*14*–*16*). This individual received treatment with REGN-COV2 (*17*, *18*), but several of the RBD substitutions had occurred even before administration of this therapeutic antibody cocktail (*14*–*16*). Lentivirus pseudotypes bearing these spike proteins, denoted day 146* and day 152* (Fig. 1A and table S2), were refractory to neutralization by V_H_3-53 heavy chain gene–derived neutralizing antibodies, a potent class of neutralizing antibodies that have been repeatedly isolated from convalescent donors (*19*–*25*). These pseudotypes were also resistant to neutralization by components of REGN-COV2 (*17*, *18*) and by polyclonal immunoglobulin G (IgG) purified from the serum of COVID-19 convalescent donors (*14*). Substitutions in the day 146* and day 152* spike proteins, noted in samples sequenced from this individual in the spring and summer of 2020, foreshadowed those in currently circulating VOCs at three positions: N501_RBD_, E484_RBD_, and T478_RBD_ (Fig. 1A). The day 146* and day 152* spike proteins also contain substitutions that are not in current dominant strains but could have serious effects if acquired. For example, the S494P_RBD_ substitution is a therapeutic antibody (LY-CoV555) escape mutation (*26*) that, as of 27 September 2021, was present in more than 12,000 human-derived SARS-CoV-2 sequences on public research databases (GISAID) (*27*). Additionally, the Q493K_RBD_ mutation, which is found in more than 100 human-derived SARS-CoV-2 sequences on GISAID as of 27 September 2021, confers resistance to multiple therapeutic antibodies [REGN10933, CB6 (LY-CoV016), and LY-CoV555] and V_H_3-53 gene–derived antibodies (*14*, *16*, *17*, *28*).

To determine the impact of their combined mutations on human ACE2 binding, we generated recombinant RBDs for the day 146* and day 152* spike protein mutants. The affinity of the day 152* mutant monomeric RBD for the monomeric ACE2 ectodomain was substantially lower (binding affinity, *K*_d_, of 2.4 μM) than that of wild-type (Wuhan-Hu-1) RBD (54 nM, consistent with other reports) (*3*, *29*), suggesting that its mutations compromise ACE2 binding (fig. S1 and table S3). For comparison, the affinity we measured of the SARS-CoV RBD for human ACE2 was 0.26 μM, about ninefold higher than the affinity for the day 152* RBD (fig. S1 and table S3). The affinity of the day 152* RBD for ACE2 is comparable to that of the RBDs of some bat coronaviruses that are closely related to SARS-CoV-2 and bind human ACE2 (e.g., RaTG13 virus RBD affinity of 3.9 μM) (*30*). The day 146* RBD, however, had a similar affinity (*K*_d_ of 46 nM) for ACE2 as that of the Wuhan-Hu-1 SARS-CoV-2 RBD (fig. S1 and table S3).

We determined the x-ray crystal structure of the day 146* RBD bound to the human ACE2 ectodomain (Fig. 1B, fig. S2, and table S4). This structure is similar to previously determined structures of ACE2–SARS-CoV-2 RBD complexes (*2*, *3*), except we observed contacts between two N-linked glycans on ACE2 (attached to N53_ACE2_ and N90_ACE2_) and the RBD (fig. S3). Removing the N90_ACE2_ glycan, which interacts with the RBD in both copies of the crystal asymmetric unit (fig. S3), increased Wuhan-Hu-1 SARS-CoV-2 and day 146* RBD affinity for ACE2, although the effect was modest (fig. S1 and table S3). This finding is consistent with prior work implicating the N90_ACE2_ glycan, which is removed in a human polymorphism (T92I_ACE2_), as a barrier to SARS-CoV-2 RBD binding to ACE2 (*31*, *32*).

The N501Y_RBD_ substitution is found in multiple VOCs (Fig. 1A); once it surfaced in the immunocompromised individual, it was retained at later time points (*14*–*16*). As also shown in a cryo–electron microscopy (cryo-EM) structure of the SARS-CoV-2 spike protein containing the N501Y_RBD_ substitution bound to ACE2 (*33*), the side chain of Y501_RBD_ interacts with Y41_ACE2_ and K353_ACE2_ with no notable structural change (Fig. 1, C and D). E484_RBD_ is a critical target of antibodies against SARS-CoV-2 and is mutated in several variants (*12*, *34*, *35*). In structures of Wuhan-Hu-1 SARS-CoV-2 RBD bound to ACE2, E484_RBD_ is near but does not directly contact the receptor (Fig. 1E). In the day 146* RBD–ACE2 complex structure, the K493_RBD_ side chain reaches over the RBD surface to recruit the E484_RBD_ side chain to form a new salt bridge with K31_ACE2_ (Fig. 1F). The nearby Y489H_RBD_ mutation, which removes a polar contact with ACE2, better accommodates repositioning of E484_RBD_ because the histidine is smaller than the tyrosine side chain and would avoid potential steric clashes with E484_RBD_ in this binding mode (Fig. 1, E and F). A second rotamer for residue H34_ACE2_ forms additional RBD contacts to fill a gap created by the reorganization of local interactions (Fig. 1, E and F). This structural plasticity may explain how the RBD tolerates an unexpectedly large number of mutations during intrahost evolution yet retains the ability to bind ACE2 tightly. It is also consistent with the large sequence divergence in the RBD residues that contact ACE2 among SARS-related coronaviruses that share this cellular receptor.

### Neutralization escape of therapeutic antibodies

RBD-targeting antibodies can be categorized into classes on the basis of whether they bind an overlapping footprint with ACE2 and recognize only an open or both an open and a closed RBD on the spike protein trimer (*36*). CB6 (equivalent to LY-CoV016 or etesevimab) is a class 1, V_H_3-66–derived antibody that blocks ACE2 binding and can only bind the RBD when it is open, and LY-CoV555 (bamlanivimab) is a class 2 antibody that blocks ACE2 binding but can bind both open and closed RBDs (*21*, *37*). LY-CoV016 and LY-CoV555 are used together as a cocktail and bind epitopes that partially overlap on the RBM such that both cannot bind simultaneously (*21*, *37*). REGN10933 is a class 1 antibody, and REGN10987 is a class 3 antibody that sterically blocks ACE2 binding but binds the RBM outside the main ACE2 binding site; both are used as a cocktail (REGN-COV2) (*17*, *18*).

**Fig. 2.**
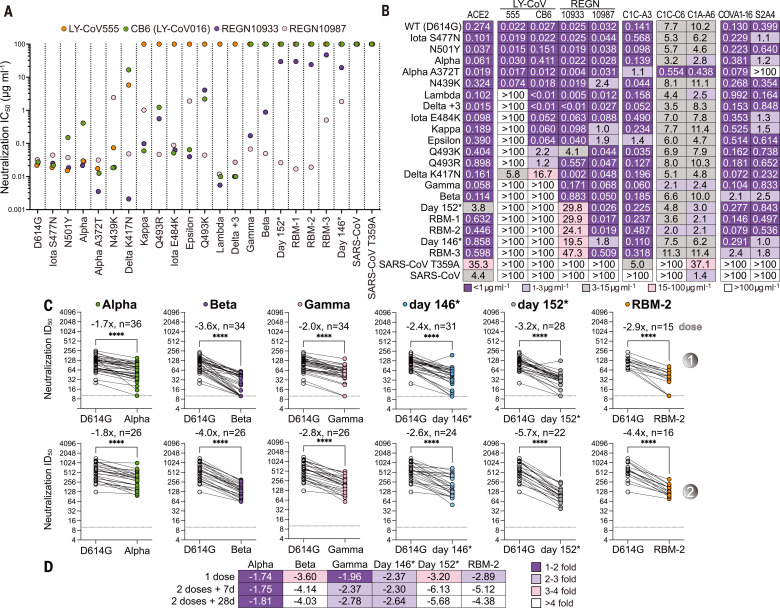
Neutralization escape from therapeutic antibodies and mRNA vaccine–elicited serum. (**A**) Summary of neutralization IC_50_ values for lentivirus pseudotypes with the indicated monoclonal antibodies. (**B**) Tabulated IC_50_ values for lentivirus pseudotypes with the indicated monoclonal antibodies and an ACE2-Fc fusion protein (ACE2). (**C**) Mean ID_50_ neutralization titers for the indicated variant pseudotypes at the time of the second immunization but before vaccination (“dose 1”), or 28 days after second immunization (“dose 2”) with mRNA-1273 or BNT162b2. The fold change of the mean ID_50_ neutralization titer with respect to D614G_S_ pseudotype is shown in each panel. Each experiment was performed twice independently in triplicate (*n* = 6). Wilcoxon matched-pairs signed rank test; ****P < 0.0001. (**D**) Tabulated fold change of mean ID_50_ neutralization titers for the indicated pseudotypes as compared with D614G_S_ pseudotype.

Structural plasticity at the RBD–ACE2 interface suggests that the RBD could tolerate many more mutations than found in current VOCs. We next generated pseudotypes for spike protein variants that contain composite mutations. The Delta variant, which contains the L452R_RBD_ and T478K_RBD_ substitutions, has become a dominant strain across the globe (*38*). We generated a pseudotype for the Delta AY.2 variant, which contains the K417N_RBD_ mutation that is usually found in the Beta variant, and a Delta variant containing the N501Y_RBD_, E484K_RBD_, and F490S_RBD_ mutations usually found in the Beta, P.1 (Gamma), and C.37 (Lambda) variants (referred to here as “Delta +3”) (Fig. 1A and tables S1 and S2). The set of RBD mutations for the latter strain occurred in deposited sequences from samples collected in Turkey (table S1). We also generated pseudotypes in which we combined spike protein substitutions detected in the immunocompromised host with mutations found in the Beta variant, which we chose because this VOC is highly resistant to antibody neutralization (*10*, *12*, *39*). Starting with a day 146* spike protein sequence, which contains an NTD deletion, we incorporated either one (E484K_RBD_) or two (E484K_RBD_ and K417N_RBD_) additional substitutions; these are referred to as receptor binding mutant-1 (RBM-1) and RBM-2, respectively (Fig. 1A and table S2). Additionally, starting with the Beta variant spike protein sequence, we generated a variant pseudotype that contains two additional mutations associated with immune evasion (L452R_RBD_ and N439K_RBD_) (*40*, *41*). This pseudotype is referred to as RBM-3 (Fig. 1A and table S2). An ACE2-Fc fusion protein neutralized RBM-1, RBM-2, and RBM-3 pseudotypes, suggesting that all entered cells by binding ACE2 (Fig. 2B and fig. S4A).

We tested the activity of therapeutic antibodies against Delta AY.2, Delta +3, RBM-1, RBM-2, RBM-3, and additional variant pseudotypes with known resistance profiles to serve as comparators in the same assay (Fig. 2, A and B, and fig. S4A). LY-CoV555 was the most affected by escape mutations, followed by CB6 (from which LY-CoV016 is derived) (Fig. 2, A and B, and fig. S4A). The Q493K_RBD_ mutation conferred absolute resistance to LY-CoV555, generated 80-fold resistance to CB6, and also compromised REGN10933 activity, consistent with previous reports (Fig. 2, A and B, and fig. S4A) (*14*, *16*, *17*, *26*). In addition to the expected loss of activity of LY-CoV555 and CB6 against Beta and Gamma variants (*9*, *11*, *12*, *42*), LY-CoV555 and CB6 lost all activity against day 146*, day 152*, RBM-1, RBM-2, and RBM-3 pseudotypes (Fig. 2, A and B, and fig. S4A). Whereas the Delta variant is known to resist neutralization by LY-CoV555 but retain sensitivity to neutralization by CB6/LY-CoV016 (*38*), the Delta AY.2 pseudotype was resistant to both agents (Fig. 2, A and B, and fig. S4A). This is expected because CB6/LY-CoV016 is derived from a V_H_3-66 antibody (*21*), and the additional mutation the Delta AY.2 variant contains with respect to Delta (K417N_RBD_) confers resistance to CB6/LY-CoV16 and other members of the V_H_3-53 and V_H_3-66 class of neutralizing antibodies (*9*, *14*, *16*, *26*, *43*). The Delta +3 pseudotype, which despite containing six RBD mutations does not contain the K417N_RBD_ substitution, only escaped neutralization by LY-CoV555 (Fig. 2, A and B; fig. S4A; and table S2). Although the distribution of LY-CoV016 and LY-CoV555 was paused in the United States in the summer of 2021 as the prevalence of Gamma and Beta VOCs increased, the distribution of this antibody cocktail has since been resumed with the rise of Delta as the predominant strain. However, our findings emphasize the importance of close monitoring of Delta AY.2 and of other Delta variants for acquisition of the K417N_RBD_ mutation.

Although REGN10933 lost substantial activity against the Beta variant, which is consistent with other reports (*9*, *12*, *42*), it still had a median inhibitory concentration (IC_50_) value of <1 μg ml^−1^ in our assays (Fig. 2, A and B, and fig. S4A). However, resistance markedly worsened with the day 146*, day 152*, RBM-1, RBM-2, and RBM-3 pseudotypes, with 800- to 1900-fold loss of neutralizing activity (IC_50_ values ranging from 20 to 47 μg ml^−1^). REGN10987 potently neutralized many of the variant pseudotypes we examined. While we observed the expected resistance to REGN10987 neutralization by variants containing the N439K_RBD_ or the adjacent N440D_RBD_ substitutions (*14*, *16*), we also observed some loss of activity against Epsilon and B.1.617.1 (Kappa), which was not expected because none of their substitutions fall within the REGN10987 RBD footprint (Fig. 1A and Movie 1). Nonetheless, other reports have also noted varying degrees of modest in vitro resistance of Epsilon and Kappa variants to REGN10987 neutralization (*39*, *42*). Notably, the day 146* and RBM-3 pseudotypes were the only ones to gain resistance to both antibodies in REGN-COV2, because they contain substitutions in the REGN10933 (e.g., Q493K_RBD_, or E484K_RBD_ and K417N_RBD_) and the REGN10987 binding sites (N439K_RBD_ or N440D_RBD_) (Fig. 2, A and B; fig. S4A; and Movie 1) (*14*). We observed on GISAID instances of “day 146*–like” viruses that would be expected to resist neutralization by LY-CoV555, LY-CoV016, REGN10933, and REGN10987, because they contain the Q493K_RBD_ and N439K_RBD_ substitutions. One strain contains the N501Y_RBD_, Q493K_RBD_, and N439K_RBD_ mutations (sequenced once in South Africa), and the other contains the N501Y_RBD_, Q493K_RBD_, L452R_RBD_, N439K_RBD_, and N440F_RBD_ mutations (sequenced once in the United Kingdom) (table S1).

**Movie 1.**
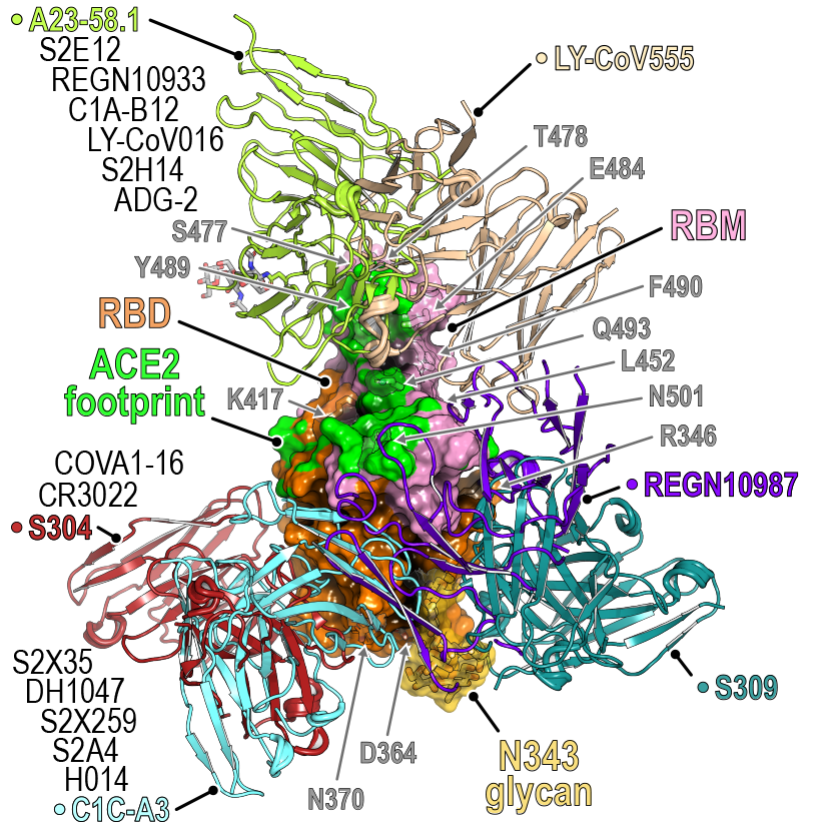
Antibody footprints on an evolving SARS-CoV-2 RBD. Antibodies are classified according to Barnes *et al.* (*36*). PDB IDs are listed in parentheses. Key RBD residues discussed in the main text are highlighted.

The broadly neutralizing antibody S309 (*44*), a class 3 antibody that binds the RBD but does not interfere with ACE2 binding and from which the therapeutic antibody sotrovimab is derived, was active against all variants we tested (fig. S4A). However, we could not calculate reliable neutralization IC_50_ values because of variable non-neutralizable pseudotype fractions (fig. S4A). The presence of a non-neutralizable fraction is unexplained but has been noted in other reports when human cells overexpressing ACE2, as opposed to African green monkey (Vero) target cells, were used to examine S309 neutralizing activity (*45*, *46*).

### Neutralization escape of mRNA vaccine recipient sera

Messenger RNA (mRNA)–based vaccines encoding the SARS-CoV-2 spike protein elicit robust neutralizing antibody responses (*47*–*49*). We directly compared immune evasion of the day 146*, day 152*, and RBM-2 pseudotypes to the B.1.1.7 (Alpha), Beta, and Gamma pseudotypes in sera obtained from individuals who had received a two-dose series of an mRNA vaccine (BNT162b2 or mRNA-1273) (Fig. 2, C and D, and fig. S5). In addition to RBD substitutions, day 146*, day 152*, RBM-1, and RBM-2 spike proteins all contain NTD deletions spanning residues 141 to 144, which are positioned near NTD mutations in Alpha, Beta, and Delta in a key antigenic supersite (table S2) (*6*, *7*). As similar NTD deletions found in Alpha, Beta, and Delta prevent binding of some neutralizing antibodies (*6*, *7*, *46*), they would be expected to escape neutralization by some NTD-targeting antibodies in addition to escaping neutralization by RBD-targeting antibodies. After initial immunization but before the second dose, we observed a loss in neutralizing activity for all variants, although the severity of this loss varied. Variants that contain any substitution at E484_RBD_ combined with an NTD deletion (Beta, day 152*, and RBM-2) were more effective at evading antibody responses than variants that had an E484_RBD_ substitution without an NTD deletion (Gamma) or an NTD deletion but no E484_RBD_ substitution (day 146*) (Fig. 2, C and D; fig. S5; and table S2). These findings are consistent with the role of E484_RBD_ as a major driver in neutralization escape of polyclonal antibody responses to SARS-CoV-2 (*35*) and observations that Beta more robustly escapes antibody neutralization than Gamma (*9*, *13*). They further suggest that variants that have an NTD supersite deletion and an E484_RBD_ substitution are the most concerning when it comes to resistance to polyclonal antibodies.

One-quarter of sampled individuals had no detectable activity against the Beta and RBM-2 pseudotypes after a single immunization (Fig. 2, C and D). However, sampling at 7 and 28 days after the second immunization revealed detectable neutralizing activity against all variants in all vaccine recipients, including against the RBM-2 pseudotype, which contains seven RBD mutations (Fig. 2, C and D, and fig. S5). Thus, repeated administration of an mRNA vaccine encoding constructs of the SARS-CoV-2 spike protein used in current formulations may provide sufficient neutralizing antibody breadth and potency to yield baseline serum neutralizing activity against variants that are more extensively mutated than the current dominant strains.

### Identification of SARS-CoV cross-reactive antibodies

**Fig. 3.**
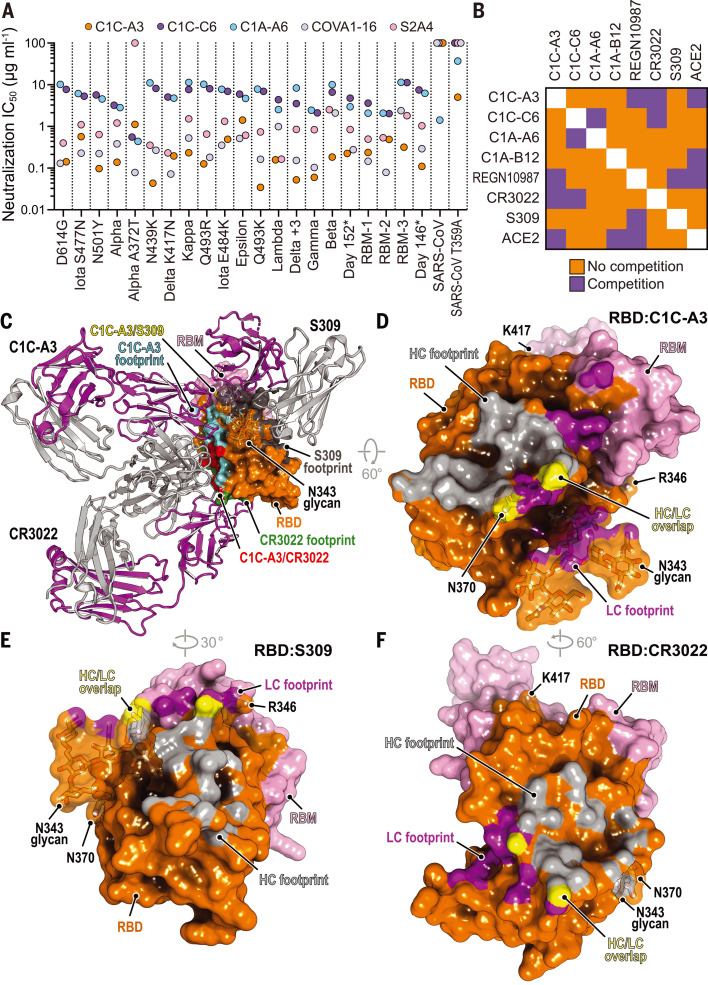
Neutralization of SARS-CoV-2 variants by an RBD core–targeting antibody. (**A**) Summary of neutralization IC_50_ values for pseudotypes and the indicated antibodies. (**B**) Summary of the results of BLI-based competition assays. (**C**) Superposition of the CR3022 (PDB ID 6W41) (*55*) and S309 (PDB ID 6WPS) (*44*) structures onto the C1C-A3–bound RBD structure. Antibody Fabs are shown as ribbon diagrams, and the RBD is shown in surface representation. Antibody footprints are shown on the RBD surface. (**D**) RBD footprint of C1C-A3. (**E**) RBD footprint of S309 (PDB ID 6WPS) (*44*). (**F**) RBD footprint of CR3022 (PDB ID 6W41) (*55*). In panels (D) to (F), key RBD residues discussed in the main text are highlighted.

The RBD is also the major target of neutralizing antibodies against SARS-CoV, which caused a small outbreak of viral pneumonia from 2003 to 2004, although with a much higher case fatality rate (*50*, *51*). Polyclonal antibody responses against SARS-CoV-2 poorly cross-neutralize SARS-CoV (*52*, *53*). To identify barriers that restrict neutralization breadth, we performed single memory B cell sorting with the SARS-CoV spike protein to mine the memory B cell repertoire of a COVID-19 convalescent individual (“C1”). Polyclonal IgG from C1 plasma neutralized SARS-CoV-2 pseudotype but had weak activity against SARS-CoV pseudotype (fig. S6A). From C1 peripheral blood mononuclear cells, using a prefusion stabilized SARS-CoV spike protein (S2P) (*54*), we cloned 17 cross-reactive antibodies. Of these, 11 antibodies bound both the SARS-CoV and the SARS-CoV-2 spike protein in an enzyme-linked immunosorbent assay (ELISA) (fig. S6C and table S5). Only two RBD-binding antibodies, C1C-A3 (“A3”) and C1C-C6 (“C6”), neutralized SARS-CoV-2 pseudotypes in our assays (Figs. 2B and 3A and fig. S6F). Despite binding to the SARS-CoV spike protein and RBD by ELISA, A3 and C6 did not neutralize SARS-CoV pseudotype (fig. S6, F and G). We also included C1A-A6 (“A6”) in these assays, a SARS-CoV-2 neutralizing antibody we previously isolated from the C1 donor using prefusion stabilized SARS-CoV-2 S2P in single B cell sorting experiments (*14*). Unlike A3 and C6, A6 neutralized SARS-CoV pseudotypes (Figs. 2B and 3A and fig. S6F). We determined Fab RBD binding affinities using biolayer interferometry (BLI) (fig. S7 and table S3) and confirmed A3 and A6 activity against infectious SARS-CoV-2 in a plaque reduction neutralization assay (fig. S4B).

A3 neutralized almost all SARS-CoV-2 variant pseudotypes with a neutralization IC_50_ value of <1 μg ml^−1^, including Beta, Gamma, Delta AY.2, Delta +3, RBM-1, RBM-2, and RBM-3 pseudotypes; the Epsilon variant was the only exception, with an IC_50_ value of 1.9 μg ml^−1^ (Figs. 2B and 3A and fig. S4A). C6 and A6 also broadly neutralized variants, but with higher baseline IC_50_ values, even against D614G_S_ pseudotypes (ranging from 2.0 to 11.4 μg ml^−1^) (Figs. 2B and 3A and fig. S4A).

To determine where on the RBD A3, C6, and A6 bind, we carried out competition studies with C1A-B12 (*14*), a class 1 antibody; REGN10987 (*17*, *18*) and S309 (*44*), two class 3 antibodies; and CR3022 (*55*), a class 4 antibody (Fig. 3B, fig. S8, and Movie 1). A3 competed with CR3022 and REGN10987 for RBD binding, C6 competed with CR3022, and C6 and A6 competed with each other (Fig. 3B and fig. S8). A6 did not compete with any of the other antibodies tested. Among A3, C6, and A6, only A3 competed with binding of an ACE2-Fc fusion protein, suggesting that A3 blocks cellular attachment.

### Antibody C1C-A3 binds the conserved RBD core

**Fig. 4.**
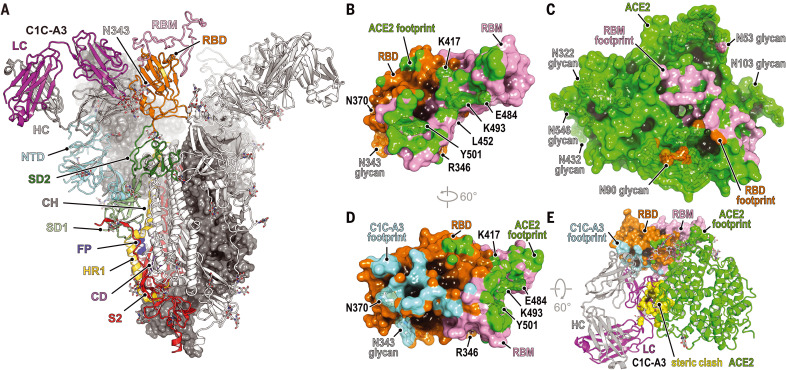
Structural basis for C1C-A3 neutralization. (**A**) Cryo-EM structure of the C1C-A3–Fab SARS-CoV-2 spike protein complex. Two of the three spike protein protomers are shown in surface representation. One protomer is shown as a ribbon diagram with labeled subdomains. The trimer model shown was generated by superposition of an RBD–C1C-A3 Fab model generated by subparticle classification of the RBD region onto the coordinates of the trimeric spike protein–C1C-A3 Fab complex (see materials and methods). SD1, subdomain 1; SD2, subdomain 2; FP, fusion peptide; HR1, heptad repeat 1; CD, connector domain; S2, additional portions of S2 subunit. (**B**) Surface representation of the SARS-CoV-2 day 146* RBD showing the ACE2 footprint, including surfaces contacted by ACE2 N-linked glycans. Key RBD positions discussed in the text are labeled. (**C**) Surface representation of ACE2, showing the day 146* RBD and RBM footprints. (**D**) Surface representation of the RBD highlighting C1C-A3 Fab and ACE2 footprints. (**E**) Overlay of the C1C-A3 Fab–RBD complex with the day 146* RBD–ACE2 complex. Atoms within 1.54 Å of each other are shown in yellow surface representation to highlight steric clashes. Key RBD residues discussed in the text are labeled in (B) and (D).

We determined the 3.1-Å cryo-EM structure of the A3 Fab bound to the SARS-CoV-2 spike protein ectodomain (Fig. 4A, figs. S9 and S10, and table S6). A3 binds the RBD core with the spike protein trapped in the three open RBD conformation (Fig. 4A). In agreement with competition assays (Fig. 3B), A3 interacts with RBD residues that overlap significantly with those of CR3022 (Fig. 3, C, D, and F, and Movie 1). A3 is therefore a class 4 antibody, a class that includes CR3022, S2A4, S304, S2X35, H014, COVA1-16, S2X259, and DH1047 (*4*, *56*–*59*) (Movie 1 and fig. S11). Although the A3 and S309 footprints on the RBD do not overlap, and S309 (a class 3 antibody) can bind the closed spike protein trimer (*44*), both antibodies contact the N-linked glycan attached to N343_RBD_ but approach it from different faces (Fig. 3C and Movie 1).

The A3 Fab avoids the RBD–ACE2 interface, which contains the majority of key antibody escape mutations, but, like other class 4 antibodies, nonetheless binds the RBD in a manner that would sterically interfere with ACE2 binding (Fig. 4, B to E, and fig. S11). On the basis of its epitope, in addition to retaining activity against all variants we tested, A3 would also have activity against emergent and preemergent SARS-CoV-2 variants; these include a variant sequenced from travelers from Tanzania that contains the E484K_RBD_, T478R_RBD_, and R346K_RBD_ mutations, and B.1.621 (Mu), a variant detected early in 2021 in Colombia that has since spread internationally and contains the E484K_RBD_, N501Y_RBD_, and R346K_RBD_ mutations (Figs. 1G and 4D and table S1). The R346K_RBD_ mutation falls within the RBD core and is in the S309 binding site but is not within A3’s footprint (Fig. 3, D and E, and Movie 1). However, S309 would likely retain activity against SARS-CoV-2 variants that contain the R346K_RBD_ mutation, as the residue that is at the position analogous to SARS-CoV R346_RBD_ is a lysine in SARS-CoV, and S309 neutralizes both SARS-CoV and SARS-CoV-2 (*44*, *60*).

### RBD core glycan addition drives neutralization escape

**Fig. 5.**
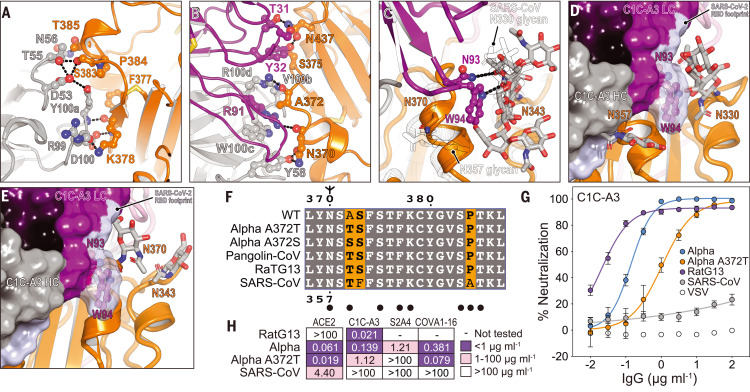
Structural basis for immune evasion of a RBD core–targeting antibody. (**A** and **B**) C1C-A3 antibody contacts with the SARS-CoV-2 RBD core. (**C**) C1C-A3 contacts with the N343_RBD_ glycan with structural superposition of the SARS-CoV RBD (PDB ID 6NB6) (*78*). N-linked glycans found at N330_RBD_ and N357_RBD_ in SARS-CoV and the analogous N343_RBD_ and N370_RBD_ positions in SARS-CoV-2 are highlighted. (**D**) Superposition of the C1C-A3 Fab–SARS-CoV-2 RBD structure with the SARS-CoV RBD (PDB ID 6NB6) (*78*) showing that a glycan attached at SARS-CoV N357_RBD_ may interfere with antibody binding. The SARS-CoV-2 RBD is not shown for clarity. (**E**) Superposition of the C1C-A3–SARS-CoV-2 RBD with the RaTG13 virus RBD (PDB ID 7CN4) (*79*) showing that a glycan attached at RaTG13 virus N370_RBD_ would be more readily accommodated because the helix that contains it would be rotated away from the Fab. The SARS-CoV-2 RBD is omitted for clarity. (**F**) Sequence alignment of the RBD core region contacted by C1C-A3. SARS-CoV-2 numbering is shown at the top of the alignment, and SARS-CoV numbering is shown at the bottom. Circles indicate antibody contacts. (**G**) C1C-A3 neutralization curves for the indicated lentivirus pseudotypes. Data are plotted as the mean ± standard deviation of the mean. The experiment was performed twice in triplicate (*n* = 6). For some data points, error bars are smaller than symbols. (**H**) Tabulated neutralization IC_50_ values for the indicated pseudotypes.

Despite A3’s breadth against SARS-CoV-2 variant pseudotypes (Figs. 2B and 3A), A3 does not neutralize SARS-CoV pseudotype (fig. S6, F and G). The A3 epitope is highly conserved between SARS-CoV-2 and SARS-CoV; however, N370_RBD_ is a site of N-linked glycosylation in SARS-CoV (N357_RBD_ in SARS-CoV numbering) and in animal coronaviruses but not in SARS-CoV-2 (Fig. 5, A to C and F) (*61*). An N-linked glycan attached to SARS-CoV-2 N370_RBD_ would introduce steric clashes with the A3 antibody heavy and light chains (Fig. 5D). Furthermore, calculations of antibody-accessible surface areas using molecular dynamic trajectories of a fully glycosylated SARS-CoV-2 spike protein with a modeled N370_RBD_ glycan suggest that its addition would restrict A3 epitope access and could also affect binding of other class 4 antibodies (fig. S12) (*61*, *62*).

Partial occupancy of the glycan attached to SARS-CoV N357_RBD_ in recombinant protein preparations may explain why we observed spike protein and RBD binding but a lack of SARS-CoV pseudotype neutralization (fig. S6, F and G). In surface plasmon resonance binding assays, A3 IgG bound tightly to the SARS-CoV RBD only when the RBD was enzymatically deglycosylated (fig. S13 and table S7). Consistent with the role of the SARS-CoV N357_RBD_ N-linked glycan as a barrier to A3 neutralization, introducing a substitution that would prevent its addition (T359A_RBD_) sensitized SARS-CoV pseudotypes to A3 neutralization (IC_50_ value of 5 μg ml^−1^) (Fig. 2B and fig. S4A).

The A372S/T_RBD_ mutations, which would introduce an N-linked glycosylation motif and allow for modification of N370_RBD_ in the SARS-CoV-2 spike protein, are found in human-derived SARS-CoV-2 sequences (GISAID) (*27*), including on sequences for VOCs Alpha and Delta, without apparent geographic restriction (48 sequence counts as of 10 October 2021 and detected in at least 14 countries) (Fig. 5F and table S8). Although the mutations are currently rare, their presence in sequence databases suggests that SARS-CoV-2 strains containing these mutations can replicate in humans. To confirm that an N-linked glycan could be added to N370_RBD_, we conducted glycan analysis on recombinant SARS-CoV-2 RBD containing the A372S_RBD_ substitution and observed 90% occupancy of an N-linked glycan at position N370_RBD_ (fig. S13B).

Because acquisition of a putative N-linked glycan at N370_RBD_ was the most frequent on the Alpha variant at the time of our initial analysis, we generated an Alpha pseudotype that contains the A372T_RBD_ substitution (Alpha A372T). We tested the effect of this substitution on three class 4 antibodies: A3, the antibody we isolated here; S2A4, an antibody that does not cross-react with the SARS-CoV RBD (*4*); and COVA1-16, an antibody that has weak cross-neutralizing activity against SARS-CoV (*57*). The mutation resulted in eightfold greater resistance to A3 neutralization (IC_50_ value of 1.1 μg ml^−1^, as compared with 0.14 μg ml^−1^ with the Alpha pseudotype) and complete resistance to S2A4 neutralization (Figs. 2B, 3A, and 5, G and H, and fig. S4A). S2A4 and COVA1-16 neutralized variants with potency that was overall comparable to A3 in most cases (Figs. 2B and 3A and fig. S4A). COVA1-16, probably because it has some activity against SARS-CoV [above the limit of detection in our assays, but 29 μg ml^−1^ as reported by Liu *et al*. (*57*)], retained activity against Alpha A372T pseudotype (Figs. 2B, 3A, and 5H, and fig. S4A). The Fab binding pose of certain class 4 antibodies, therefore, may allow them to avoid steric hindrance from an N-linked glycan attached to N370_RBD_ (S2X259 is one such antibody) (Movie 1) (*56*).

### Antibody C1C-A3 neutralizes a related coronavirus

Coronaviruses that circulate in animals and have spike protein RBDs that can bind human ACE2 are a continued threat. RaTG13 virus, which is closely related to SARS-CoV-2 phylogenetically, is one example (*63*). The RaTG13 virus spike protein contains a threonine at RBD position 372, which would allow for N370_RBD_ glycosylation (Fig. 5F). Despite the presence of the N-linked glycan, A3 potently neutralized RaTG13 virus pseudotype (neutralization IC_50_ value of 21 ng ml^−1^), suggesting that A3 neutralization breadth extends to preemergent coronaviruses that are closely related to SARS-CoV-2 (Fig. 5, G and H). Structural superposition reveals that the N370_RBD_ glycan on the RaTG13 RBD is positioned in a manner that may not block A3 epitope access but could interfere with binding of other antibodies that bind nearby epitopes on the RBD core (Fig. 5E).

## Discussion

As variants containing composite mutations begin to emerge, continued SARS-CoV-2 immune evasion will remain a big concern. We found that accumulation of large numbers of RBD mutations, which mimics accelerated spike protein evolution occurring in a persistently infected immunocompromised host (*14*–*16*), is facilitated by structural plasticity at the ACE2–RBD interface (Fig. 1, B to F). The severity of the phenotypes we observed in vitro suggests that further evolved variants will more adeptly escape therapeutic antibody neutralization than currently circulating VOCs, with potential resistance to two-component antibody cocktails (Fig. 2, A and B).

After two mRNA vaccine immunizations and as early as 7 days after the second dose, all mRNA vaccine recipients had detectable neutralizing activity against pseudotypes containing an NTD supersite deletion and RBDs with six to seven mutations (e.g., day 146*, day 152*, and RBM-2), with mean neutralization ID_50_ values decreased by 2.3- to 6.1-fold (Fig. 2, C and D, and fig. S5). While the precise epitopes targeted by this residual vaccine-elicited serum neutralizing activity remain to be determined, we surmise that antibodies targeting the RBD core (e.g., those that bind away from the RBM) at least in part account for some of this activity. As the RBD is a major target of vaccine-elicited and naturally acquired humoral immunity to SARS-CoV-2 (*4*, *5*), and the RBM is a critical site of potent neutralizing antibody binding (*19*, *21*–*25*, *64*) that is the most antibody-accessible and the least masked by glycan and conformational shielding (fig. S12), continued RBM evolution may guide antibody responses toward more conserved neutralizing epitopes on the RBD core.

We mined genome sequences in the GISAID database for substitutions that would introduce additional N-linked glycans onto the RBD. This analysis identified D364N_RBD_ as an additional mutation that would introduce a putative N-linked glycosylation site in a surface-exposed loop in the footprint of some class 4 antibodies (Movie 1). The independent acquisition of N-linked glycosylation sites (through the A372S/T_RBD_ and D364N_RBD_ substitutions) on the same surface of the RBD core, but not on other RBD sites, suggests that this region may be a target of immune selective pressure.

While glycan addition may allow neutralization escape, this change could come at a cost to viral fitness and infectivity. Indeed, the A1114G:T372A mutation that removed the glycan in the SARS-CoV-2 RBD appeared under selective pressure, and addition of the glycan decreases viral replication in human lung epithelial (Calu-3) cells by more than 60-fold (*65*). A recent molecular dynamics study suggests that introducing the glycan at N370_RBD_ in SARS-CoV-2 would favor the closed conformation with the N370_RBD_ glycan stabilizing the closed RBD structure on the trimeric spike protein (*66*). A lack of a glycan at position N370_RBD_, therefore, may increase SARS-CoV-2 ACE2-binding and infectivity by favoring the open state but may also make SARS-CoV-2 more vulnerable to neutralizing antibodies that can only bind the RBD in the open conformation.

Although addition of the N370_RBD_ glycan may be associated with a cost to viral fitness, should the selective immune pressure be considerable at this site over a long enough time scale, this may also afford the virus an opportunity to acquire permissive secondary mutations during evolution that restore viral fitness, as is observed in influenza virus drug resistance (*67*). Such compensatory mutations would be ones that promote ACE2 binding and RBD opening; for example, the D614G_S_ mutation (*68*), which favors the open conformation, and the N501Y_RBD_ mutation, which introduces more favorable interactions with ACE2 (Fig. 1D).

As parts of the world continue to face waves of infection and mitigation strategies are relaxed, viral replication in human hosts under antibody selective pressure will continue to shape the antigenic landscape of the SARS-CoV-2 spike protein. With vigorous variant monitoring efforts underway to help design next-generation antibody-based therapeutics, and with mRNA- or DNA-based vaccines that can be updated to rapidly adapt to new variants, proactively examining the consequences of further viral evolution before the next highly antibody resistant strain emerges is of utmost importance.

## Materials and methods summary

We isolated monoclonal antibodies from the blood of a COVID-19 convalescent individual using single B cell sorting with prefusion-stabilized SARS-CoV spike protein ectodomain as bait and using established protocols (*14*, *54*). We obtained venous blood samples from healthy mRNA-1273 and BNT162b2 vaccine recipients. We produced recombinant glycoproteins and antibodies or Fabs in transiently transfected mammalian cells grown in suspension culture and purified these proteins using affinity-based methods. We used ELISAs to measure antibody binding and BLI or surface plasmon resonance to determine kinetic parameters of binding. We packaged lentivirus pseudotypes by transient transfection of HEK293T cells, as previously described (*14*). We used HEK293T cells expressing human ACE2 in pseudotype neutralization assays or Vero E6 cells in plaque reduction neutralization tests, as previously described (*14*). We collected x-ray diffraction data on crystals of a day 146*–SARS-CoV-2 RBD complex at the Advanced Photon Source (APS, Argonne, IL) NE-CAT beamline and used established procedures for data processing, molecular replacement, atomic model building, and refinement (*69*–*73*). We used mass spectrometry to perform glycopeptide analysis. After data collection on a Titan Krios cryo–electron microscope equipped with a Gatan K3 camera, we used single-particle cryo-EM to determine the structure of a prefusion-stabilized SARS-CoV-2 spike protein ectodomain (*7*) complexed with C1C-A3 Fab complex using established procedures for image processing, atomic model building, and refinement (*72*–*77*).

## Supplementary Material

Figures and Tables

Checklist

Video

## Supplementary Materials

### This PDF file includes:

Materials and Methods Figs. S1 to S13 Tables S1 to S9 References (*80*–*100*)

### Other Supplementary Material for this manuscript includes the following:

MDAR Reproducibility Checklist

## Notes

View/request a protocol for this paper from *Bio-protocol*.
