# Supplementary material for: Structural basis for continued antibody evasion by the SARS-CoV-2 receptor binding domain
Source: Science. 2021 Dec 2;375(6578):eabl6251. doi: 10.1126/science.abl6251 (PMC9127715; doi:10.1126/science.abl6251)
Supplement: Figures and Tables [file science.abl6251_sm.pdf]

Science AAAS

Supplementary Materials for

Structural basis for continued antibody evasion by the SARS-CoV-2 receptor binding domain

Katherine G. Nabel *et al.*

Corresponding author: Jonathan Abraham, jonathan_abraham@hms.harvard.edu

Science **375**, eabl6251 (2022)
DOI: 10.1126/science.abl6251

The PDF file includes:

Materials and Methods
Figs. S1 to S13
Tables S1 to S9
References

Other Supplementary Material for this manuscript includes the following:

MDAR Reproducibility Checklist

Materials and Methods

Donors

Isolation of human monoclonal antibodies from the blood of a COVID-19 convalescent donor was approved by the Harvard Medical School Office of Human Research Administration Institutional Review Board (IRB20-0365) as was the usage of healthy donor control blood (IRB19-0786). We received informed, written consent from a healthy adult male (“C1”) who recovered from confirmed SARS-CoV-2 infection and had mild illness not requiring hospitalization more than five weeks prior to blood donation. We isolated plasma and peripheral blood mononuclear cells (PBMCs) from C1 and a control donor by Ficoll-Paque (GE Healthcare Cat# 17144002) density centrifugation. For studies involving vaccine recipient serum samples, the study was approved by the Institutional Review Boards of Massachusetts General Hospital (2014P002602), Brigham and Women’s Hospital (2006P001197), and Harvard Medical School (IRB21-0367), and written informed consent was obtained from all participants. Participants were healthy adults ($\geq$18 years old) who had received two doses of the SARS-CoV-2 mRNA vaccine, BNT162b2 or mRNA-1273, given 21 days or 28 days apart, respectively. Venous blood samples were collected prior to immunization (day 0), at the time of the second dose but prior to its administration (“dose 1”), and seven days and 28 days after the second vaccine dose. Serum samples were stored at -80°C until use. The final group comprised a total of 45 mRNA-1273 and 29 BNT162b2 vaccine recipients, none of whom had a reported prior history of SARS-CoV-2 infection, with all having negative baseline (anti-nucleoprotein) serological testing.

Cells and viruses

We maintained HEK293T cells (ATCC CRL-11268) in adherent cell culture in Dubelcco’s modified Eagle’s medium (DMEM) (Thermo Fisher Scientific Cat# 11995073) supplemented with

10% (v/v) fetal bovine serum (FBS) and 1% (v/v) penicillin-streptomycin (Thermo Fisher Scientific Cat# 15140163). HEK293T cells were maintained in suspension cell culture in FreeStyle 293 Expression Medium (Thermo Fisher Scientific Cat# 12338026) containing 1% (v/v) penicillin-streptomycin. HEK293S GnTI$^{-/-}$ cells (ATCC CRL-3022) were also maintained in Freestyle 293 Expression medium supplemented with 2% (v/v) ultra-low IgG FBS (Thermo Fisher Scientific Cat# 16250078) and 1% (v/v) penicillin-streptomycin. Expi293F™ cells (Thermo Fisher Scientific Cat# A14527) were maintained in Expi293™ expression-medium (Thermo Fisher Scientific Cat# A1435102) that contained 1% (v/v) penicillin-streptomycin. We received a HEK293T stable cell line overexpressing human ACE2 (hACE2) as a gift from Huihui Mou and Michael Farzan. We maintained these cells in DMEM supplemented with 10% (v/v) FBS, 1% (v/v) penicillin-streptomycin and 1 μg ml^{-1} puromycin. We received a previously described Expi293F™ stable cell line that expresses His$_6$-tagged human ACE2 ectodomain (residues 1–615, including the endogenous single peptide) (*80*) as a gift from Bing Chen, and maintained these cells in Expi293™ expression-medium, supplemented with 1 μg ml^{-1} puromycin and 1% (v/v) penicillin-streptomycin. We confirmed the absence of mycoplasma in all cell lines monthly with an e-Myco PCR detection kit (Bulldog Bio Cat# 25234). All cell lines were obtained from commercial vendors except for the Expi293F-His$_6$-tagged ACE2, and HEK239T-hACE2 stable cell lines. Neutralization assays with infectious virus used passage 4 SARS-CoV-2 USA/WA1/2020 received from the University of Texas Medical Branch (*81*), as previously described (*14*).

<u>Protein production</u>

As a reagent for single B-cell sorting experiments and for ELISAs, we generated a human codon-optimized SARS-CoV spike protein construct (GenBank ID: ABD72979.1 residues 14–1177), which was stabilized in the prefusion conformation through the addition of proline substitutions at residues 968 and 969 (“S2P”) (*54*). We cloned this construct into a pHLsec vector (*82*) that has its own secretion signal sequence. To produce SARS-CoV S2P, we transfected Expi293F™ cells using ExpiFectamine™ (Thermo Fisher Scientific Cat# A14525), according to the manufacturer's protocol. We purified the protein from cell supernatants two to three days after transfection using an anti-FLAG M2 Affinity Gel (Sigma-Aldrich Cat# A2220) (RRID: AB_10063035), according to the manufacturer’s protocol. We removed the FLAG tag and His_6-tag by TEV protease digestion followed by reverse nickel affinity purification and size exclusion chromatography on a Superose 6 Increase column (GE Healthcare Life Sciences). We biotinylated the protein with BirA ligase as previously described (*14*).

To produce recombinant SARS-CoV-2 spike protein ectodomain for ELISAs and cryo-electron microscopy (cryo-EM) experiments, we constructed a human codon-optimized SARS-CoV-2 spike protein (GenBank ID: QHD43416.1, residues 1–1208) construct (“HexaPro”) that contains a “GSAS” substitution at the furin cleavage site (residues 682–685) and six stabilizing proline substitutions at positions 817, 892, 899, 942, 986, and 987, as previously described (*83*). We subcloned it into a pHLsec vector and included at the C-terminus a foldon trimerization motif followed by a BirA ligase site, a Tobacco Etch Virus (TEV) protease site and a His_6-tag. To produce the recombinant protein, we transfected HEK293T cells grown in suspension with linear polyethylenimine (PEI) MAX (Polysciences Cat# 24765-1). We purified the protein from supernatants four days after transfection by nickel affinity purification and digested the protein with TEV protease followed by a reverse-nickel affinity step to remove the protease. We used size-

exclusion chromatography on a Superose 6 Increase column to further purify the protein in buffer containing 200 mM NaCl, 20 mM Tris-HCl, pH 7.5.

For use in ELISAs and biolayer-interferometry (BLI) experiments, we cloned the SARS-CoV spike protein RBD (GenBank ID: AAP33697.1 residues 306–526), the SARS-CoV-2 spike protein RBD (GenBank ID: QHD43416.1 residues 319–541), and the day 146* spike protein and day 152* spike protein RBDs (*14*) into the pHLsec vector (*82*). For crystallography, the day 146* construct included an N-terminal His_6-tag, a TEV protease site, and a "SGSG" linker. For BLI and ELISA assays, constructs contained an N-terminal His_6-tag, a TEV protease site, a BirA ligase site, and a 7-residue linker (GTGSGTG). The SARS-CoV-2 day 146* RBD crystallography construct was produced by PEI MAX transfection of $GnTI^{-/-}$ HEK293S cells grown in suspension. Constructs used for ELISA and BLI studies were produced in HEK293T cells by PEI MAX transfection. We purified RBDs from supernatants by nickel affinity chromatography, digested RBDs with TEV protease to remove the His_6-tag and performed reverse nickel affinity purification. For BLI, we biotinylated proteins with BirA ligase as previously described (*14*), followed by reverse nickel affinity purification to remove the ligase. As a final step, we purified all recombinant RBDs by size exclusion on a Superdex 200 Increase column (GE Healthcare Life Sciences) in phosphate buffered saline (PBS).

To produce recombinant soluble mucin-like deleted Ebola virus (EBOV) glycoprotein (GP) for use as a negative control in ELISAs, we subcloned a human codon-optimized construct of EBOV GP (GenBank ID: NC_002549.1 residues 34–312 and 464–636) into the pHLsec expression vector (*82*) containing a Foldon motif at the protein C-terminus. We purified the supernatant five days after transfection on an affinity resin with mAb100 Fab (*84*) conjugated to a

Pierce NHS-Activated Agarose Slurry (Thermo Fisher Scientific Cat #26200), followed by size exclusion purification on a Superose 6 Increase column.

Expression constructs for antibodies REGN10933 and REGN10987 (*18*), CR3022 (*55*), and C1A-A6 were previously described (*14*). We also generated codon optimized cDNA for the following antibodies using publicly available sequences: S309 (PDB: 6WS6) (*44*); LY-CoV555 (PDB: 7L3N) (*37*); LY-CoV016/CB6 (PDB: 7C01) (*21*). We cloned antibody variable light and heavy chain gene regions for these antibodies or newly isolated C1 donor-derived SARS-CoV spike protein cross-reactive antibodies into pVRC8400 vectors to generate light chains, IgGs, or Fabs as previously described (*14*). We co-transfected heavy and light chain vectors in Expi293F™ cells using an ExpiFectamine™ transfection kit, according to the manufacturer's protocol. We performed affinity purification of IgGs using the MabSelect SuRE Resin (GE Healthcare Cat# 17547401) and Fabs using either the CaptureSelect™ KappaXL Affinity Matrix (Thermo Fisher Scientific Cat# 194321010) or the CaptureSelect™ LC-lambda (Hu) Affinity Matrix (Thermo Fisher Scientific Cat# 084905) to isolate Fabs by following the manufacturers' protocols. Following affinity purification, we isolated all Fabs by size exclusion chromatography on a Superdex 200 Increase column, and all proteins eluted at the expected retention volume as single peaks in PBS.

An Fc fusion protein containing the human ACE2 ectodomain (GenBank ID: BAB40370.1 residues 18–740) was expressed and purified as previously described (*14*). We produced recombinant human ACE2 ectodomain containing the $T55A_{ACE2}$ and $T92A_{ACE2}$ substitutions by subcloning the cDNA of human ACE2 (a gift from Michael Farzan) into a pHLsec vector (GenBank ID: BAB40370.1 residues 19–615) with Gibson assembly to insert codon mutations. Both constructs contain a C-terminal His_6-tag. Wild-type (WT) His_6-tagged human ACE2

ectodomain was purified from a stable cell line (a gift from Bing Chen) as previously described (*80*). We further purified each protein using size-exclusion chromatography on a Superdex 200 Increase column, in PBS (for proteins used in BLI assays) or 150 mM NaCl, 25 mM Tris-HCl, pH 7.5 (for proteins used in crystallization screens).

Single B-cell sorting and antibody cloning

We sorted single memory B cells as previously described (*14*) using a MoFlo Astrios EQ Cell Sorter (Beckman Coulter) at the Harvard Medical School Flow Cytometry Core Facility. Briefly, we isolated PBMCs from B cells by incubating them with anti-CD20 MicroBeads (Miltenyi Biotec Cat# 130-091-104) and subjecting them to magnetic separation on a MACS LS column (Miltenyi Biotec Cat# 130-042-401), according to the manufacturer's protocol. We resuspended B cells in PBS supplemented with 2% (v/v) FBS. We adjusted the density of B cells to $1x10^7$ cells ml^{-1} and incubated cells on ice for 30 min with biotinylated SARS-CoV S2P. We washed the cells three times with PBS containing 2% (v/v) FBS; added streptavidin-PE (Invitrogen Cat# S866), anti-CD19-FITC antibody (BD Biosciences Cat# 340864 RRID: AB_400152), and anti-IgG-APC antibody (BD Biosciences Cat# 550931 RRID: AB_398478); and incubated them for 30 minutes on ice. Following incubation, we washed cells three times in PBS containing 2% (v/v) FBS and passed them through a cell strainer, followed by sorting.

We performed single cell cDNA synthesis using SuperScript™ III reverse transcriptase (Invitrogen Cat# 18080044) followed by nested PCR amplification to obtain the IgH, Igλ, and IgK variable segments from memory B cells as previously described (*14*). We used IMGT/V-QUEST (*85*) (http://www.imgt.org) to analyze IgG gene usage and the frequencies of variable segment somatic hypermutation.

Protein crystallization

We formed complexes by mixing recombinant His_6-tagged human ACE2 ectodomain with 1.5 molar excess of SARS-CoV-2 day 146* RBD. We incubated the mixture overnight at 4°C and passed it on a HiLoad 16/600 Superdex 200 column (GE Healthcare Life Sciences) in 150 mM NaCl, 25 mM Tris-HCl, pH 7.5. The complex co-eluted as a single peak at the expected retention volume and we concentrated it to 12 mg ml^{-1}. We screened for crystallization conditions in hanging drops containing 0.1 µl protein and 0.1 µl mother liquor using a Mosquito protein crystallization robot (SPT Labtech) with commercially available screens (Hampton and Molecular Dimensions). Crystals grew within 72 h in 20% (v/v) PEG550 MME, 10% (w/v) PEG 20000, 20 mM 1,6-Hexanediol, 20 mM 1-Butanol, 20 mM 1,2-Propanediol, 20 mM 2-Propanol, 20 mM 1,4-Butanediol, 20 mM 1,3-Propanediol, and 0.1M Tris (base) BICINE pH 8.5.

We purified C1C-A3 Fab on a Superdex 200 Increase column in buffer containing 150 mM NaCl, 25 mM Tris-HCl, pH 7.5. We concentrated the Fab to 7 mg ml^{-1} and screened for crystallization conditions in hanging drops containing 0.1 µl Fab and 0.1 µl mother liquor using a Mosquito protein crystallization robot (SPT Labtech) with commercially available screens (Hampton and Molecular Dimensions). Crystals grew within 48 h in 18% (w/v) PEG 8000; 0.02 M Magnesium chloride hexahydrate; and 0.1 M Tris pH 8.9.

Lentivirus pseudotype production

Human codon-optimized SARS-CoV (GenBank ID: AAP33697.1 residues 14–1228) and human codon-optimized SARS-CoV-2 (GenBank ID: QJA16508.1 residues 1–1246) spike proteins were cloned into the pCAGGS expression vector and modified to include HIV gp41

residues (NRVRQGYS) as previously described (*14*), replacing C-terminal residues 1228–1225 or 1247–1273 for SARS-CoV or SARS-CoV-2, respectively. The cDNA encoding the SARS-CoV spike protein (a gift from Hyeryun Choe) includes a CD5 signal sequence. SARS-CoV-2 spike constructs for pseudotypes D614G_S, D614G_S/N439K_{RBD}, D614G_S/N501Y_{RBD}, D614G_S/Q493K_{RBD}, D614G_S/Q493R_{RBD}, day 146*, and day 152* were previously described (*14*). The day 146* spike protein is derived from hCoV-19/USA/MA-JLL-D146/2020 (EPI_ISL_593557), with WT (Wuhan-Hu-1 SARS-CoV-2 strain) sequences at positions 12–18, an NTD deletion spanning residues 142–144, and an additional Y489H_{RBD} mutation (observed at an early sequencing time point in the immunocompromised host) (*14, 15*). The day 152* spike protein is derived from the hCoV-19/USA/MA-JLL-D152/2020 strain (EPI_ISL_593558), with an additional Y489H_{RBD} mutation. We used Gibson assembly to generate additional spike protein variants used in this study: B.1.1.7 (Alpha), B.1.351 (Beta), P.1 (Gamma), B.1.427/429 (Epsilon), B.1.526 E484K or S477N (Iota E484K or S477N), B.1.617.1 (Kappa), RBM-1 or RBM-2. The B.1.1.7 (Alpha) sequence was obtained from hCoV-19/England/205261299/2020 (EPI_ISL_754289). The B.1.351 (Beta) sequence was obtained from hCoV-19/South Africa/Tygerberg-461/2020 (EPI_ISL_745186). The P.1 (Gamma) sequence was obtained from hCoV-19/Brazil/AM-FIOCRUZ-20143138FN-R2/2020 (EPI_ISL_811149). The B.1.427/429 (Epsilon) sequence was obtained from hCoV-19/USA/CA-COLOR-UCSF-CC353/2021 (EPI_ISL_1825082). The B.1.526 E484K (Iota E484K) sequence was obtained from hCoV-19/USA/NY-CDC-2-4242372/2021 (EPI_ISL_1823970). The B.1.526 S477N (Iota S477N) sequence was obtained from hCoV-19/USA/NY/NYCPHL-004382/2021 (EPI_ISL_1804950). The B.1.617.1 (Kappa) sequence was obtained from hCoV-19/India/KA-NIMH-SEQ-280/2021 (EPI_ISL_1595896). The Delta AY.2 sequence was obtained from hCoV-19/USACA-LACPHL-AH01469/2021

(EPI_ISL_2907577). The B.1.617.2 E484K N501Y F490S (Delta +3) sequence was obtained from hCoV-19/Turkey/HSGM-FS976/2021 (EPI_ISL_3403341). The C.37 (Lambda) sequence was obtained from hCoV-19/Chile/MA-CADIUMAG-00881/2021 (EPI_ISL_2907514). RBM-1 is derived from the hCoV-19/USA/MA-JLL-D146/2020 strain (EPI_ISL_593557), with WT sequences at positions 440 and 1020, and the $E484K_{RBD}$ substitution. RBM-2 bears the RBM-1 mutations with the $K417N_{RBD}$ substitution. RBM-3 is derived from the B.1.351 (Beta) sequence, originating from the hCoV-19/South Africa/Tygerberg-461/2020 strain (EPI_ISL_745186), with the additional $N439K_{RBD}$ and $L452R_{RBD}$ substitutions. The RaTG13 virus (GenBankID: QHR63300.2, residues 1–1242) spike protein was cloned into the pCAGGS expression vector and modified to include HIV gp41 residues (NRVRQGYS), replacing C-terminal residues 1243–1269 for RatG13 virus. A pCAGGS expressor plasmid for VSV G was previously described (*14*). Spike protein lentivirus pseudotypes were packaged, harvested, filtered, and concentrated, with storage at -80°C, as previously described (*14*).

Pseudotype neutralization experiments

We pre-incubated SARS-CoV-2 spike protein variants or VSV G lentivirus pseudotypes with serial dilutions of polyclonal human sera, monoclonal antibodies, or an ACE2-Fc fusion protein in the presence of DMEM supplemented with 0.5 μg ml^{-1} of polybrene for 1 hour at 37°C. We added pseudotype-antibody mixtures to monolayers of HEK293T cells overexpressing human ACE2 for 24 hours at 37°C and then replaced the media with DMEM supplemented with 10% (v/v) FBS, 1% (v/v) penicillin-streptomycin, and 25 mM HEPES. We performed FACS 48 hours after initial infection with an iQue3 Screener PLUS (Intellicyt) to determine the percent of GFP positive cells. We calculated percent relative entry and percent neutralization using the following

equations: (1) Relative entry (%) = (%GFP-positive cells in the presence of antibody / %GFP in the absence of antibody) x 100%; (2) Neutralization (%) = [1 - (%GFP-positive cells in the presence of antibody / %GFP in the absence of antibody)] x 100%.

For dose response neutralization assays, we generated response curves with a 4-parameter nonlinear function and reported titers as the antibody concentration required to achieve 50% neutralization [50% inhibitory concentration (IC_{50} value) for monoclonal antibodies or 50% inhibitory dilution (ID_{50} value) for serum] in GraphPad Prism v9.0.1. For serum samples, the input dilution of serum used was 1:20, which represents the lower limit of quantification for this assay. Samples that did not achieve 50% neutralization are expressed as <20 and plotted at half of that dilution (1:10). Each experiment was performed twice independently in triplicate (*n*=6). To calculate the mean fold change between each variant pseudotype and the $D614G_S$ pseudotype, the average of the six ID_{50} data points was calculated for WT and the variant pseudotype. In the case where sample replicates returned ID_{50} values both >20 and <20, the result is reported as 1:15. We then calculated the mean fold change using the equation: Mean Fold Change = - (WT $D614G_S$ Mean Titer / Variant Mean Titer).

Live virus PRNT experiments

Neutralization assays with C1C-A3 and C1A-A6 IgG and infectious SARS-CoV-2 [passage 4 SARS-CoV-2 USA/WA1/2020 (*81*)] were performed as previously described (*14*).

ELISA experiments

We coated NUNC MaxiSorb plates (Thermo Fisher Scientific Cat# 44-2404) with SARS-CoV-2 HexaPro soluble spike protein ectodomain, SARS-CoV S2P soluble spike protein

ectodomain, SARS-CoV-2 RBD, SARS-CoV RBD, or control proteins soluble Ebola virus surface glycoprotein lacking the mucin like domain or Lujo virus GP1 (generated as previously described) (*14*) overnight at 4°C. We blocked the antigen with PBS containing 3% BSA and 0.02% (v/v) Tween. We added the monoclonal antibodies to the plates for one hour at room temperature at the indicated concentrations. We washed the plates three times with PBS containing 0.02% (v/v) Tween followed by two washes with PBS alone. We detected bound antibodies with anti-human-Fc-HRP (Sigma-Aldrich Cat# A0170) using a Synergy Neo2 HTS Multi-Mode Microplate Reader (BioTek).

<u>Biolayer interferometry experiments</u>

We used an Octet RED 96e (Sartorius) for all BLI experiments. For affinity measurements, we loaded biotinylated SARS-CoV RBD, SARS-CoV-2 RBD, or SARS-CoV-2 variant RBDs onto a streptavidin (SA) sensor (ForteBio) at a concentration of 1.5 μg ml^{-1} for 100 s in kinetic buffer (0.02% Tween and 0.1% BSA in PBS). After a 60 s baseline measurement in kinetic buffer, antibody Fabs or recombinant human ACE2 ectodomain were associated for 300 s followed by a 300 s dissociation step in kinetic buffer. We used ForteBio data analysis software with a 1:1 binding model to determine kinetics of binding.

For competition experiments, we loaded biotinylated SARS-CoV-2 RBD onto SA sensors for 100 s at 1.5 μg ml^{-1}. We associated the antibody Fab or the ACE2-Fc fusion protein for 180 seconds at 250 nM followed by association of the second Fab or the ACE2-Fc fusion protein for 180 s at 250 nM. For competition assays with antibody S309, we used an IgG instead of a Fab. Fabs or the ACE2-Fc fusion protein were deemed to be competing if there was no or little change in refractive index following association of the second protein.

X-ray crystallography

We flash froze crystals in mother liquor with 15% (v/v) glycerol added as a cryoprotectant. We collected single crystal X-ray diffraction data on Eiger X 16M pixel detectors (Dectris) at a wavelength of 0.97918 Å at the Advanced Photon Source (APS, Argonne, IL) NE-CAT beamline 24-ID-C. Diffraction data were indexed and integrated using XDS (build 202 00131) (*69*) and merged using AIMLESS (v0.5.32) (*70*). We generated a Fab model using the heavy and light chains of PDB codes 4DGV and 4PTU, respectively, with flexible loops trimmed. We used the constant and variable domains of this Fab as search models to determine the structure of the C1C-A3 Fab (space group $P12_11$; one Fab per asymmetric unit) by molecular replacement using Phaser (v2.8.3) (*86*). The day 146* RBD:ACE2 complex structure (space group $P2_12_12_1$; two copies per asymmetric unit) was determined by molecular replacement using ACE2 and the SARS-CoV-2 RBD from PDB code 6M0J (*2*) as search models. We performed iterative model building in program O 15.1.0 (*87*) or *Coot* v0.9.5 (*72*) and refinement in Phenix v1.18.2-3874 (*73*) and Buster v2.10.3 (*71*), during which we also built alternative conformations for residue side chains where density was apparent. During refinement, we updated TLS groups calculated using Phenix v1.18.2-3874 (*73*) and a python script, as well as occupancy restraints calculated in Buster v2.10.3 (*71*). During model building, we also customized geometry restraints to prevent large displacement of unambiguous contacts in poor regions; the restraints were released once refinements became stable. Water molecules were automatically picked and updated in Buster, followed by manual examination and adjustment until late-stage refinement. Data collection, processing and refinement statistics are summarized in table S4. Final models were validated using MolProbity (*88*).

SARS-CoV-RBD deglycosylation

Recombinant SARS-CoV RBD (R&D Systems Cat# 10583-CV) was deglycosylated by treating with Rapid™ PNGase F (New England Biolabs Cat# P0710S) at a ratio of 1:10 (enzyme:protein) for five hours at 37°C with gentle mixing. The intact molecular weight of deglycosylated and glycosylated proteins were then analyzed using a 6538 Q-TOF attached to a 1290 UPLC system (Agilent Technologies) equipped with a BioResolve RP mAb Polyphenyl column (2.1 X 100mm, Waters). Material was eluted from the column using a linear gradient of 15% (v/v) to 50% (v/v) mobile phase B over 17 min at 250 μl min^{-1}, in which Mobile phase A was 0.1% (v/v) form acid (FA), 0.01% (v/v) trifluoroacetic acid (TFA) in water and mobile phase B was 0.1% (v/v) form acid, 0.01% (v/v) trifluoroacetic acid in acetonitrile (ACN). Q-TOF acquisition parameters were: 350°C gas temperature, OCT/RF at 750 V, mass range from 600–3200 m/z and a collection rate of 1.03 spectra/s. Mass Hunter Qualitative Analysis software (Agilent Technologies) was used to deconvolute the observed sample molecular weight, which was 32,308.4 Da for the unmodified SARS-CoV RBD and 25,948.7 Da for the deglycosylated SARS-CoV RBD. Observed masses were compared to the expected masses as calculated from the protein sequence using General Protein Mass Analysis (GPMAW). Deglycosylated and glycosylated samples were analyzed on a Dionex Ultimate 3000 system (Thermo Fisher Scientific) equipped with a Superdex-200 column (10/300L) (GE Healthcare Life Sciences). Samples were passed onto the column in PBS and eluted at the expected elution volumes for their expected sizes with only minor changes in percent aggregation (8%) after removal of N-linked glycans.

Identification of an RBD N-linked glycans in public data bases

To identify additional sites for glycans that could potentially be introduced into the SARS-CoV-2 RBD, we searched genome sequences in the GISAID database (this included 2,487,354 sequences at the time of analysis on July 26th, 2021) for substitutions that introduce an asparagine while a serine or threonine is present at the +2 position (without a proline present at the +1 position) or substitutions at the +2 position of an asparagine that make it a candidate for N-linked glycosylation (*89*). We restricted our analysis to occurrences of at least 10 in GISAID. This analysis identified the following substitutions: $D364N_{RBD}$, $A372S_{RBD}$, and $A372T_{RBD}$. GISAID accession numbers and acknowledgements to sequence depositors are provided in table S8 and table S9.

Antibody affinity measurements using surface plasmon resonance

Surface plasmon resonance measurements were made using a Biacore T200 instrument, with Biacore T200 control software v4.0.1 (Cytiva Inc). Experimental data from the Biacore T200 instrument was analyzed using Biacore evaluation software v4.1 (GE Healthcare Life Sciences). Goat antibodies specific to the Fc region of human IgG (Invitrogen Cat# 31125) were covalently linked to the carboxy methyl dextran matrix of CM5 biosensor chips (GE Healthcare Life Sciences Cat# BR-1006-68) using free amine groups with an amine coupling kit (GE Healthcare Life Sciences Cat# BR-1000-50). Carboxyl groups of the dextran matrix on the chip were activated with 100 mM N-Hydroxysuccinimide (NHS) and 400 mM of 1-Ethyl-3-(3-dimethylaminopropyl) carbodiimide hydrochloride (EDC). Goat anti-human IgG Fc (25 μg ml^{-1}), diluted in 10 mM sodium acetate, pH 4.5, was injected across all four of the activated flow cell surfaces. Once the level of binding response reached the desired value, unreacted groups were deactivated by injection of 1 M Ethanolamine. Approximately 9000–10000 response units (RU) of goat anti-

human IgG Fc were immobilized on each of the four active flow cell surfaces. C1C-A3 and C1A-A6 were diluted to 1 μg ml^{-1} in HBS-EP 1x [10 mM HEPES buffer pH 7.4 containing 150 mM NaCl, 3 mM Ethylenediaminetetraacetic acid (EDTA), 0.005% (v/v) P20] and injected over the goat anti-human IgG Fc surfaces, at a flow rate of 10 μl min^{-1} for 60 s, to achieve a capture level of 128–186 RU. The net difference in the baseline signal and the signal after the completion of the antibody injections were taken to represent the amount of captured monoclonal antibodies. The antigen binding experiments consisted of antigen association and antigen dissociation phases. Aliquots of unmodified SARS-CoV RBD (R&D Systems Cat# 10583-CV) and deglycosylated SARS-CoV RBD were injected at different concentrations, at a flow rate of 50 μl min^{-1}, for 5 min over the captured antibodies to ascertain association rates. SARS-CoV RBDs were tested at the following concentrations: 0.41, 1.23, 3.70, 11.11, 33.33, 100, and 300 nM. The dissociation phase consisted of continued flow of HBS-EP at 50 μl min^{-1} for 10 min. Reaction surfaces were regenerated with 20 μl injections of 10 mM glycine pH 1.5 at a flow rate of 50 μl min^{-1}. The reference surface response, with no captured antibody, was subtracted from the reaction surface data to eliminate changes in the refractive index and injection noise. We fit the data using a 1:1 Langmuir binding model in the BiaEvaluation software (GE Healthcare Life Sciences).

Glycopeptide analysis by mass spectrometry

To evaluate whether the $A372S_{RBD}$ substitution allows for N-linked glycosylation of $N370_{RBD}$, we ordered for synthesis a DNA construct comprising WT RBD residues 319–540 of the SARS-CoV-2 spike protein (GenBank ID: YP_009724390.1), a human IgG heavy chain signal peptide, and a C-terminal "GGSGGS" linker followed by a His_{10} tag, a "GSG" linker, an AVI tag, and a His_6 tag (Genewiz). Plasmid encoding WT RBD was mutagenized by site directed

mutagenesis to introduce the A372S$_{RBD}$ substitution. The recombinant protein was produced by transient transfection of HEK293-6E cells (NRC Canada) using PEI-MAX (Polysciences Inc Cat# 24765) transfection. Twenty-four hours post-transfection cells were fed with Tryptone-N1 (5 g L^{-1}) (OrganoTechnie Cat# 19553), and supernatants were harvested six days post-transfection. We purified the RBD from supernatants by nickel affinity chromatography using HisTrap Excel columns (Cytiva Cat# 17371206), followed by size exclusion on a HiLoad 26/600 Superdex 200 pg in PBS. The protein eluted as a single peak at the expected elution volume [97% homogeneity when analyzed on a Superdex S200 Increase column (Cytiva Cat# 28990945)]. SARS-CoV-2 RBD containing the A372S$_{RBD}$ substitution was first denatured in 6 M guanidine hydrochloride, 100 mM Tris pH 8.0 for 15 min at room temperature. The denatured sample was then reduced with 10 mM DTT and alkylated with 50 mM iodoacetic acid (Sigma) at 37°C for 30 min. The reduced and alkylated sample was then desalted and exchanged into 50 mM Tris, pH 8.0 using Zeba Protein Desalt Spin Cartridges (Thermo Fisher Scientific). The sample was then divided into three aliquots and digested separately with sequencing grade trypsin, chymotrypsin, and LysC-Glu-C at a 1:20 w/w protease: protein ratio at 37°C overnight, after which the digestion was quenched by lowering the pH with formic acid. The resulting peptides were analyzed by nanoLC-ESI MS with an Easy-nLC 1200 (Thermo Fisher Scientific) system coupled to an Orbitrap Fusion LUMOS mass spectrometer (Thermo Fisher Scientific). Peptides were separated by PepMap RSLC C_{18} column (75 μm × 75 cm) with an in-line trapping column (PepMap 100 C_{18} 3 μM 75 μM x 2 cm) prior to the analytical column. Peptides were eluted with a linear gradient of 5% to 35% mobile phase B over 65 min at 200 nl min^{-1}, where mobile phase A was 0.1% (v/v) formic acid in water and mobile phase B was 0.1% (v/v) formic acid in acetonitrile. The spray voltage was set to 3 kV and capillary temperature at 275°C. MS1 scans were performed over 600–1500 m/z at an Orbitrap resolution of

120,000 and selected precursors for MS2 with charge states 2–7 and dynamic exclusion for 30 s. Standard AGC target was used with a maximum injection time of 250 s and HCD collision energy of 30%.

Glycopeptide identification

Byonic software v3.11 (Protein Metrics Inc.) was used for the data analysis and identification of the glycan compositions associated with the detected glycopeptides. The precursor mass tolerance was set at 10 ppm, 20 ppm for fragments, and FDR=1%. The high confidence glycopeptides search criteria was set at a minimum score ≥ 200 and a delta mod score ≥ 100. The variable modification included oxidation (Met), deamidation (Asn) and fixed modification for carbamidomethyl (Cys). The mass spectrometry data were searched using the Protein Metrics 309 N-glycan library. The extracted-ion chromatogram (XIC) areas from MS1 were used for quantitation of the glycopeptides and the different glycoforms having an identical peptide sequence. Glycans were categorized and classified according to a report by Watanabe and colleagues (*90*). Spotfire software was then used to summarize the overall glycosylation complexity and heterogeneity at each site as well as across the entire protein (fig. S13B).

Cryo-EM sample preparation and data collection

We applied 3 µl of sample containing 0.6 mg ml^{-1} of SARS-CoV-2 spike protein ectodomain (HexaPro) (*83*) and 0.3 mg ml^{-1} of the C1C-A3 Fab (1:1.5 spike protein to Fab molar ratio), which had been pre-mixed and incubated for 5 min at room temperature, to Cu 1.2/1.3 2 nm carbon film 300 mesh grids (EMS Catalog# Q3100CR1.3-2nm) that had been previously glow discharged in a PELCO easiGlow™ system. Grids containing samples were vitrified with a

Vitrobot Mark IV with a blotting time of 4–6 s at 100% relative humidity. Data were collected on a Thermo Fisher Titan Krios operating at 300 kV with a post-GIF energy filter (20 eV), equipped with a Gatan K3 camera. We collected 4583 dose-fractionated images, each with a total dose of 56.5 ($e^-/Å^2$) divided into 50 frames over a 1.9 s exposure. The defocus range used was -1.0 to -2.5 µm.

Cryo-EM structure determination

Initial image processing was carried out using RELION 3.1 (*91*). Movie frames were gain-normalized and motion-corrected using MotionCor2 (*74*). Defocus was calculated using CTFFind4.1 (*75*), as implemented in RELION 3.21. We picked particles using crYOLO v1.7.6 (*76*) with a general model for low-pass filtered images. We processed 1,296,929 picked particles in cryoSPARC v2.14.2 (*77*) and RELION 3.1 (*91*). We discarded particles that yielded poor 2D class images (fig. S10). We generated an *ab initio* model with 3 classes in cryoSPARC and chose one class containing 509,866 particles for homogenous refinement followed by heterogenous refinement with five classes. Two classes containing a total of 344,920 particles from heterogenous refinement were further refined with CTF refinement, resulting in a final map of 3.1 Å resolution. We also calculated a similar map of 4.0 Å resolution in RELION 3.1 following 3D classification and auto-refinement. Due to flexibility in the RBD and C1C-A3 Fab regions, the local resolution was poor in both maps. We thus extracted RBD:C1C-A3 subparticles from the trimer particle images using alignment parameters and trimer geometry for local subparticle classification (fig. S10, right panels). A total of 218,112 subparticles were generated from the final set of 72,704 trimer particle images. Two of the five subparticle classes containing a total of 106,285 particles

were combined to compute a final map of 4.0 Å resolution. The final map revealed continuous density that allowed us to trace the full RBD:C1C-A3 Fab model, described as follows.

We then used UCSF Chimera v1.13.1 to fit the crystal structure of the C1C-A3 Fab (PDB: 7SN1) and the SARS-CoV-2 RBD (PDB: 7KFV) (*14*) into maps obtained from subparticle classification and performed rigid body refinement in Phenix (v1.19-4080) (*73*). The resulting coordinates served as a starting model for iterative manual rebuilding in program O (v15.1.0) (*87*) with real space refinement in Phenix (v1.19-4080) (*73*) until convergence. We applied secondary structure restraints throughout refinement. We generated geometry restraints based on the identified key contacts in the converged model and applied the restraints with Ramachandran restraints for the final rounds of refinement. We followed similar strategies to build and refine the trimeric SARS-CoV-2 spike protein:C1C-A3 Fab model. Briefly, we used UCSF Chimera v1.13.1 to fit the spike trimer from PDB: 7KNI (*92*) into the trimer map and created the full trimer model by superposing three copies of the RBD:C1C-A3 Fab model (PDB: 7SN2) onto the RBD. Minor adjustments were necessary to ensure proper fitting of most model segments into the density. The rigid bodies of the composite model were refined in Phenix (v1.19-4080) (*73*) before iterative manual building and real space refinement. Phenix validation tools were used to guide the entire building and rebuilding processes. We trimmed parts of the model that were associated with poor map density prior to the final rounds of real space refinement and deposition of atomic coordinates. Final models were validated using MolProbity (*88*). The data processing strategy is shown in figure S10, and the data collection, model refinement, and validation statistics are provided in table S6. To aid in visualization of the full antibody Fab pose on the trimeric spike protein ectodomain as shown in Figure 4a, we superposed three copies of the RBD:C1C-A3 model (PDB: 7SN2) to the trimer model (PDB: 7SN3) based on RBD:C1C-A3 Fv alignment.

Antibody accessible surface area calculations

To provide a quantitative analysis of antibody accessibility and epitope preference due to the complex structure and extensive glycosylation of the SARS-CoV-2 spike protein, we calculated the antibody accessible surface area (AbASA) (*62*) for each residue in the RBD based on molecular dynamics (MD) simulations of the SARS-CoV-2 spike protein. The 3 μs MD trajectory used for the analysis, previously described in Grant et al. (*62*) and available from GLYCAM-Web (https://www.glycam.org), is based on the atomic coordinates of the prefusion-stabilized SARS-CoV-2 spike protein (PDB: 6VSB) (*29*). The glycoform of the SARS-CoV-2 spike protein in the model is representative of the data obtained from protein expressed in HEK293 cells (*90*). AbASA was computed for each residue in the RBD at each frame using the Python interface of FreeSASA (*93*) with the Lee & Richards algorithm (*94*) and using a probe radius of 7.2 Å. Values were averaged over the trajectory and a smoothing procedure was applied, consisting in a seven-point moving window procedure, according to the formula (*95*):

$$N_i=[7N_i+6(N_i-1+N_i+1)+3(N_i-2+N_i+2)-2(N_i-3+N_i+3)]/21$$

To estimate the effect of a N-linked glycan attached to SARS-CoV-2 $N370_{RBD}$, corresponding to $N357_{RBD}$ in the SARS-CoV spike protein, we used a 2 μs MD trajectory of the SARS-CoV spike protein RBD (*96*). The trajectory, based on the atomic coordinates PDB: 2AJF (*97*), is available from the COVID-19 Molecular Structure and Therapeutics Hub (https://covid.molssi.org). Briefly, the SARS-CoV RBD was superimposed frame by frame with the SARS-CoV-2 spike protein RBD, and the N-linked glycan attached to $N357_{RBD}$ was included in the AbASA calculation.

Structural analysis of RBD-targeting antibodies

To model the ADG-2 RBD complex shown in in Figure S11, we used ColabFold AlphaFold2 w/ MMseqs2 (*98*) to generate a model for the ADG-2 Fab using the antibody variable heavy (GenBank ID: MZ439266.1) and light chain (GenBank ID: MZ439267.1) protein sequences (*45*) linked with human CH_1 heavy chain and lambda light chain constant region protein sequences. We then fit the ADG-2 Fab model into cryo-EM maps of a prefusion-stabilized SARS-CoV-2 spike protein bound to the ADG-2 Fab (5.94 Å, EMD-23160) (*45*) using UCSF Chimera (v1.13.1), placing the Fab density near the open RBD, followed by manual adjustments. Although the pose of its Fab differs somewhat from other class 1 antibodies and its binding site sits in between most class 1 and class 4 antibodies (Movie 1), we classified ADG-2 as a "class 1-like" antibody because its RBD footprint overlaps with the ACE2 binding site and based on our structural modeling ADG-2 would only bind the spike protein RBD when it is in the open conformation. We used PyMOL (v2.4) (Schrödinger) to analyze structures and generate figures.

Quantification and statistical analysis

Data and statistical analyses were performed using Prism (v9.0.1) (GraphPad). Flow cytometry data was analyzed using IntelliCyt ForeCyt Standard Edition (v8.1.7524) (Sartorius). Biolayer-interferometry data were analyzed using Data Analysis HT (v12.0.1.55) (Sartorius). Statistical details for experiments can be found in figures and figure legends, including the statistical test used, p-value for statistical significance, exact value of n, and what n represents.

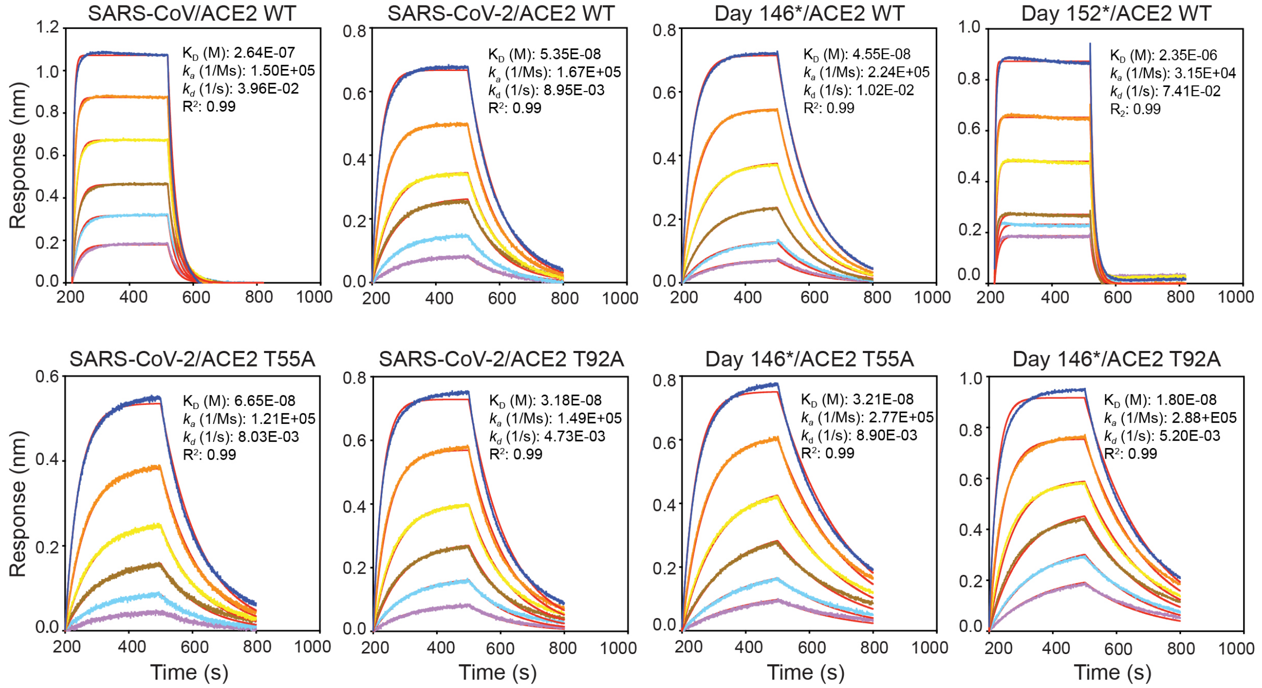

Fig. S1. Sensorgrams for biolayer interferometry-based affinity measurements of ACE2 ectodomain RBD binding affinities. SARS-CoV and SARS-CoV-2 RBD affinities for wild-type (WT) ACE2 or the indicated ACE2 mutants were determined by biolayer interferometry (BLI). Red lines represent the fit for a 1:1: binding model, and alternate colors represent response curves measured at varying concentrations. Binding affinities were determined for six concentrations of ACE2 at twofold dilutions ranging from 1 µM to 31.3 nM (SARS-CoV RBD/ACE2 WT), 125 nM to 3.9 nM (SARS-CoV-2 RBD/ACE2 WT, SARS-CoV-2 RBD/ACE2 T55A, SARS-CoV-2 RBD/ACE2 T92A, day 146* RBD/ACE2 WT, day 146* RBD/ACE2 T55A, day 146* RBD/ACE2 T92A), and 10 µM to 312.5 nM (day 152* RBD/ACE2 WT). Each experiment was performed at least twice, and representative data are shown. Biolayer interferometry data are summarized in table S3.

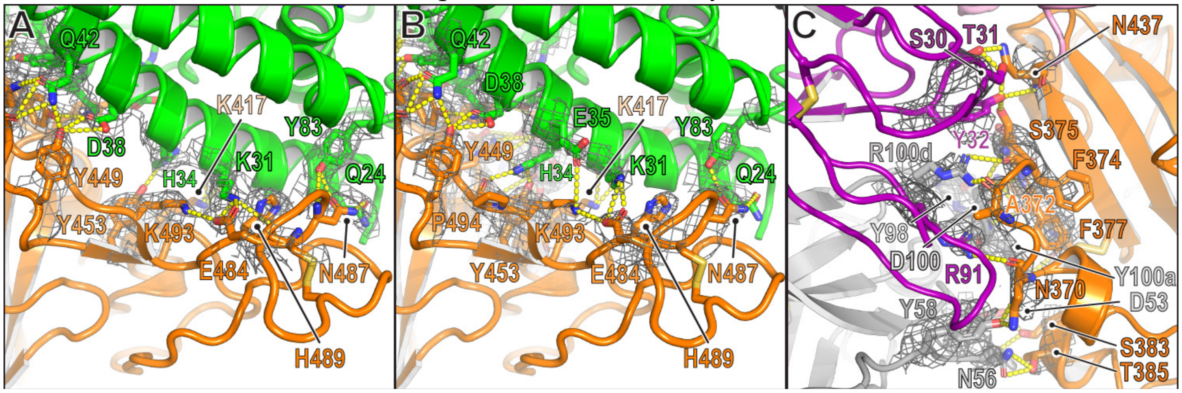

Figure S2. Examples of side chain density at the interfaces for reported structures. (**A**) Side chain electron density for one copy in the asymmetric unit of the X-ray crystal structure of the day 146* RBD complexed with the human ACE2 ectodomain at part of the receptor-glycoprotein interface. (**B**) Side chain density for the second copy in the asymmetric unit of the day 146* RBD/human ACE2 ectodomain crystal structure at part of the receptor-glycoprotein interface. (**C**) Density at the Fab/RBD interface for the cryo-EM structure of the C1C-A3 Fab bound to the SARS-CoV-2 spike protein ectodomain after local refinement.

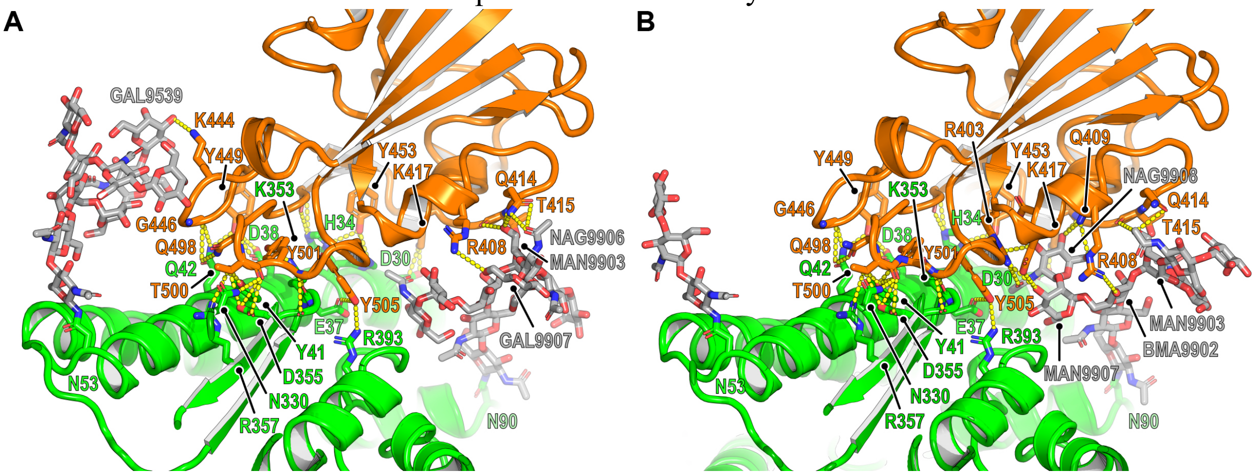

Fig. S3. ACE2 glycans contacting the day 146* RBD. (**A**) Overview of the day 146* RBD interactions with human ACE2 highlighting contacts N-linked glycans attached to $N53_{ACE2}$ and $N90_{ACE2}$ make with the RBD. One of the two complexes of the crystal asymmetric unit is shown. (**B**) Same view as in (A), but with the other complex of the crystal asymmetric unit.

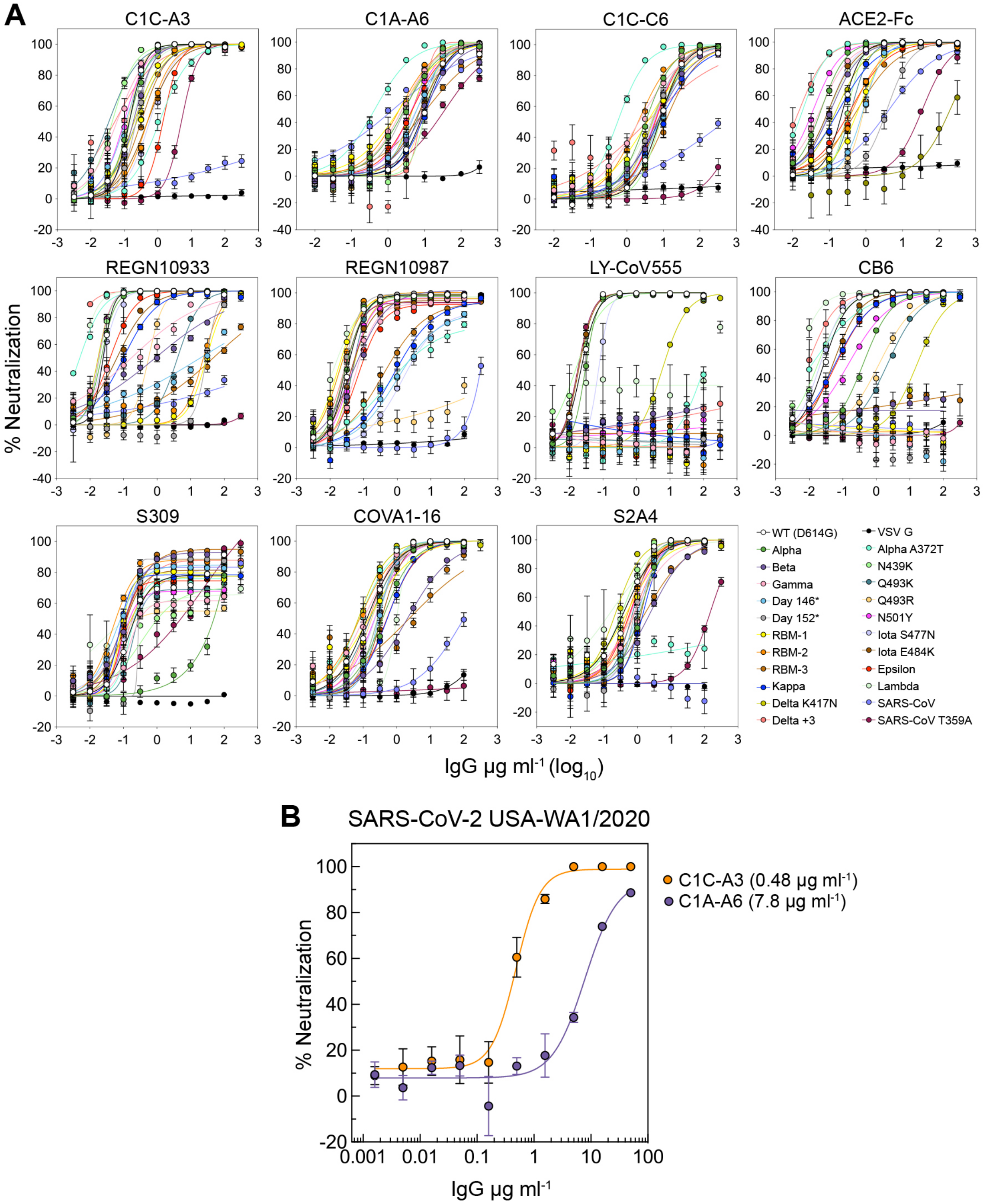

Fig. S4. Neutralization profiles of monoclonal antibodies against spike protein lentivirus pseudotypes and infectious SARS-CoV-2. (**A**) Neutralization of lentivirus pseudotypes by the indicated monoclonal antibodies as measured with HEK293T cells overexpressing human ACE2. (**B**) Neutralization IC_{50} dose response curves of antibodies C1C-A3 and C1A-A6 against infectious SARS-CoV-2 USA/WA1/2020 measured through a plaque reduction neutralization test on Vero E6 cells. IC_{50} values are listed in parentheses. Data are shown as mean +/- standard deviation of the mean for experiments performed twice in triplicate (A) (*n*=6) or three experiments performed in triplicate (B) (*n*=9). In (A) and (B), for some data points, error bars are smaller than symbols.

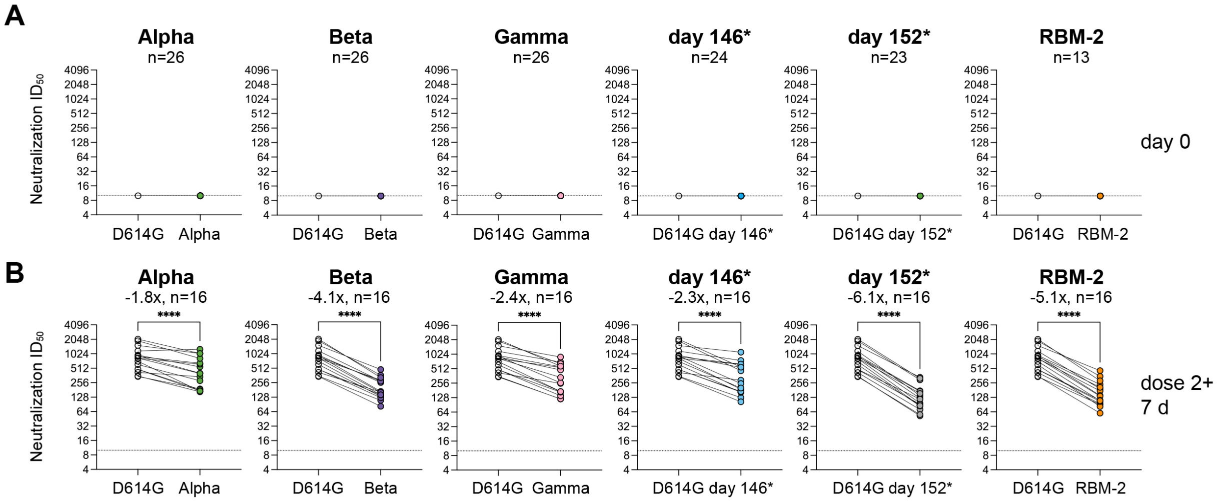

Fig. S5. Neutralization of spike protein lentivirus pseudotypes by mRNA vaccine-elicited serum. (**A**) Baseline mean ID_{50} neutralization titers for the indicated spike protein variant pseudotypes at day 0. (**B**) Mean neutralization ID_{50} values for indicated spike protein pseudotypes seven days following the second immunization (Wilcoxon matched-pairs signed rank test) (****indicates p-value < 0.0001). Baseline samples were not available for testing all donors in this study, but no donors reported a prior history of SARS-CoV-2 infection, and all had baseline negative serological testing (anti-nucleoprotein). Total donor group included 45 mRNA-1273 and 29 BNT162b2 vaccine recipients. Each experiment was performed twice independently in triplicate (*n*=6).

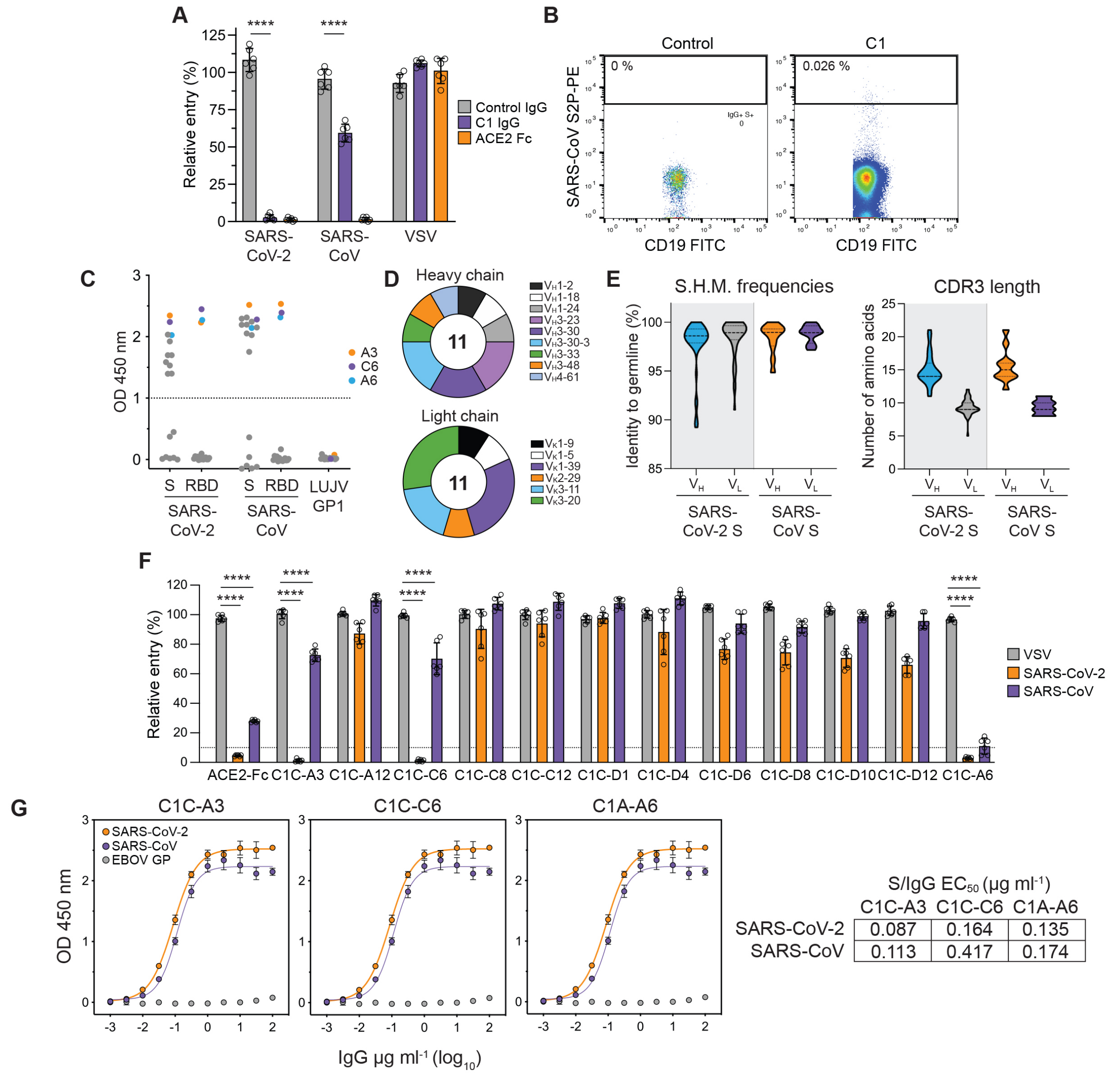

Fig. S6. Isolation profile of SARS-CoV spike-protein reactive antibodies recovered from a COVID-19 convalescent individual. (**A**) Percent relative entry levels of SARS-CoV-2, SARS-CoV, or vesicular stomatitis virus (VSV) lentivirus pseudotypes after pre-incubation with polyclonal immunoglobulins (IgG) purified from the plasma of a COVID-19 convalescent individual ("C1"), a non-immune control donor ("Control IgG"), or an ACE2-Fc fusion protein (all at a single concentration of 316 μg ml^{-1}). Data are normalized to a no antibody control and are plotted as mean +/- standard deviation of the mean from two experiments performed in triplicate (*n*=6) (One-way ANOVA with Tukey's multiple comparisons test) (**** indicates p-value < 0.0001). (**B**) Density plots from a FACS experiment to sort memory B cells that bind phycoerythrin (PE)-labeled streptavidin tetramers coupled to a prefusion-stabilized SARS-CoV spike (S) protein construct (S2P-PE). The box denotes the approximate location of the sorting gate, with the percentage of cells that fall within that gate indicated. The left panel shows FACS data from a control donor, while the right panel is for donor C1. (**C**) Whisker plot of ELISA values for IgG binding to SARS-CoV-2 spike protein (S) or RBD, SARS-CoV S or RBD, or a control protein

Lujo virus (LUJV) GP1 (all antibodies were tested at a single concentration of 100 μg ml^{-1}). Dots above the dashed line indicate the antibodies that bind each respective protein. C1C-A3 ("A3"), C1C-C6 ("C6"), and C1A-A6 ("A6") are specifically labeled. (**D**) Antibody heavy and light chain gene usage for 11 total SARS-CoV spike protein reactive monoclonal antibodies isolated from C1. (**E**) Violin plots of somatic hypermutation frequencies (S.H.M.) and CDR3 loop lengths for spike protein-reactive monoclonal antibodies. The quartiles and median are depicted as dotted and dashed lines, respectively. Previously published data illustrating S.H.M. and CDR3 loop lengths for SARS-CoV-2 spike protein reactive monoclonal antibodies isolated from C1 are highlighted in gray in each figure (*14*). (**F**) Single concentration neutralization assays were performed to determine whether each indicated monoclonal antibody or an ACE2-Fc fusion protein neutralized SARS-CoV-2, SARS-CoV, or VSV lentivirus pseudotypes at a screening concentration of 100 μg ml^{-1}. The dashed line indicates 10% relative entry. Data are plotted as mean +/- standard deviation of the mean from two experiments performed in triplicate (n=6), with all data normalized to a no antibody control. (One-way ANOVA with Tukey's multiple comparisons test) (**** indicates p-value < 0.0001). (**G**) ELISAs were performed to determine the half maximal effective concentration (EC_{50}) of A3, C6, and A6 IgGs binding to SARS-CoV-2 RBD, SARS-CoV RBD, or a control protein Ebola virus (EBOV) GP. Each experiment was performed twice in triplicate (n=6). EC_{50} values are summarized in the table. For some data points, error bars are smaller than symbols.

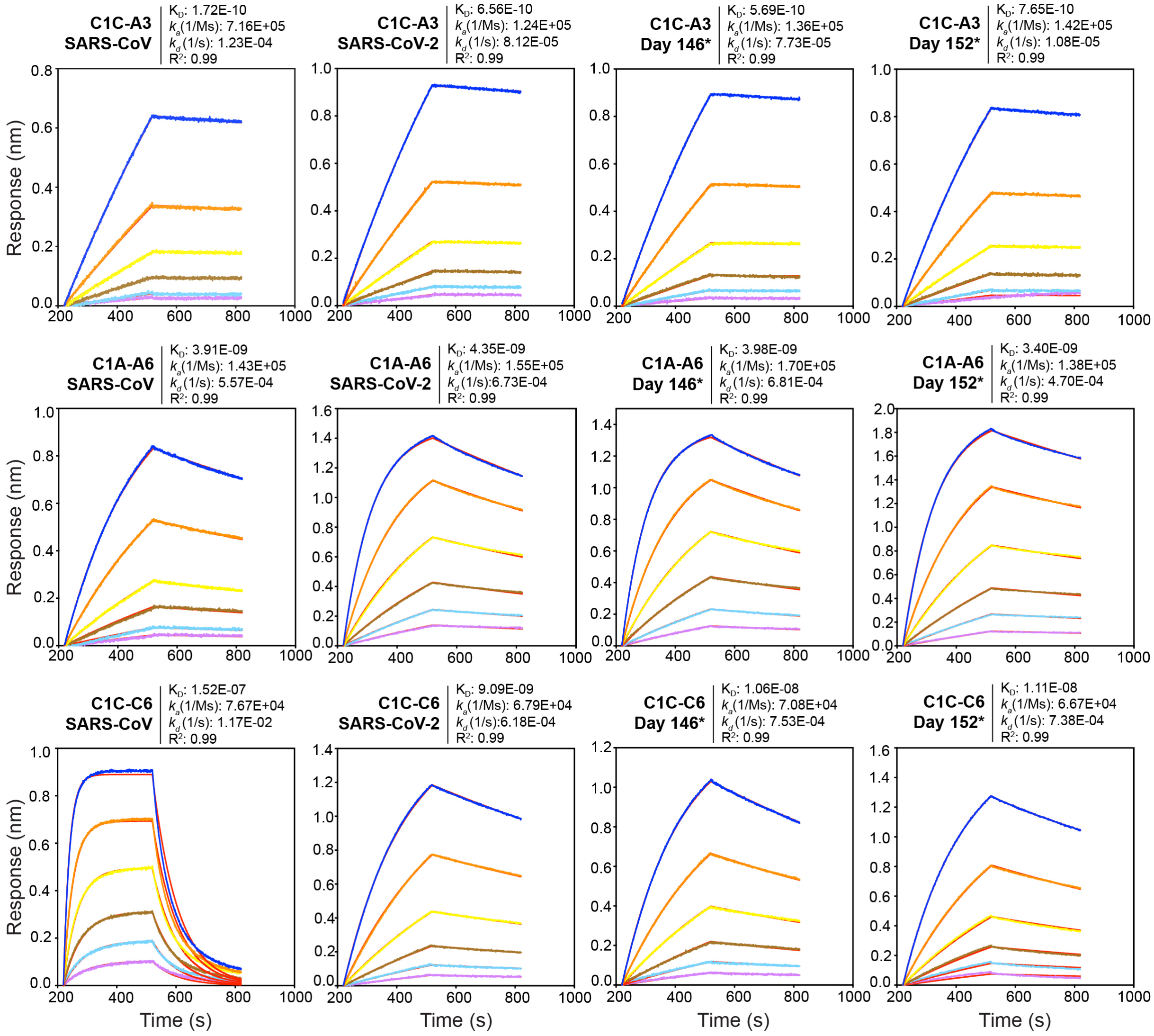

Fig. S7. Biolayer interferometry sensorgrams for antibody Fab RBD affinity measurements. Antibody Fab affinities for RBDs immobilized to sensor tip surfaces were determined by biolayer interferometry (BLI). Red lines represent the fit for a 1:1: binding model, and alternate colors represent response curves measured at varying concentrations. Assays were carried out using six concentrations of Fabs at twofold dilutions with a starting concentration of 500 nM (C1C-C6 with SARS-CoV RBD), 62.5 nM (C1A-A6 with SARS-CoV-2 RBD, C1A-A6 with day 146* RBD, C1A-A6 with day 152* RBD, C1C-C6 with SARS-CoV-2, C1C-C6 with day 146* RBD, C1C-C6 with day 152* RBD), 25 nM (C1A-A6 with SARS-CoV RBD), or 12.5 nM (C1C-A3 with all RBDs). Each experiment was performed at least twice, and representative data are shown. Biolayer interferometry data are summarized in table S3.

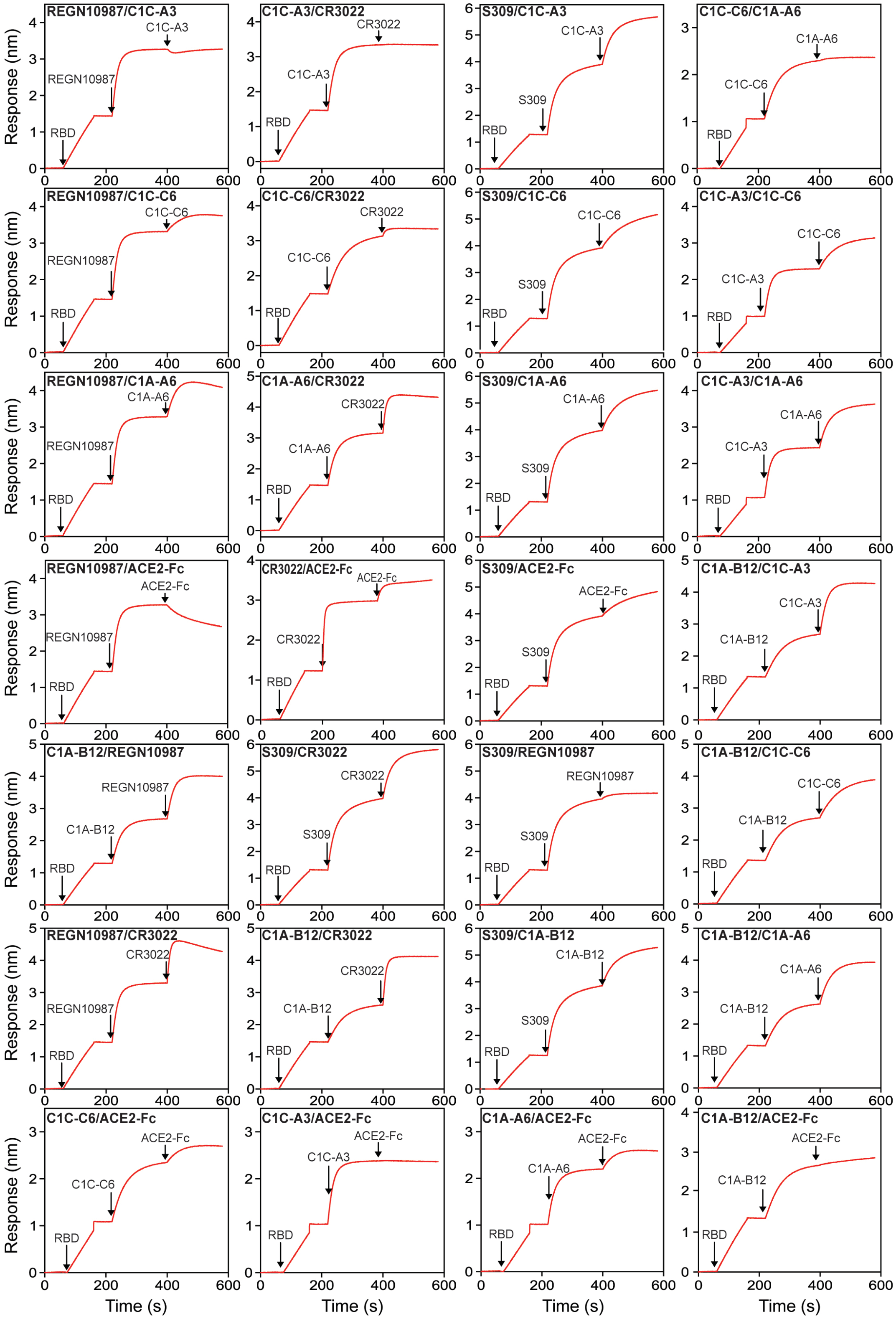

Fig. S8. Antibody competition assay. Biolayer interferometry-based competition assay for antibody Fabs (C1C-A3, C1C-C6, C1A-A6, C1A-B12, REGN10987, CR3022), S309 IgG, and a

human ACE2-Fc fusion protein with immobilized SARS-CoV-2 RBD. Arrows show the time point at which the indicated protein was added. Representative results for an experiment that was performed twice are shown. The results are summarized in Fig. 3B.

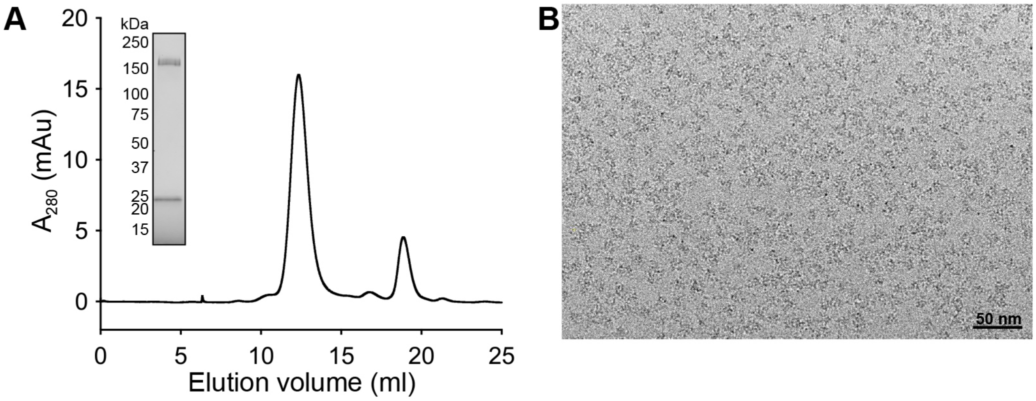

Fig. S9. Size exclusion chromatography trace and cryo-EM micrograph of a C1C-A3 Fab complexed with the SARS-CoV-2 spike protein ectodomain. (**A**) Size exclusion chromatography trace of prefusion stabilized SARS-CoV-2 spike protein ectodomain (HexaPro) (*83*) complexed with the C1C-A3 Fab. Inset is an SDS-PAGE gel of the main (first) peak fraction under reducing conditions. The Fab forms a stable complex with the spike protein ectodomain. kDa: kilodalton. (**B**) Motion corrected cryo-EM micrograph of vitrified samples of the complex obtained after pre-mixing of the C1C-A3 Fab with the spike protein ectodomain. The data processing strategy for cryo-EM structure determination is provided in figure S10.

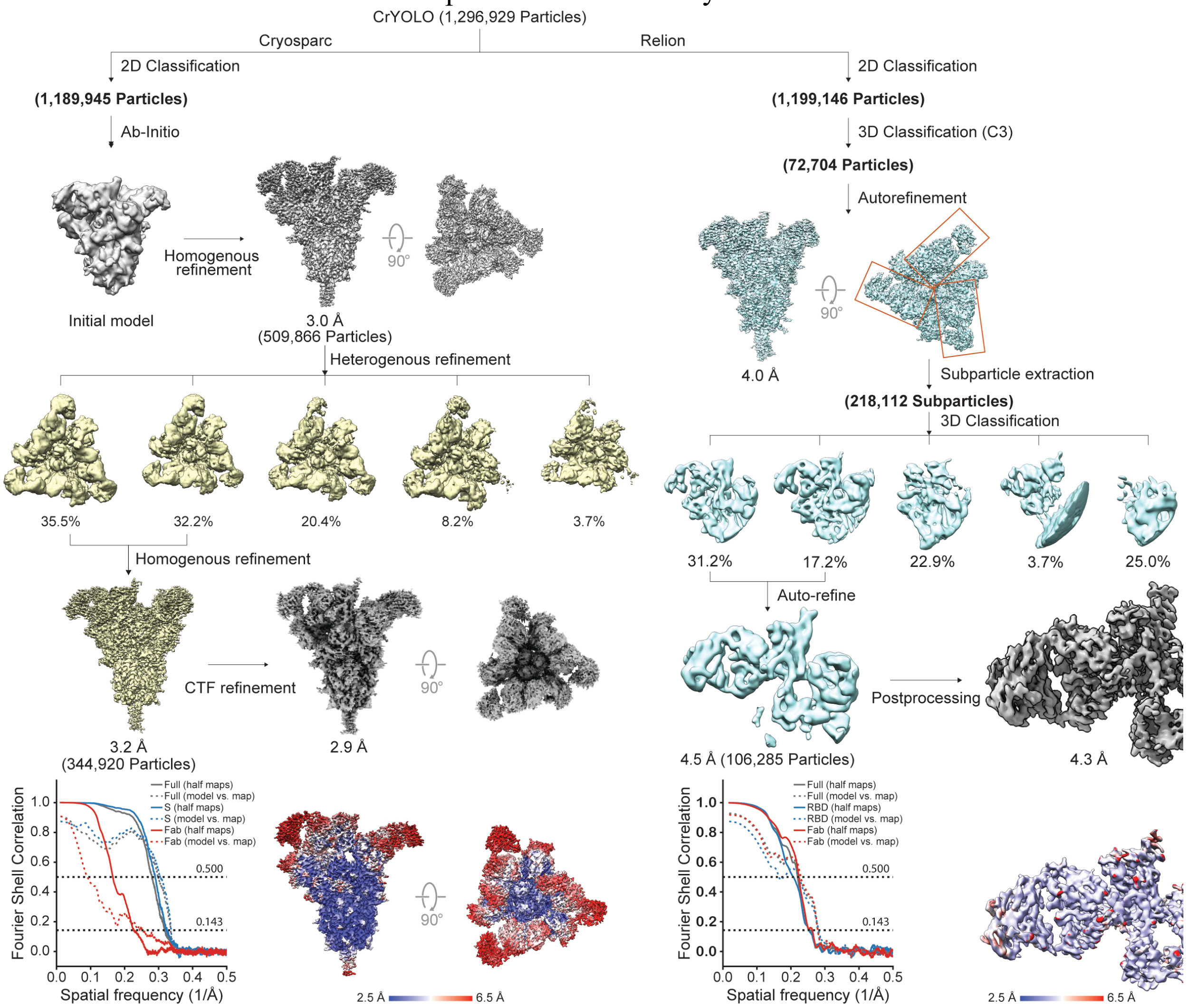

Fig. S10. Data processing strategy for cryo-EM structure determination of the C1C-A3 Fab complexed with the SARS-CoV-2 spike protein ectodomain. Additional information is provided in Materials and Methods.

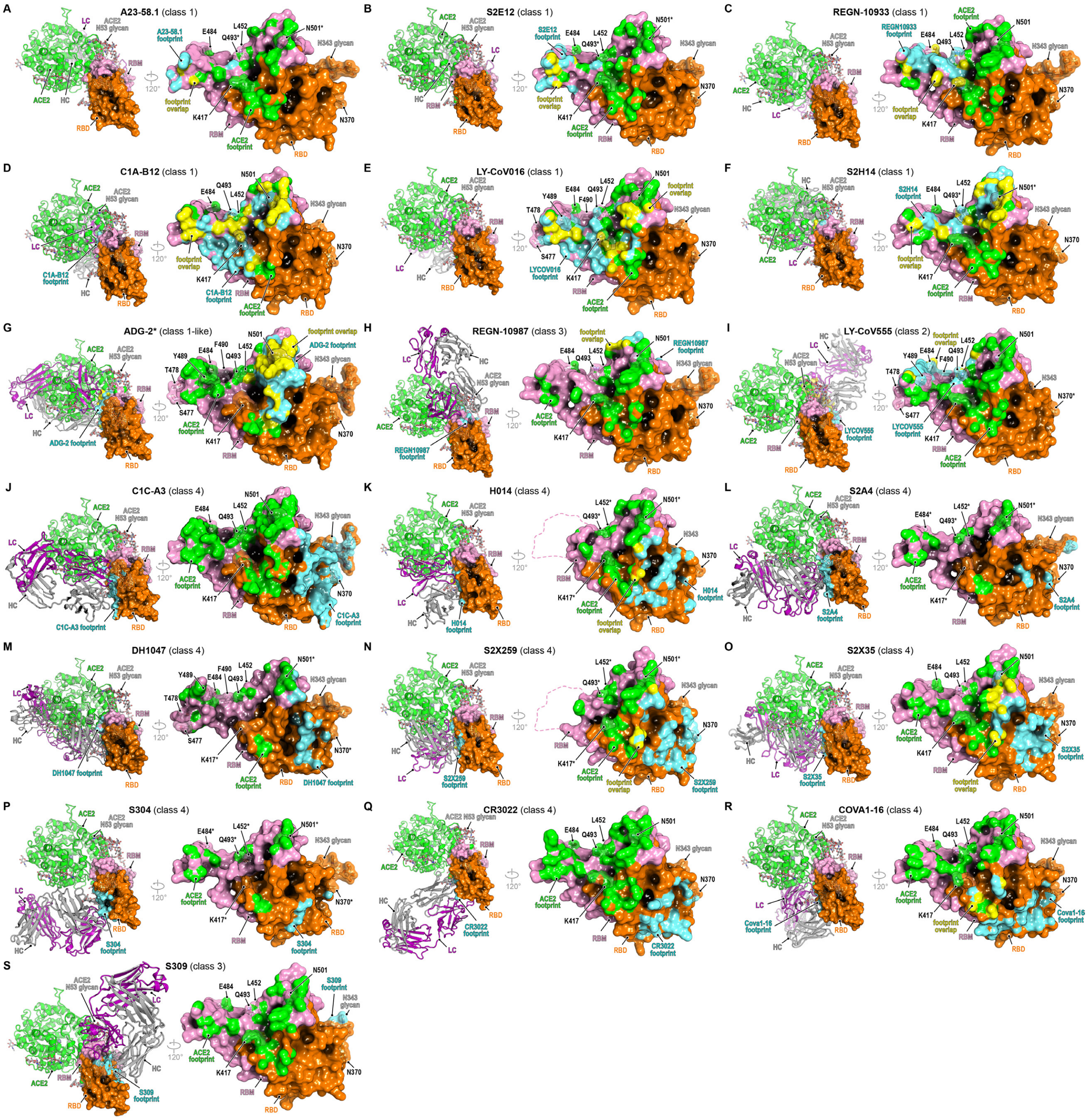

Fig. S11. RBD footprint of monoclonal antibodies and relationship to the ACE2 binding site. Fabs of V_H/V_L fragments for the indicated antibodies are shown in ribbon diagram and the RBD is shown in surface representation with antibody and ACE2 footprints labeled as indicated. Key residues that are substituted among variants discussed in the main text are also indicated. For left subpanels, the structure of the day 146* RBD/ACE2 ectodomain complex is superposed to highlight antibody steric clashes with ACE2 as well as to facilitate comparison with the binding mode of class 4 antibodies, which principally target the RBD core but not the RBM. Because of the angle, Fabs or V_H/V_L fragments for some antibodies that bind the ACE2 footprint are partially obscured. Residues built as alanine in models are indicated with an asterisk. Antibodies are

classified according to Barnes *et al*. (*36*). (**A**) A23-58.1, a class 1 antibody (PDB: 7LRS) (*42*). (**B**) S2E12, a class 1 antibody (PDB: 7K45) (*99*). (**C**) REGN10933, a class 1 antibody (PDB: 6XDG) (*18*). (**D**) C1A-B12, a class 1 V_H3-53-derived neutralizing antibody (PDB: 7KFV) (*14*). (**E**) LY-CoV016 (CB6), a class 1 antibody (PDB: 7C01) (*21*). (**F**) S2H14, a class 1 antibody (PDB: 7JX3) (*4*). (**G**) ADG-2 (*45*), a "class 1-like" antibody for which structural coordinates were not available but were modeled using low resolution cryo-EM maps of prefusion-stabilized SARS-CoV-2 spike bound to ADG-2 Fab (see Materials and Methods for further details). (**H**) REGN10987, a class 3 antibody (PDB: 6XDG) (*18*). (**I**) LY-CoV555, a class 2 antibody (PDB: 7L3N) (*37*). (**J**) C1C-A3, a class 4 antibody described in this study. (**K**) H014, a class 4 antibody (PDB: 7CAH) (*58*). (**L**) S2A4, a class 4 antibody (PDB: 7JVC) (*4*). (**M**) DH1047, a class 4 antibody (PDB: 7LD1) (*59*). (**N**) S2X259, a class 4 antibody (PDB: 7RAL) (*56*). (**O**) S2X35, a class 4 antibody (PDB: 7R6W) (*60*). (**P**) S304, a class 4 antibody (PDB: 7JW0) (*4*). (**Q**) CR3022, a class 4 antibody (PDB: 6W41) (*55*). (**R**) COVA1-16, a class 4 antibody (PDB: 7JMW) (*57*). (**S**) S309, a class 3 antibody (PDB: 6WPS) (*44*).

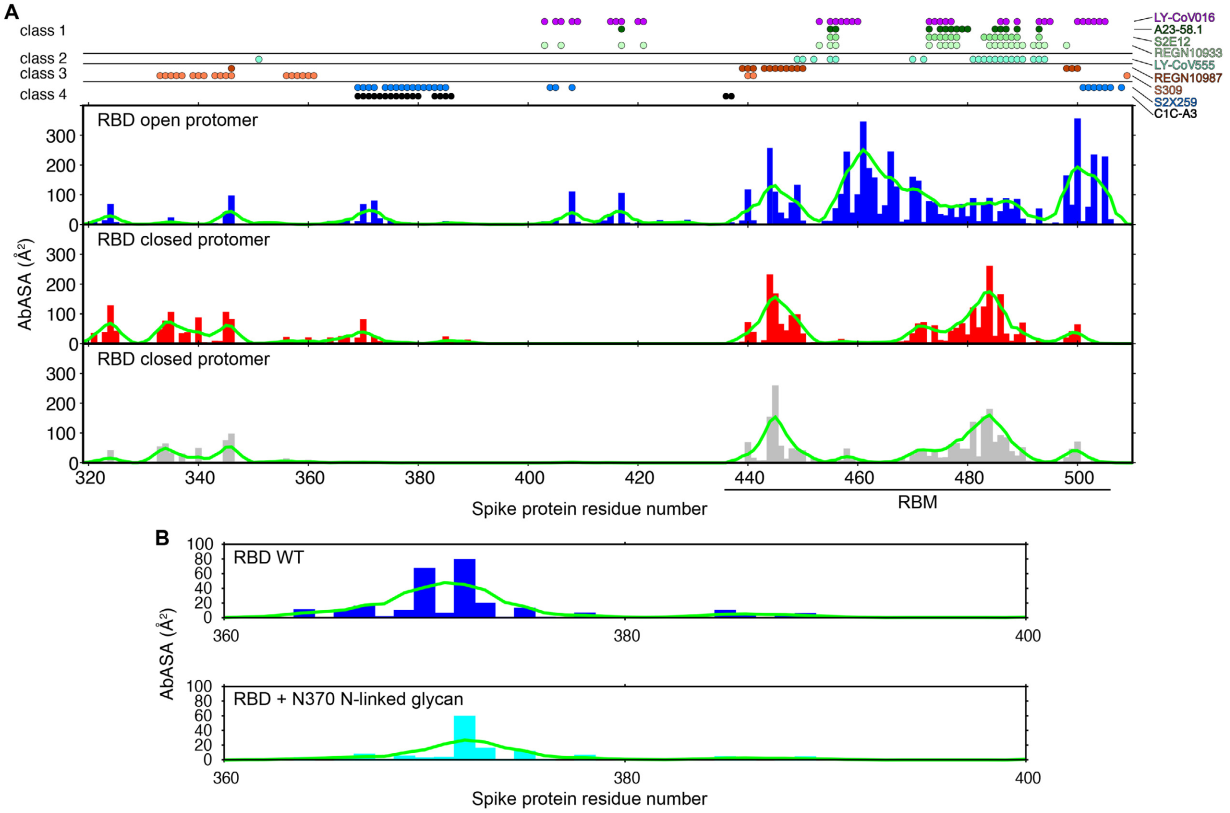

Fig. S12. Antibody accessibility surface area calculations for glycosylated SARS-CoV-2 spike protein. (**A**) Antibody accessible surface area (AbASA) profiles for the RBDs of the SARS-CoV-2 spike protein trimer as calculated with the trimer in the one open RBD and two closed RBDs state, which is thought to be a predominant form on the surface of virions (*100*). The AbASA values were calculated for each frame in the trajectory and averaged. The plots show the results for each of the protomers of the trimer, where the given RBD is in the open or closed state, as indicated. A smoothing procedure was applied to each profile, shown as a green line, according to the formula: $N_i=[7N_i+6(N_{i-1}+N_{i+1})+3(N_{i-2}+N_{i+2})-2(N_{i-3}+N_{i+3})]/21$ (*95*). RBD contact residues for the indicated antibodies are shown as circles on top of the plot and were determined using the respective atomic coordinates: LYCoV-016 (CB6) PDB: 7C01 (*21*), A23-58.1 PDB: 7LRS (*42*), S2E12 PDB: 7K45 (*99*), REGN10933 PDB 6XDG (*18*), REGN10987 PDB: 6XDG (*18*), S309 PDB: 6WPS (*44*), S2X259 PDB: 7RAL (*56*). Antibodies are grouped using the classification scheme described by Barnes *et al*. (*36*). (**B**) The AbASA values were calculated for each frame in the trajectories and averaged. The plots show the results for the open RBD without (top) or with an N-linked glycan (bottom) attached to $N370_{RBD}$ modelled onto the SARS-CoV-2 spike protein. A smoothing procedure was applied to each profile, shown as a green line, as described in *(A)*. Additional details are provided in the Materials and Methods section.

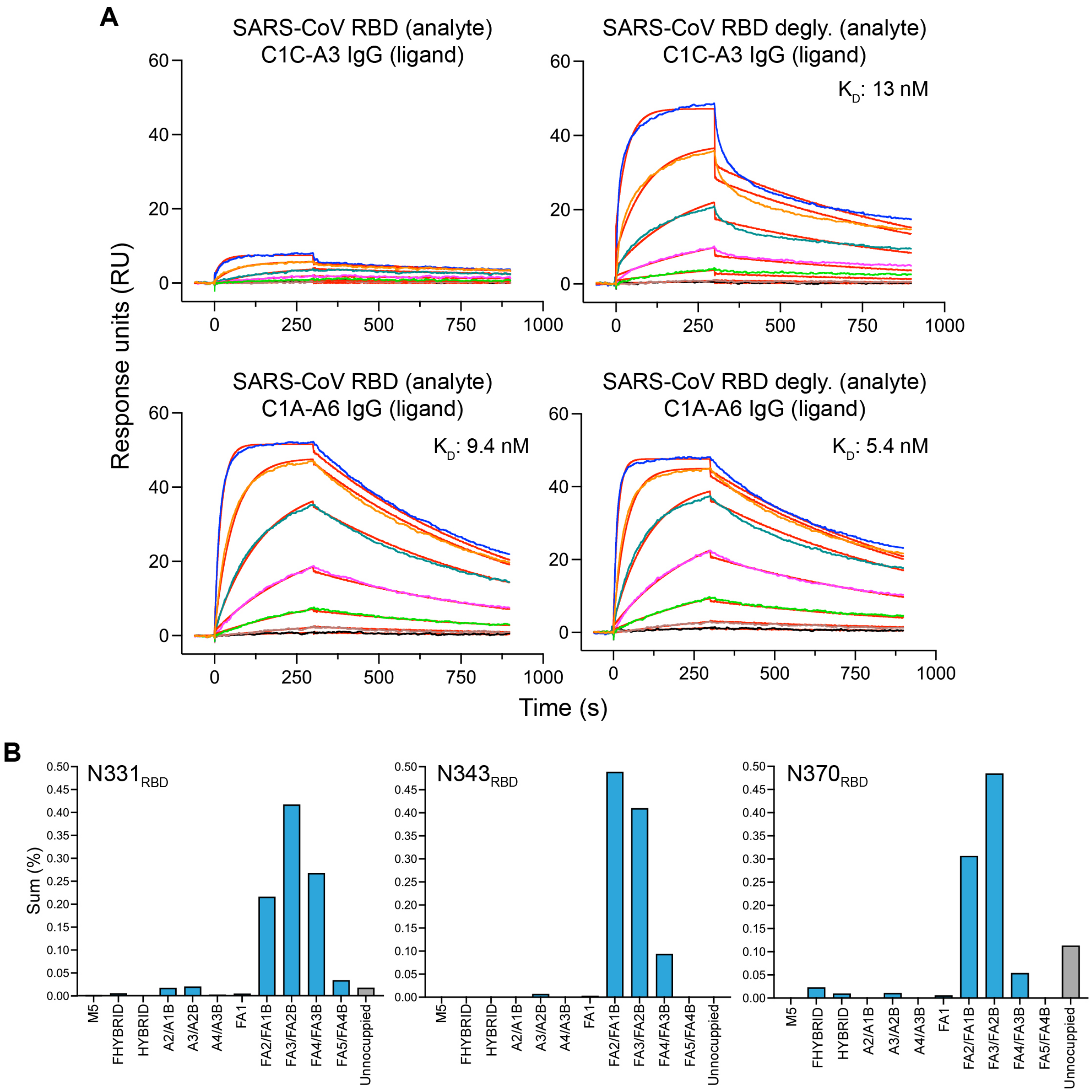

Fig. S13. Surface plasmon resonance sensorgrams for antibody binding to unmodified and deglycosylated SARS-CoV RBD and glycan analysis of A372S mutant RBD. (**A**) RBD affinities for the indicated IgG immobilized to sensor chip surfaces were determined by surface plasmon resonance. Red lines represent the fit for a 1:1: binding model, and alternate colors represent response curves measured at varying concentrations (0.41, 1.23, 3.70, 11.11, 33.33, 100, and 300 nM). Degly: deglycosylated RBD. Kinetic parameters of binding are provided in table S7. (**B**) N-linked glycans on recombinant RBD containing the A372S$_{RBD}$ substitution were categorized by mass spec analysis and classified according to a report by Watanabe and colleagues (*90*). The percent of glycan type at each position is shown.

Table S1. Human-derived SARS-CoV-2 sequences containing key RBD mutations discussed in the main text.

Virus name	PANGO lineage	Accession No.	RBD mutations of interest	Location	Collection date	Originating laboratory	Submitting laboratory	Authors
hCoV-19/England/205261299/2020	B.1.1.7	EPI_ISL_754289	N501Y	Europe / United Kingdom / England	2020-12-20	Respiratory Virus Unit, National Infection Service, Public Health England	COVID-19 Genomics UK (COG-UK) Consortium	PHE Covid Sequencing Team
hCoV-19/South Africa/Tygerberg-461/2020	B.1.351	EPI_ISL_745186	N501Y, E484K, K417N	Africa / South Africa / Western Cape / Cape Town Metro	2020-12-07	Wallacedene Clinic wc WAL	National Health Laboratory Service (NHLS), Tygerberg	Susan Engelbrecht, Kayla Delaney, Bronwyn Kleinhans, Houriiyah Tegally, Eduan Wilkindon, Gert van Zyl, Wolfgang Preiser, Tulio de Oliveira
hCoV-19/USA/NY-CDC-2-4242372/2021	B.1.526	EPI_ISL_1823970	E484K	North America / USA / New York / New York City	2021-04-01	NYC Department of Health and Mental Hygiene	Centers for Disease Control and Prevention Division of Viral Diseases, Pathogen Discovery	Mili Sheth, Sarah Nobles, Jasmine Padilla, Mark Burroughs, Shoshona Le, Katie Dillon, Peter Cook, Clinton R. Paden, Dhwani Batra, Krista Queen, Kristen Knipe, Dakota Howard, Yvette Unoarumhi, Darlene Wagner, Matthew Schmerer, Ben L. Rambo-Martin, Kristine Lacek, Sam Shepard, Alison Laufer Halpin, Dave Wentworth, Vivien Dugan, Suxiang Tong, Justin Lee
hCoV-19/USA/NY-NYCPHL-004382/2021	B.1.526	EPI_ISL_1804950	S477N	North America / USA / New York / New York City	2021-04-12	DOHMH Chelsea	New York City Public Health Laboratory	Jade Wang, et al.
hCoV-19/India/KA-NIMH-SEQ-280/2021	B.1.617.1	EPI_ISL_1595896	L452R, E484Q	Asia / India / Karnataka	2021-03-19	Kempegowda International Airport	INSACOG-KA, NIMHANS	Chitra Pattabiraman, Pramada Prasad, Anson Kunjumon George, Ananthapadmanabha Kotambail, Darshan Sreenivas, Chetan G K, Gautham Arunachal Udupi, Anita S Desai, V Ravi
hCoV-19/India/TS-CDFD-14963/2021	B.1.617.2	EPI_ISL_2897940	L452R, T478K	Asia / India / Telangana / Mahabubnagar	2021-05-22	Government Medical College	CDFD	Murali Bashyam, Asmita Gupta, Reelina Basu, Vinay Donipadi, Nagamani Kammili, Divya Vashisht, Ashwin Dalal
hCoV-19/USA/CA-LACPHL-AF01469/2021	B.1.617.2-like	EPI_ISL_2907577	L452R, T478K, K417N	North America / USA / California / Los Angeles County	2021-06-25	Martin Luther King Jr. Community Hospital	Los Angeles County Public Health Laboratories	P. Hemarajata et al
hCoV-19/USA/CA-COLOR-UCSF-CC363/2021	B.1.429	EPI_ISL_1825082	L452R	North America / USA / California / San Francisco County	2021-03-26	Color Genomics	Chiu Laboratory, University of California, San Francisco	Charles Chiu, Venice Servellita, Candace Wang, Alicia Sotomayor-Gonzalez, Yueyuan Zhang, Daniel Wong, Scott Topper, Madyson Wanner, Erika Torres, Alicia Zhou

hCoV-19/Brazil/AM-FIOCRUZ-20143138FN-R2/2020	P.1	EPI_ISL_811149	N501Y, E484K, K417T	South America / Brazil / Amazonas / Manaus	2020-12-30	Laboratorio de Ecologia de Doencas Transmissiveis na Amazonia, Instituto Leonidas e Maria Deane - Fiocruz Amazonia	Laboratorio de Ecologia de Doencas Transmissiveis na Amazonia, Instituto Leonidas e Maria Deane - Fiocruz Amazoni	Valdinete Nascimento, Victor Souza, André Corado, Fernanda Nascimento, George Silva, Ágatha Costa, Debora Duarte, Luciana Gonçalves, Matilde Mejía, Karina Pessoa, Maria Júlia Brandão, Michele Jesus, Felipe Naveca on behalf of the Fiocruz COVID-19 Genomic Surveillance Network
hCoV-19/Chile/MA-CADIUMAG-00881/2021	C.37	EPI_ISL_2907514	L452Q, F490S	South America / Chile / Magallanes / Punta Arenas	2021-06-28	Laboratorio de Medicina Molecular, Universidad de Magallanes	Centro Asistencial Docente y de Investigacion, Universidad de Magallanes	Constanza Ceroni, Diego Alvarez, Jorge González, Jacqueline Aldridge, Inés Cid, Roberto Uribe-Paredes, Marcelo Navarrete
hCoV-19/Angola/CERI-KRISP-K013439/2020	B.1.351	EPI_ISL_2493037	N501Y, E484K, K417N, L452R	Africa / Angola / Luanda	2020-12-09	Instituto Nacional de Investigação em Saúde	CERI, Centre for Epidemic Response and Innvoation, Stellenbosch University and KRISP, KZN Research Innovation and Sequencing Platform, UKZN.	Morais J, Neto Z, Afonso P, Miranda J, David K, Inglês L, Pereira A, Paulo A Carralero RR Paixão JP, Freitas RH, Mufinda M, Lutucuta S, Giandhari J, Pillay S, Naidoo Y, Emmanuel SJ, Tegally H, Wilkinson E, de Oliveira T
hCoV-19/Angola/CERI-KRISP-K013444/2020	B.1.351	EPI_ISL_2493036	N501Y, E484K, K417N, L452R	Africa / Angola / Luanda	2020-12-09	Instituto Nacional de Investigação em Saúde	CERI, Centre for Epidemic Response and Innovation, Stellenbosch University and KRISP, KZN Research Innovation and Sequencing Platform, UKZN	Morais J, Neto Z, Afonso P, Miranda J, David K, Inglês L, Pereira A, Paulo A Carralero RR Paixão JP, Freitas RH, Mufinda M, Lutucuta S, Giandhari J, Pillay S, Naidoo Y, Emmanuel SJ, Tegally H, Wilkinson E, de Oliveira
hCoV-19/Angola/CERI-KRISP-K013455/2020	B.1.351	EPI_ISL_2492744	N501Y, E484K, K417N, R346K, L452R	Africa / Angola / Luanda	2020-12-09	Instituto Nacional de Investigação em Saúde	CERI, Centre for Epidemic Response and Innovation, Stellenbosch University and KRISP, KZN Research Innovation and Sequencing Platform, UKZN.	Morais J, Neto Z, Afonso P, Miranda J, David K, Inglês L, Pereira A, Paulo A Carralero RR Paixão JP, Freitas RH, Mufinda M, Lutucuta S, Giandhari J, Pillay S, Naidoo Y, Emmanuel SJ, Tegally H, Wilkinson E, de Oliveira
hCoV-19/Angola/CERI-KRISP-K013451/2020	B.1.351	EPI_ISL_2492731	N501Y, E484K, K417N, R346K, L452R	Africa / Angola / Luanda	2020-12-09	Instituto Nacional de Investigação em Saúde	CERI, Centre for Epidemic Response and Innovation, Stellenbosch University and KRISP, KZN Research Innovation and Sequencing Platform, UKZN.	Morais J, Neto Z, Afonso P, Miranda J, David K, Inglês L, Pereira A, Paulo A Carralero RR Paixão JP, Freitas RH, Mufinda M, Lutucuta S, Giandhari J, Pillay S, Naidoo Y, Emmanuel SJ, Tegally H, Wilkinson E, de Oliveira T
hCoV-19/Angola/CERI-KRISP-K012620/2021	B.1.351	EPI_ISL_2492702	N501Y, E484K,	Africa / Angola / Luanda	2021-02-23	Instituto Nacional de	CERI, Centre for Epidemic Response	Morais J, Neto Z, Afonso P, Miranda J, David K, Inglês L,

			K417N, L452R			Investigação em Saúde	and Innovation, Stellenbosch University and KRISP, KZN Research Innovation and Sequencing Platform, UKZN.	Pereira A, Paulo A Carralero RR Paixão JP, Freitas RH, Mufinda M, Lutucuta S, Giandhari J, Pillay S, Naidoo Y, Emmanuel SJ, Tegally H, Wilkinson E, de Oliveira T
hCoV-19/Angola/KRISP-K009792/2021	A	EPI_ISL_1347942	E484K, R346K, T478R	Africa / Angola / Luanda	2021-02-13	Instituto Nacional de Investigação em Saúde	KRISP, KZN Research Innovation and Sequencing Platform	Morais J, Neto Z, Afonso P, Miranda J, David K, Inglês L, Pereira A, Paulo A Carralero RR Paixão JP, Freitas RH, Mufinda M, Lutucuta S, Giandhari J, Pillay S, Naidoo Y, Emmanuel SJ, Tegally H, Wilkinson E, de Oliveira T
hCoV-19/Turkey/HSGM-FS976/2021	B.1.617.2	EPI_ISL_3403341	N501Y, E484K, L452R, T478K, F490S	Europe / Turkey	2021-08-05	Ministry of Health Turkey	Ministry of Health Turkey	Fatma Bayrakdar, Yasemin Cosgun, Suleyman Yalcin, Gulay Korukluoglu
hCoV-19/Turkey/HSGM-FS476/2021	B.1.617.2	EPI_ISL_3403280	N501Y, E484K, L452R, T478K, F490S	Europe / Turkey	2021-08-05	Ministry of Health Turkey	Ministry of Health Turkey	Fatma Bayrakdar, Yasemin Cosgun, Suleyman Yalcin, Gulay Korukluoglu
hCoV-19/Turkey/HSGM-B18019/2021	B.1.617.2	EPI_ISL_2958474	N501Y, E484K, L452R, T478K, F490S	Europe / Turkey	2021-06-23	Ministry of Health Turkey	Ministry of Health Turkey	Fatma Bayrakdar, Yasemin Cosgun, Suleyman Yalcin, Gulay Korukluoglu
hCoV-19/South Africa/Tygerberg_1173/2021	B.1.399	EPI_ISL_2876377	N501Y, N440K, Q493K	Africa / South Africa / Western Cape Province / Cape Town Metro	2021-06-23	Division of Medical Virology, National Health Laboratory Service (NHLS), Tygerberg Hospital / Stellenbosch University	Division of Medical Virology, National Health Laboratory Service (NHLS), Tygerberg Hospital / Stellenbosch University	Susan Engelbrecht, Tongai Maponga, Bronwyn Kleinhans, Tania Stander, Gert van Zyl, Wolfgang Preiser
hCoV-19/England/PHEC-30D9F6/2021	B.1.1.7	EPI_ISL_2742396	N501Y, L452R, N439K, N440F	Respiratory Virus Unit, Microbiology Services Colindale, Public Health England	2021	Respiratory Virus Unit, Microbiology Services Colindale, Public Health England	COVID-19 Genomics UK (COG-UK) Consortium	PHE Covid Sequencing Team
hCoV-19/Colombia/MAG-INS-VG-482/2021	B.1.621	EPI_ISL_1220045	N501Y, E484K, R346K	South America / Colombia / Magdalena	2021	Universidad de Magdalena	Instituto Nacional de Salud-Dirección de Investigación en Salud Pública	Katherine Laiton-Donato, Carlos Franco-Muñoz, Diego A. Álvarez-Díaz, Hector Alejandro Ruiz-Moreno, Jhonnatan Reales-González, Diego Andrés Prada, Sheryll Corchuelo, Maria T. Herrera-Sepúlveda, Julian Naizaque, Gerardo Santamaría, Magdalena Wiesner, Martha Lucia Ospina Martínez, Marcela Mercado-Reyes

Accession numbers for SARS-CoV-2 variants containing RBD mutations that would affect receptor binding or antibody responses as discussed in the main text and figures. We gratefully acknowledge the listed authors from the originating laboratories responsible for obtaining the specimens and the submitting laboratories where genetic sequence data were generated and shared through the GISAID Initiative, on which this research is based.

Table S2. Substitutions and deletions in SARS-CoV-2 spike protein expression constructs used in this study.

Virus name	GISAID Accession No.	NTD changes	RBD substitutions	S1 substitutions	S2 substitutions
WT D614G	N/A	-	-	D614G	-
WT D614G/N501Y	N/A	-	N501Y	D614G	-
WT D614G/N439K	N/A	-	N439K	D614G	-
WT D614G/Q493K	N/A	-	Q493K	D614G	-
WT D614G/Q493R	N/A	-	Q493R	D614G	-
Alpha	EPI_ISL_754289	H69del, V70del, Y144del	N501Y	A570D, D614G, P681H	T716I, S982A, D1118H
Alpha A372S	EPI_ISL_754289	H69del, V70del, Y144del	A372S, N501Y	A570D, D614G, P681H	T716I, S982A, D1118H
Beta	EPI_ISL_745186	D80A, D215G, L242del, A243del, L244del	K417N, E484K, N501Y	D614G	A701V
Gamma	EPI_ISL_811149	L18F, T20N, P26S, D138Y, R190S	K417T, E484K, N501Y	D614G, H655Y	T1027I, V1176F
Epsilon	EPI_ISL_1825082	S13I, W152C	L452R	D614G	
Lambda	EPI_ISL_2907514	G75V, T76I, R246del, S247del, Y248del, L249del, T250del, P251del, G252del, D253N	L452Q, F490S	D614G	T859N
Iota E484K	EPI_ISL_1823970	L5F, T95I, D253G	E484K	D614G	A701V
Iota S477N	EPI_ISL_1804950	L5F, T95I, D253G	S477N	D614G	A701V
Kappa	EPI_ISL_1595896	T95I	L452R, E484Q	D614G, P681R	
Delta K417N (AY.2)	EPI_ISL_2907577	T19R, V70F, E156G, F157del, R158del, A222V	K417N, L452R, T478K	D614G, P681R	D950N
Delta +3	EPI_ISL_3403341	T19R	L452R, T478K, E484K, F490S, N501Y, T523N	D614G, P681R	D950N
Day 146*	N/A	G142del, V143del, Y144del, Q183H	N440D, T478K, Y489H, Q493K, S494P, N501Y	D614G	A1020S, I870V
Day 152*	N/A	L141del, G142del, V143del, Y144del, Q183H	T478K, E484A, F486I, Y489H, S494P, N501Y	D614G	I870V
RBM-1	N/A	G142del, V143del, Y144del, Q183H	T478K, E484K, Y489H, Q493K, S494P, N501Y	D614G	I870V
RBM-2	N/A	G142del, V143del, Y144del, Q183H	K417N, T478K, E484K, Y489H, Q493K, S494P, N501Y	D614G	I870V
RBM-3	N/A	D80A, D215G, L242del, A243del, L244del	K417N, N439K, L452R, E484K, N501Y	D614G	A701V

Relevant RBD substitutions are listed based on their location in the N-terminal domain (NTD), receptor-binding domain (RBD), S1 (excluding the NTD and RBD), or S2. WT: wild-type; del: deletion. -: no change. N/A: not applicable. Spike protein expression constructs also contained cytoplasmic tail modifications in S2 that are not listed here (see Materials and Methods for additional information).

Table S3. Antibody Fab, human ACE2 ectodomain, and RBD binding kinetics measured by biolayer interferometry.

Ligand	Analyte	k_a (1/Ms)	k_d (1/S)	K_D (M)	K_D (nM)
SARS-CoV-2 WT	C1C-A3 Fab	1.24E+05	8.12E-05	6.56E-10	0.66
SARS-CoV-2 WT	C1C-C6 Fab	6.79E+04	6.18E-04	9.09E-09	9.09
SARS-CoV-2 WT	C1A-A6 Fab	1.55E+05	6.73E-04	4.35E-09	4.35
SARS-CoV-2 day 146*	C1C-A3 Fab	1.36E+05	7.73E-05	5.69E-10	0.57
SARS-CoV-2 day 146*	C1C-C6 Fab	7.08E+04	7.53E-04	1.06E-08	10.6
SARS-CoV-2 day 146*	C1A-A6 Fab	1.71E+05	6.81E-04	3.98E-09	3.98
SARS-CoV-2 day 152*	C1C-A3 Fab	1.42E+05	1.08E-04	7.64E-10	0.76
SARS-CoV-2 day 152*	C1C-C6 Fab	6.67E+04	7.38E-04	1.11E-08	11.1
SARS-CoV-2 day 152*	C1A-A6 Fab	1.38E+05	4.70E-04	3.40E-09	3.40
SARS-CoV	C1C-A3 Fab	7.16E+05	1.23E-04	1.72E-09	1.72
SARS-CoV	C1C-C6 Fab	7.67E+04	1.17E-02	1.52E-07	152
SARS-CoV	C1A-A6 Fab	1.43E+05	5.57E-04	3.91E-09	3.91
SARS-CoV-2 WT	ACE2	1.67E+05	8.95E-03	5.35E-08	53.5
SARS-CoV-2 WT	ACE2 T55A	1.21E+05	8.03E-03	6.65E-08	66.5
SARS-CoV-2 WT	ACE2 T92A	1.49E+05	4.73E-03	3.18E-08	31.8
SARS-CoV-2 day 146*	ACE2	2.24E+05	1.02E-02	4.55E-08	45.5
SARS-CoV-2 day 146*	ACE2 T55A	2.77E+05	8.90E-03	3.21E-08	32.1
SARS-CoV-2 day 146*	ACE2 T92A	2.88E+05	5.20E-03	1.80E-08	18.0
SARS-CoV-2 day 152*	ACE2	3.15E+04	7.41E-02	2.35E-06	2350
SARS-CoV	ACE2	1.50E+05	3.96E-02	2.64E-07	264

Kinetic parameters of binding were determined for the indicated monomeric proteins using biolayer-interferometry as detailed in the Materials and Methods section. WT: wild-type, signifies Wuhan-Hu-1 sequence.

Table S4. X-ray data collection and refinement statistics

	Day 146* RBD:ACE2[a] (PDB 7SN0)	C1C-A3 Fab[a] (PDB 7SN1)
**Data collection**		
Space group	$P2_12_12_1$	$P12_11$
Cell dimensions		
a, *b*, *c* (Å)	85.7, 104.0, 223.9	62.7, 52.4, 71.4
α, β, γ (°)	90.0, 90.0, 90.0	90.0, 113.7, 90.0
Resolution (Å)	200–3.06 (3.25–3.06)[b]	200–1.47 (1.48–1.47)[b]
R_{sym} or R_{merge}	0.246 (2.29)[b]	0.059 (0.934)[b]
R_{meas}	0.294 (2.75)[b]	0.077 (1.22)[b]
I / σ	5.29 (0.84)[b]	8.45 (1.14)[b]
Completeness (%)	98.5 (19.6)[b]	98.9 (96.2)[b]
Redundancy	3.37 (3.14)[b]	2.19 (2.17)[b]
**Refinement**		
Resolution (Å)	111.96–3.08	55.64–1.47
No. reflections	37515	71663
R_{work} / R_{free}	0.235/0.286	0.177/0.206
No. atoms		
Protein	12822	3301
Ligand/ion	866	0
Water	0	601
B-factors (Å^2)		
Protein	94.5	24.05
Ligand/ion	146.1	-
Water	-	40.81
R.m.s. deviations		
Bond lengths (Å)	0.008	0.008
Bond angles (°)	1.000	1.040

[a] Numbers of crystals for day 146* RBD:ACE2 and C1C-A3 Fab data were one each.
[b] Values in parentheses are for the highest-resolution shell.

Table S5. Properties of cross-reactive monoclonal antibodies isolated from COVID-19 convalescent individual C1.

							PsV neut.		ELISA (OD 450nm)				
									SARS-CoV-2		SARS-CoV		
Antibody	V_H gene	CDR H3 (a.a.)	Identity (%)	V_L gene	CDR L3 (a.a.)	Identity (%)	SARS-CoV-2	SARS-CoV	HXP	RBD	S2P	RBD	Ctrl
C1C-A3	V_H3-33	16	98.96	V_κ3-11	11	97.85	+	-	2.52	2.53	2.35	2.23	0.08
C1C-A12	V_H3-30	16	99.31	V_κ2-29	10	98.98	-	-	2.19	0.02	1.53	0.04	0.03
C1C-C6	V_H3-48	16	98.96	V_κ1-39	9	99.28	+	+	2.14	2.32	2.03	2.27	0.02
C1C-C8	V_H3-30	14	100	V_κ1-13	10	99.64	-	-	2.05	0.03	1.40	0.03	0.03
C1C-C12	V_H4-61	14	94.85	V_κ3-20	10	97.16	-	-	1.75	0.00	0.45	0.03	0.01
C1C-D1	V_H1-18	12	97.57	V_κ1-5	8	97.49	-	-	2.15	0.02	1.70	0.02	0.02
C1C-D4	V_H3-21	21	99.31	V_κ1-39	9	100	-	-	2.25	0.00	1.59	0.01	0.01
C1C-D6	V_H3-23	19	96.18	V_κ1-39	8	98.92	-	-	2.03	0.05	1.70	0.06	0.04
C1C-D8	V_H1-69	15	99.65	V_κ3-11	11	98.92	-	-	2.31	0.06	2.03	0.05	0.04
C1C-D10	V_H3-30-3	14	99.31	V_κ3-20	9	98.94	-	-	2.20	0.00	1.40	0.02	0.02
C1C-D12	V_H3-30-3	14	98.26	V_κ3-20	8	99.65	-	-	2.29	0.00	1.92	0.00	0.01
C1A-A6*	V_H3-11	15	98.96	V_κ1-13	9	98.92	+	+	2.28	2.39	2.24	2.45	0.01

CDR loop lengths are shown as numbers of amino acids (a.a.). ELISA values are colored in shades of blue according to their magnitude; darker shades reflect a stronger signal. HexaPro ("HXP") and "S2P" are constructs representing the prefusion-stabilized form of the spike protein ectodomain for SARS-CoV-2 and SARS-CoV, respectively. RBD: receptor-binding domain; Ctrl: negative control protein Lujo virus GP1. Neut: neutralizing activity as shown in figure S6F. + signifies >90% neutralization at the tested concentration, and - signifies neutralization not meeting this threshold. *C1A-A6 is a previously described SARS-CoV-2 neutralizing antibody isolated from the C1 donor using SARS-CoV-2 S2P as bait (*14*).

Table S6. Cryo-EM data collection, refinement, and validation statistics.

	SARS-CoV-2 S:C1C-A3 Fab (EMDB-25210) (PDB 7SN3)	RBD:C1C-A3 Fab (EMDB-25209) (PDB 7SN2)
**Data collection and processing**		
Magnification (nominal/calibrated)	105,000/60,606	105,000/60,606.06
Voltage (kV)	300	300
Electron exposure (e–/$Å^2$)	50	50
Defocus range (μm)	1.5-2.5	1.5–2.5
Pixel size (Å)	0.825	0.825
Symmetry imposed	*C*1	*C*1
Initial particle images (no.)	1,296,969	218,112
Final particle images (no.)	344,920	106,285
Map resolution (Å)	3.1	4.0
FSC threshold	0.143	0.143
Map resolution range (Å)	∞–3.1	∞–4.0
**Refinement**		
Initial model used (PDB code)	7SN2, 7KN1	7SN1, 7KFV
Model resolution (Å)	3.3	4.4
FSC threshold	0.5	0.5
Model resolution range (Å)	∞–3.3	∞–4.4
Map sharpening *B* factor ($Å^2$)	-103.6	-60.01
Model composition		
Non-hydrogen atoms	27,477	5,170
Protein residues	3,451	650
Ligands	54	10
B factors ($Å^2$)		
Protein	52.1 (1.85–115.8)	111.9 (69.7–179.1)
Ligand	58.0 (13.1–135.2)	124.3 (88.4–155.9)
R.m.s. deviations		
Bond lengths (Å)	0.009	0.007
Bond angles (°)	1.083	1.288
Validation		
MolProbity score[b]	1.27 (99th percentile)	1.43 (97th percentile)
Clashscore	5.15	7.74
Poor rotamers (%)	0.10	0.00
Ramachandran plot		
Favored (%)	99.4	98.9
Allowed (%)	0.36	0.78
Disallowed (%)	0.29	0.31

[a]Refinement statistics were obtained with the program Phenix v1.18.2-3874 (phenix.real_space_refinement) except where otherwise noted.

[b]MolProbity score was obtained from the MolProbity online server (100th percentile is the best among structures within the resolution specified).

Table S7. Antibody RBD binding kinetics for a deglycosylated SARS-CoV RBD measured by surface plasmon resonance.

Ligand	Analyte	k_a (1/Ms)	k_d (1/S)	K_D (M)	K_D (nM)
C1C-A3 IgG	SARS-CoV RBD	-	-	W.B.	W.B.
C1C-A3 IgG	SARS-CoV RBD deglycosylated	9.5E+04	1.2E-03	1.3E-08	13 nM
C1A-A6 IgG	SARS-CoV RBD	1.6E+05	1.5E-03	9.4E-09	9.4 nM
C1A-A6 IgG	SARS-CoV RBD deglycosylated	2.3E+05	1.3E-03	5.4E-09	5.4 nM

Kinetic parameters of binding were determined for the indicated antibodies and unmodified or enzymatically deglycosylated SARS-CoV RBD using surface plasmon resonance as detailed in the Materials and Methods section. W.B.: weak binding.

Table S8. Human-derived SARS-CoV-2 sequences containing the A372S/T$_{RBD}$ substitution.

Virus name	PANGO lineage	Accession No.	RBD mutations of interest	Location	Collection date	Originating laboratory	Submitting laboratory	Authors
hCoV-19/USA/WA-CDC-QDX28381256/2021	B.1.617.2	EPI_ISL_4466927	A372S, L452R, T478K	North America / USA / Washington	2021-08-28	Quest Diagnostics Incorporated	Centers for Disease Control and Prevention Division of Viral Diseases, Pathogen Discovery	Dakota Howard, Dhwani Batra, Peter Cook, Jason Caravas, Benjamin Rambo-Martin, Scott Sammons, Yvette Unoarumhi, Matthew Schmerer, Kristine Lacek, Tymeckia Kendall, Victoria Caban Figueroa, Shatavia Morrison, Christopher Gulvick, Erisa Sula ,S. Rosenthal, A. Gerasimova, R. Kagan, B. Anderson, M. Hua, Y. Liu, L. Bernstein, K. Livingston, A. Perez, I. Shlyakhter, R. Rolando, R. Owen, P. Tanpaiboon, F. Lacbawan, Clinton Paden, Duncan MacCannell
hCoV-19/USA/MD-HP09204-PIDJWRTCUN/2020	B.1.369	EPI_ISL_4418832	A372S	North America / USA / Maryland	2020-05-07	Johns Hopkins Hospital Department of Pathology	Johns Hopkins Hospital Department of Pathology	C. Paul Morris, David Gaston, Julie M. Norton, Matthew Schwartz, Heba H. Mostafa
hCoV-19/USA/WA-CDC-QDX27909420/2021	B.1.617.2	EPI_ISL_4021446	A372S, L452R, T478K	North America / USA / Washington	2021-08-16	Quest Diagnostics Incorporated	Centers for Disease Control and Prevention Division of Viral Diseases, Pathogen Discovery	Dakota Howard, Dhwani Batra, Peter Cook, Kara Moser, Adrian Paskey, Jason Caravas, Benjamin Rambo-Martin, Shatavia Morrison, Christopher Gulvick, Sammons, Scott ,Yvette Unoarumhi, Darlene Wagner, Matthew Schmerer, S. Rosenthal, A. Gerasimova, R. Kagan, B. Anderson, M. Hua, Y. Liu, L. Bernstein, K. Livingston, A. Perez, I. Shlyakhter, R. Rolando, R. Owen, P. Tanpaiboon, F. Lacbawan, Clinton Paden, Duncan MacCannell
hCoV-19/USA/WA-CDC-QDX27909376/2021	B.1.617.2	EPI_ISL_4019269	A372S, L452R, T478K	North America / USA / Washington	2021-08-14	Quest Diagnostics Incorporated	Centers for Disease Control and Prevention Division of Viral Diseases, Pathogen Discovery	Dakota Howard, Dhwani Batra, Peter Cook, Kara Moser, Adrian Paskey, Jason Caravas, Benjamin Rambo-Martin, Shatavia Morrison, Christopher Gulvick, Sammons, Scott ,Yvette Unoarumhi, Darlene Wagner, Matthew Schmerer, S. Rosenthal, A. Gerasimova, R. Kagan, B. Anderson, M. Hua, Y. Liu, L. Bernstein, K. Livingston, A. Perez, I. Shlyakhter, R. Rolando, R. Owen, P. Tanpaiboon, F. Lacbawan, Clinton Paden, Duncan MacCannell
hCoV-19/USA/OR-CDC-UW21081395459/2021	AY.4	EPI_ISL_3759504	A372S, L452R, T478K	North America / USA / Oregon	2021-08-13	UW Virology Lab	UW Virology Lab	Pavitra Roychoudhury, Shah Mohamed Bakhash, Ricardo Perez, Maria Lukes, Hong Xie, Tien V. Nguyen, Savanna S. Carmack, Sean Ellis, Nathan Breit, Robert J. Livingston, Meei-Li Huang, Keith R Jerome, Patrick Mathias, Alexander Greninger
hCoV-19/USA/WA-CDC-QDX27713230/2021	AY.4	EPI_ISL_3740150	A372S, L452R, T478K	North America / USA / Washington	2021-08-11	Quest Diagnostics Incorporated	Centers for Disease Control and Prevention Division of Viral Diseases, Pathogen Discovery	Dakota Howard,Dhwani Batra,Peter Cook,Kara Moser,Adrian Paskey,Jason Caravas,Benjamin Rambo-Martin,Shatavia Morrison,Christopher Gulvick, Scott Sammons,Yvette Unoarumhi,Darlene Wagner,Matthew Schmerer,S. Rosenthal,A. Gerasimova,R. Kagan,B.

								Anderson,M. Hua,Y. Liu,L. Bernstein,K. Livingston,A. Perez,I. Shlyakhter,R. Rolando,R. Owen,P. Tanpaiboon,F. Lacbawan,Clinton Paden,Duncan MacCannell
hCoV-19/England/PHEC-N30AN768/2021	AY.4	EPI_ISL_3080914	A372S, L452R, T478K	Europe / United Kingdom / England	2021-07-14	Respiratory Virus Unit, Microbiology Services Colindale, Public Health England	COVID-19 Genomics UK (COG-UK) Consortium	PHE Covid Sequencing Team
hCoV-19/England/LOND-124FE60/2020	B.1.1.164	EPI_ISL_665190	A372S	Europe / United Kingdom / England	2020-04-09	University College London Hospital	COVID-19 Genomics UK (COG-UK) Consortium	Judith Heaney, Matthew Byott, Catherine Houlihan, Dan Frampton, Stuart Kirk, Moira Spyer and Eleni Nastouli
hCoV-19/Wales/PHWC-26365/2020	B.3.1	EPI_ISL_422309	A372S	Europe / United Kingdom / Wales	2020-03-26	Wales Specialist Virology Centre	Public Health Wales Microbiology Cardiff	Catherine Moore, Johnathan Evans, Malorie Perry, Simon Cottrell, Alec Birchley, Alexander Adams, Amy Gaskin, Bree Gatica-Wilcox, Jason Coombes, Lauren Gilbert, Lee Graham, Nicole Pacchiarini, Sara Kumziene-Summerhayes, Sarah Taylor, Sophie Jones, Sara Rey, Matthew Bull, Joanne Watkins, Sally Corden, Tom Connor
hCoV-19/Germany/NW-HHU-10343/2021	AY.4	EPI_ISL_4458254	A372T, L452R, T478K	Europe / Germany / North Rhine-Westphalia	2021-09-12	ZOTZ KLIMAS MVZ Düsseldorf-Centrum GbR ÜBAG für Labormedizin, Genetik, Zytologie, Pathologie	Center of Medical Microbiology, Virology, and Hospital Hygiene, University of Duesseldorf	Maximilian Damagnez; Alexander Dilthey; Patrick Finzer; Katrin Hoffmann; Torsten Houwaart; Lisanna Hülse; Malte Kohns Vasconcelos; Nadine Lübke; Jessica Nicolai; Klaus Pfeffer; Daniel Strelow; Jörg Timm; Andreas Walker; Tobias Wienemann; Rainer Zotz
hCoV-19/Germany/NW-HHU-10863/2021	AY.4	EPI_ISL_4447517	**A372T,** L452R, T478K	Europe / Germany / North Rhine-Westphalia	2021-09-06	ZOTZ KLIMAS MVZ Düsseldorf-Centrum GbR ÜBAG für Labormedizin, Genetik, Zytologie, Pathologie	Center of Medical Microbiology, Virology, and Hospital Hygiene, University of Duesseldorf	Maximilian Damagnez; Alexander Dilthey; Patrick Finzer; Katrin Hoffmann; Torsten Houwaart; Lisanna Hülse; Malte Kohns Vasconcelos; Nadine Lübke; Jessica Nicolai; Klaus Pfeffer; Daniel Strelow; Jörg Timm; Andreas Walker; Tobias Wienemann; Rainer Zotz
hCoV-19/Germany/NW-RKI-I-249329/2021	AY.4	EPI_ISL_4446061	A372T, L452R, T478K, K356E	Europe / Germany / North Rhine-Westphalia	2021-09-14	SYNLAB MVZ Leverkusen	Robert Koch Institute	Oliver Drechsel
hCoV-19/Germany/NW-RKI-I-248155/2021	AY.4	EPI_ISL_4444859	A372T, L452R, T478K, K356E	Europe / Germany / North Rhine-Westphalia	2021-09-11	Labor Dr. Wisplinghoff - Köln	Robert Koch Institute	Oliver Drechsel
hCoV-19/Germany/NW-RKI-I-248019/2021	AY.4	EPI_ISL_4444632	A372T, L452R, T478K, K356E	Europe / Germany / North Rhine-Westphalia	2021-09-11	Labor Dr. Wisplinghoff - Köln	Robert Koch Institute	Oliver Drechsel
hCoV-19/Germany/NW-RKI-I-244679/2021	AY.4	EPI_ISL_4441712	A372T, L452R, T478K	Europe / Germany / North Rhine-Westphalia	2021-09-08	Limbach - MVZ Labor Eveld & Kollegen Essen	Robert Koch Institute	Oliver Drechsel
hCoV-19/USA/MD-HP09014-PIDYQFYQJJ/2020	hCoV-19/USA/MD-HP09014-	EPI_ISL_4418770	A372T	North America / USA / Maryland	2020-08-26	Johns Hopkins Hospital Department of Pathology	Johns Hopkins Hospital Departmen	C. Paul Morris, David Gaston, Julie M. Norton, Matthew Schwartz, Heba H. Mostafa

	PIDYQFY QJJ/2020						t of Pathology	
hCoV-19/USA/TN-SPHL-0203/2021	B.1.2	EPI_ISL_4261403	A372T, E484Q	North America / USA / Tennessee	2021-03-25	TN DOH Lab Services	TN DOH Lab Services	Karen Beasley-Maynard, Brian Selinsky
hCoV-19/England/BRBR-1E1A3DC/2021	AY.4	EPI_ISL_4248074	A372T, L452R, T478K	Europe / United Kingdom / England	2021-09-09	Berkshire and Surrey Pathology Services Lighthouse Laboratory	Wellcome Sanger Institute for the COVID-19 Genomics UK (COG-UK) Consortium	Berkshire and Surrey Pathology Services Lighthouse Laboratory and Alex Alderton, Roberto Amato, Jeffrey Barrett, Sonia Goncalves, Ewan Harrison, David K. Jackson, Ian Johnston, Dominic Kwiatkowski, Cordelia Langford, John Sillitoe on behalf of the Wellcome Sanger Institute COVID-19 Surveillance Team
hCoV-19/Germany/NW-HHU-10078/2021	AY.4	EPI_ISL_4089467	A372T, L452R, T478K	Europe / Germany / North Rhine-Westphalia	2021-08-30	ZOTZ KLIMAS MVZ Düsseldorf-Centrum GbR ÜBAG für Labormedizin, Genetik, Zytologie, Pathologie	Center of Medical Microbiology, Virology, and Hospital Hygiene, University of Duesseldorf	Maximilian Damagnez, Alexander Dilthey, Patrick Finzer;Katrin Hoffmann, Torsten Houwaart, Lisanna Hülse, Malte Kohns Vasconcelos, Nadine Lübke, Jessica Nicolai, Klaus Pfeffer, Daniel Strelow, Jörg Timm, Andreas Walker, Tobias Wienemann, Rainer Zotz
hCoV-19/USA/CA-SEARCH-47335/2021	B.1.561	EPI_ISL_3937027	A372T, E484K	North America / USA / California / San Diego	2021-08-11	EXCITE Lab	Andersen lab at Scripps Research	David Pride, Sharon Reed, Chip Schooley, Angela Scioscia, Natasha Martin Cheryl Anderson, Sawyer Farmer, Abigail Schnapper, Helena Tubb, Tommy Valles + SEARCH
hCoV-19/USA/CA-CDC-FG-083865/2021	B.1.617.2	EPI_ISL_3753602	A372T, L452R, T478K	North America / USA / California	2021-08-16	Fulgent Genetics	Centers for Disease Control and Prevention Division of Viral Diseases, Pathogen Discovery	Dakota Howard, Dhwani Batra, Peter Cook, Kara Moser, Adrian Paskey, Jason Caravas, Benjamin Rambo-Martin, Shatavia Morrison, Christopher Gulvick, Scott Sammons, Yvette Unoarumhi, Darlene Wagner, Matthew Schmerer, Harry Gao, Mickey Li, John Gao, Joseph Fierro,Benafsh Sapra, Becky Tsai, Yan Meng, Doreen Ng, James Xie, Clinton Paden, Duncan MacCannell
hCoV-19/Japan/YCH0580/2021	AY.29	EPI_ISL_3706157	A372T, F392S, L452R, T487K	Asia / Japan / Yamanashi	2021-08-14	Department of Microbiology, Yamanashi Institute for Public Health and Environment	Genome Analysis Center, Yamanashi Central Hospital	Yosuke Hirotsu
hCoV-19/USA/CA-ALSR-43701/2021	B.1.561	EPI_ISL_3509013	A372T, E484K, S477N	North America / USA / California / San Diego	2021-07-02	EXCITE Lab	Andersen lab at Scripps Research	David Pride, Sharon Reed, Chip Schooley, Angela Scioscia, Natasha Martin Cheryl Anderson, Sawyer Farmer, Abigail Schnapper, Helena Tubb, Tommy Valles + SEARCH
hCoV-19/Germany/RP-RKI-I-198680/2021	B.1.258	EPI_ISL_3497667	A372T, N439K, G476S	Europe / Germany / Rhineland-Palatinate	2021-08-07	SYNLAB MVZ Trier	Robert Koch Institute	Oliver Drechsel
hCoV-19/France/NAQ-Poitiers_2107_170859/2021	B.1.1.318	EPI_ISL_3246237	A372T, E484K	Europe / France / Nouvelle-Aquitaine / La Rochelle	2021-07-17	CH La Rochelle	CHU Poitiers	Caroline Michaud, Maxime Pichon, Manon Prat, Valentin Bon-Baret, Birama N'Diaye, Magali Garcia, Agnes Beby-Defaux, Nicolas Leveque
hCoV-19/South Korea/KDCA5804/2021	B.1.619	EPI_ISL_2967517	A372T, E484K, N440K	hCoV-19/South Korea/KDCA5804/2021	2021-07-01	Division of Emerging Infectious Diseases, Bureau of Infectious Diseases Diagnosis Control, Korea	Division of Emerging Infectious Diseases, Bureau of Infectious Diseases Diagnosis Control, Korea	Jeong-Ah Kim, Ae Kyung Park, Il-Hwan Kim, Heui Man Kim, Jeong-Min Kim, Chae Young Lee, Eun-Jin Kim

						Disease Control and Prevention Agency	Disease Control and Prevention Agency	
hCoV-19/Poland/PZH-NIZP-0256/2021	B.1.258	EPI_ISL_2810326	A372T, R357I, N501Y, N439K	Europe / Poland / Opolskie / Opole	2021-06-14	ZAKŁAD DIAGNOSTYKI LABORATORYJNEJ; SZPITAL WOJEWÓDZKI W OPOLU SP. Z O.O.	National Institute of Public Health - National Institute of Hygiene	Wołkowicz Tomasz, Sadkowska-Todys Małgorzata, Gierczyński Rafał
hCoV-19/USA/MN-UMGC-2071/2021	B.1.1.7	EPI_ISL_2699059	A372T N501Y,	North America / USA / Minnesota	2021-04-26	Public Health Laboratory, Minnesota Department of Health	University of Minnesota Genomics Center	Daryl M. Gohl, John Garbe, Jaquelyn Kuriger-Laber, Corbin Dirkx, and Sean Wang
hCoV-19/Croatia/2842/2021	B.1.258	EPI_ISL_2626505	A372T, N439K, S494P	Europe / Croatia	2021-03-19	Institute of Public Health of Zagreb County	Croatian Institute of Public Health	Irena Tabain, Ivana Ferenčak
hCoV-19/Netherlands/ZH-RIVM-35696/2021	B.1.1.7	EPI_ISL_2406154	A372T, N501Y	Europe / Netherlands / Zuid-Holland	2021-05-15	Dutch COVID-19 response team	National Institute for Public Health and the Environment (RIVM)	Adam Meijer, Harry Vennema, Dirk Eggink, Jeroen Cremer, Sharon van den Brink, Bas van der Veer, AnneMarie van den Brandt, Lisa Wijsman, Kim Freriks, Ryanne Jaarsma, Euníce Then, Lynn Aarts, Sanne Bos, Melissa van Tuil, Linda van de Nes, Sjoerd Kuiling, James Groot, Florian Zwagemaker, Dennis Schmitz, Annelies Kroneman, Karim Hajji, Chantal Reusken, on behalf of the national COVID-19 response team
hCoV-19/Poland/WGS-CoV_PZH-GUM-1303/2021	B.1.1.7	EPI_ISL_2377877	A372T, N501Y, S371Y	Europe / Poland / Pomorskie	2021-05-13	WSSE w Gdańsku	Tricity SARS-CoV-2 sequencing consortium: University of Gdansk, Medical University of Gdansk, Vaxican Ltd., Invicta Ltd. 2. National Institute of Public Health - National Institute of Hygiene, Warsaw, Poland	Maciej Kosinski, Celina Cybulska, Krystyna Bienkowska Szewczyk, Maciej Grzybek, Karolina Gackowska, Marcin Lubocki, Katarzyna Groth, Lukasz Rabalski, Katarzyna Zacharczuk, Magdalena Nowakowska, Małgorzata Sadkowska-Todys, Tomasz Wołkowicz
hCoV-19/Spain/VC-IBV-99028544/2021	B.1.1.7	EPI_ISL_2271457	A372T, N501Y	Europe / Spain / Comunitat Valenciana / Alicante	2021-03-01	Hospital General Universitario de Alicante - Instituto de Investigación Sanitaria y Biomédica de Alicante	SeqCOVID-SPAIN consortium /IBV(CSIC)	Maripaz Ventero Martín, Carmen Molina Pardines and SeqCOVID-SPAIN consortium
hCoV-19/USA/TX-HMH-MCoV-40150/2020	B.1	EPI_ISL_2223312	A372T	North America / USA / Texas / Houston	2020-08-04	Houston Methodist Hospital	Houston Methodist Hospital	Randall J. Olsen, Paul A. Christensen, S. Wesley Long, Sishir Subedi, Robert Olson, Marcus Nguyen, James J. Davis, Matthew Ojeda Saavedra, Prasanti Yerramilli, Layne Pruitt, Kristina Reppond, Madison N. Shyer, Jessica Cambric, Ryan Gadd, Ilya J. Finkelstein, Jimmy Gollihar, and James M. Musser
	B.1.258	EPI_ISL_2165898	A372T,	North America /	2021-03-01		Public Health	Buss, E, Croxen M, Deo A, Dieu P, Gill K, Ferrato C,

hCoV-19/Canada/ABPHL-07115/2021			E484Q, N439K	Canada / Alberta		Alberta Precision Labs (APL)	Agency of Canada (PHAC) National Microbiology Laboratory	Khan F, Koleva P, Li V, Lloyd C, Lynch T, Ma R, Murphy S, Pabbaraju K, Shokoples S, Tipples G, Thayer J, Whitehouse M, Wong A, Yu C, Zelyas N
hCoV-19/Canada/ABPHL-07076/2021	B.1.258	EPI_ISL_2165875	A372T, E484Q, N439K	North America / Canada / Alberta	2021-02-27	Alberta Precision Labs (APL)	Public Health Agency of Canada (PHAC) National Microbiology Laboratory	Buss, E, Croxen M, Deo A, Dieu P, Gill K, Ferrato C, Khan F, Koleva P, Li V, Lloyd C, Lynch T, Ma R, Murphy S, Pabbaraju K, Shokoples S, Tipples G, Thayer J, Whitehouse M, Wong A, Yu C, Zelyas N
hCoV-19/Canada/ABPHL-06984/2021	B.1.258	EPI_ISL_2165799	A372T, E484Q, N439K	North America / Canada / Alberta	2021-03-01	Alberta Precision Labs (APL)	Public Health Agency of Canada (PHAC) National Microbiology Laboratory	Buss, E, Croxen M, Deo A, Dieu P, Gill K, Ferrato C, Khan F, Koleva P, Li V, Lloyd C, Lynch T, Ma R, Murphy S, Pabbaraju K, Shokoples S, Tipples G, Thayer J, Whitehouse M, Wong A, Yu C, Zelyas N
hCoV-19/Belgium/Aalst-OLVZ-8094777/2021	B.1.1.7	EPI_ISL_2017495	A372T, N501Y, S371Y	Europe / Belgium	2021-05-03	OLVZ Aalst	OLVZ Aalst	OLVZ Aalst
hCoV-19/Germany/ST-MD2171/2020	B.1.258	EPI_ISL_1670378	A372T, N439K,	Europe / Germany / Saxony-Anhalt	2021-04-01	Institute of Medical Microbiology and Hospital Hygiene	Institute of Medical Microbiology and Hospital Hygiene	Achim Kaasch, Aljoscha Tersteegen
hCoV-19/USA/OH-PLMI-HNYM7-00176/2021	B.1.2	EPI_ISL_1575358	A372T	North America / USA / Ohio	2021-03	Pathology and Laboratory Medicine Institute, Cleveland Clinic, Ohio, USA	Pathology and Laboratory Medicine Institute, Cleveland Clinic, Ohio, US	Joy Nakitandwe, Zheng Jin Tu, Jay Brock, Yu-Wei Cheng, Gary Procop, Daniel Rhoads, Daniel H. Farkas, David Bosler
hCoV-19/England/ALDP-149AABB/2021	B.1.1.7	EPI_ISL_1484875	A372T, N501Y	Europe / United Kingdom / England	2021-03-28	Lighthouse Lab in Alderley Park	Wellcome Sanger Institute for the COVID-19 Genomics UK (COG-UK) Consortium	Jacquelyn Wynn, Mairead Hyland, The Lighthouse Lab in Alderley Park and Alex Alderton, Roberto Amato, Jeffrey Barrett, Sonia Goncalves, Ewan Harrison, David K. Jackson, Ian Johnston, Dominic Kwiatkowski, Cordelia Langford, John Sillitoe on behalf of the Wellcome Sanger Institute COVID-19 Surveillance Team
hCoV-19/Germany/BB-RKI-I-028191/2021	B.1.177.86	EPI_ISL_1211222	A372T	Europe / Germany / Brandenburg	2021-02-17	Gesundheitszentrum Brandenburg an der Havel GmbH bzw. Institut für Laboratoriumsmedizin Brandenburg an der Havel	Robert Koch Institute	Oliver Drechsel
hCoV-19/Austria/CeMM4058/2021	B.1.1.7	EPI_ISL_1117857	A372T, N501Y	Europe / Austria / Vienna	2021-02-12	Austrian Agency for Health and Food Safety (AGES)	Austrian Agency for Health and Food Safety (AGES)	Lukas Endler, Anna Schedl, Thomas Penz, Benedikt Agerer, Maelle Le Moing, Michael Schuster, Bekir Erguner, Jan Laine, Martin Senekowitsch, Christoph Bock, Andreas Bergthaler
hCoV-19/Austria/CeMM4055/2021	B.1.1.7	EPI_ISL_1117855	A372T, N501Y	Europe / Austria / Vienna	2021-02-12	Austrian Agency for Health and Food Safety (AGES)	Bergthaler laboratory, CeMM Research Center for Molecular Medicine of the Austrian Academy of Sciences	Lukas Endler, Anna Schedl, Thomas Penz, Benedikt Agerer, Maelle Le Moing, Michael Schuster, Bekir Erguner, Jan Laine, Martin Senekowitsch, Christoph Bock, Andreas Bergthale
hCoV-19/England/LIVE-DF5B88/2021	B.1.1.7	EPI_ISL_997429	A372T, N501Y	Europe / United Kingdom / England	2021-01-28	Liverpool Clinical Laboratories	COVID-19 Genomics UK (COG-UK)	Sam Haldenby, Anita Lucaci, Steve Paterson, Julian Hiscox, Alistair Darby, M Almsaud, A Alrezaihi, Muhannad Alruwaili, Stuart D

							Consortium	Armstrong, Jones Benjamin, Eleanor G Bentley, Anu Chawla, Jordan J Clark, Angela Cowell, Richard Eccles, Isabel García-Dorival, Matthew Gemmell, Alessandro Gerada, PKF Gilmore, Richard Gregory, Ximeng Han, Catherine Hartley, Margaret Hughes, Miren Iturriza-Gomara, James Johnson, L Luu, Jenifer Manson, Charlotte Nelson, Elaine O'Toole, Cassie Olateju, Rebekah Penrice-Randal , Lucille Rainbow, N.P Randle, Trevor Ian Robinson, Parul Sharma, Ghada T Shawli, James P Stewart, Neil Swainston, Ecaterina Vamos, Joanne Watts, Mark Whitehead
hCoV-19/Denmark/DCGC-23798/2020	B.1	EPI_ISL_997429	A372T, S477N	Europe / Denmark / Sjaelland	2020-12-21	Department of Virus and Microbiological Special Diagnostics, Statens Serum Institut, Copenhagen, Denmar	Albertsen Lab, Department of Chemistry and Bioscience, Aalborg University, Denmark	Danish Covid-19 Genome Consortium
hCoV-19/England/QEUH-B2E432/2020	B.1.177.4	EPI_ISL_646330	A372T	Europe / United Kingdom / England	2020-11-03	Lighthouse Lab in Glasgow	Wellcome Sanger Institute for the COVID-19 Genomics UK (COG-UK) Consortium	Harper VanSteenhouse, Yumi Kasai, David Gray, Carol Clugston, Anna Dominiczak and Alex Alderton, Roberto Amato, Sonia Goncalves, Ewan Harrison, David K. Jackson, Ian Johnston, Dominic Kwiatkowski, Cordelia Langford, John Sillitoe on behalf of the Wellcome Sanger Institute COVID-19 Surveillance Team
hCoV-19/England/CAMB-1AB1A9/2020	B.40	EPI_ISL_470305	A372T	Europe / United Kingdom / England	2020-04-19	Department of Pathology, University of Cambridge	Wellcome Sanger Institute for the COVID-19 Genomics UK (COG-UK) consortium	Luke W Meredith, M. Estée Török , Myra Hosmillo, William L. Hamilton, Martin D. Curran, Theresa Feltwell, Grant Hall, Anna Yakovleva, Fahad A Khokhar, Charlotte J. Houldcroft, Laura G Caller, Aminu S. Jahun, Sarah L. Caddy, Ian Goodfellow; and Alex Alderton, Roberto Amato, Sonia Goncalves, Ewan Harrison, David K. Jackson, Ian Johnston, Dominic Kwiatkowski, Cordelia Langford, John Sillitoe on behalf of the Wellcome Sanger Institute COVID-19 Surveillance Team (http://www.sanger.ac.uk/covid-team)
hCoV-19/England/CAMB-770E6/2020	B.40	EPI_ISL_439380	A372T	Europe / United Kingdom / England	2020-03-31	Department of Pathology, University of Cambridge	Wellcome Sanger Institute for the COVID-19 Genomics UK (COG-UK) consortium	Luke W Meredith, M. Estée Török, Myra Hosmillo, William L. Hamilton, Martin D. Curran, Theresa Feltwell, Grant Hall, Anna Yakovleva, Fahad A Khokhar, Charlotte J. Houldcroft, Laura G Caller, Aminu S. Jahun, Sarah L. Caddy, Ian Goodfellow, Alex Alderton, Roberto Amato, Sonia Goncalves, Ewan Harrison, David K. Jackson, Ian Johnston, Dominic Kwiatkowski, Cordelia Langford, John Sillitoe on behalf of the Wellcome Sanger Institute COVID-19 Surveillance Team (http://www.sanger.ac.uk/covid-team)

List of GISAID accession numbers for SARS-CoV-2 variants containing RBD mutations that would introduce an N-linked glycan on $N370_{RBD}$ by creating a sequon (N-X-S/T, in which X is any amino acid other than proline) (*89*). We gratefully acknowledge the listed authors from the

originating laboratories responsible for obtaining the specimens and the submitting laboratories where genetic sequence data were generated and shared through the GISAID Initiative, on which this research is based.

Table S9. Human-derived SARS-CoV-2 sequences containing the D364N$_{RBD}$ substitution.

Virus name	Pango lineage	Accession No.	RBD mutations of interest	Location	Collection date	Originating laboratory	Submitting laboratory	Authors
hCoV-19/Chile/RM-150319/2021	B.1.621	EPI_ISL_4659689	D364N, N501Y, E484K, R346K, S371F	South America / Chile / Region Metropolitana de Santiago / Penalolen	2021-09-07	Genetica Molecular and Subdepartamento de Virologia ISP Chile	Instituto de Salud Publica de Chile	Karen Orostica, Constanza Campano, Barbara Parra, Loredana Arata, Gisselle Barra, Patricia Bustos, Rodrigo Fasce, Javier Tognarelli, Andres Castillo, Soledad Ulloa, Jorge Fernandez
hCoV-19/Chile/BI-152645/2021	B.1.621	EPI_ISL_4658473	D364N, N501Y, R346K, S371F	South America / Chile / Biobio / Concepcion	2021-09-13	Genetica Molecular and Subdepartamento de Virologia ISP Chile	Instituto de Salud Publica de Chile	Karen Orostica, Constanza Campano, Barbara Parra, Loredana Arata, Gisselle Barra, Patricia Bustos, Rodrigo Fasce, Javier Tognarelli, Andres Castillo, Soledad Ulloa, Jorge Fernandez
hCoV-19/Chile/BI-151300/2021	B.1.621	EPI_ISL_4636536	D364N, N501Y, R346K, S371F	South America / Chile / Biobio / Santa Juana	2021-09-11	Genetica Molecular and Subdepartamento de Virologia ISP Chile	Instituto de Salud Publica de Chile	Karen Orostica, Constanza Campano, Barbara Parra, Loredana Arata, Gisselle Barra, Patricia Bustos, Rodrigo Fasce, Javier Tognarelli, Andres Castillo, Soledad Ulloa, Jorge Fernandez
hCoV-19/USA/WA-UW-21080782200/2021	AY.3	EPI_ISL_4422062	D364N, L452R, T478K	North America / USA / Washington	2021-08-07	UW Virology Lab	UW Virology Lab	Pavitra Roychoudhury, Shah Mohamed Bakhash, Ricardo Perez, Maria Lukes, Hong Xie, Tien V. Nguyen, Savanna S. Carmack, Sean Ellis, Meei-Li Huang, Keith R Jerome, Alexander Greninger
hCoV-19/USA/IL-CDC-ASC210108498/2021	B.1.628	EPI_ISL_4362873	D364N, N440K	North America / USA / Illinois	2021-06-09	Aegis Sciences Corporation	Centers for Disease Control and Prevention Division of Viral Diseases, Pathogen Discovery	Dakota Howard,Dhwani Batra,Peter Cook,Jason Caravas,Benjamin Rambo-Martin,Scott Sammons,Yvette Unoarumhi,Matthew Schmerer,Kristine Lacek,Tymeckia Kendall,Victoria Caban Figueroa,Shatavia Morrison,Christopher Gulvick,Erisa Sula,Cyndi Clark,Patrick Campbell,Rob Case,Vikramsinha Ghorpade,Holly Houdeshell,Ola Kvalvaag,Dillon Nall,Ethan Sanders,Alec Vest,Shaun Westlund,Matthew Hardison,Clinton Paden,Duncan MacCannell

hCoV-19/USA/IL-CDC-ASC210066106/2021	B.1.628	EPI_ISL_4360922	D364N, N440K	North America / USA / Illinois	2021-05-22	Aegis Sciences Corporation	Centers for Disease Control and Prevention Division of Viral Diseases, Pathogen Discovery	Dakota Howard,Dhwani Batra,Peter Cook,Jason Caravas,Benjamin Rambo-Martin,Scott Sammons,Yvette Unoarumhi,Matthew Schmerer,Kristine Lacek,Tymeckia Kendall,Victoria Caban Figueroa,Shatavia Morrison,Christopher Gulvick,Erisa Sula,Cyndi Clark,Patrick Campbell,Rob Case,Vikramsinha Ghorpade,Holly Houdeshell,Ola Kvalvaag,Dillon Nall,Ethan Sanders,Alec Vest,Shaun Westlund,Matthew Hardison,Clinton Paden,Duncan MacCannell
hCoV-19/Chile/LI-138894/2021	B.1.621	EPI_ISL_3988148	D364N, N501Y, E484K, R346K, S371F	South America / Chile / O'Higgins / Pichilemu	2021-08-13	Genetica Molecular and Subdepartamento de Virologia ISP Chile	Instituto de Salud Publica de Chile	Karen Orostica, Constanza Campano, Barbara Parra, Loredana Arata, Gisselle Barra, Patricia Bustos, Rodrigo Fasce, Javier Tognarelli, Andres Castillo, Soledad Ulloa, Jorge Fernandez
hCoV-19/USA/IL-S21WGS3984/2021	B.1.617.2	EPI_ISL_3941559	D364N, L452R, T478K	North America / USA / Illinois / La Salle	2021-08-09	Illinois Department of Public Health - Springfield Lab	Illinois Department of Public Health - Springfield Lab	Bryan Sim, Gordon McCall
hCoV-19/USA/LA-CDC-ASC210066509/2021	B.1.526	EPI_ISL_2489399	D364N	North America / USA / Louisiana	2021-05-24	Aegis Sciences Corporation	Centers for Disease Control and Prevention Division of Viral Diseases, Pathogen Discovery	Dakota Howard, Dhwani Batra, Peter W. Cook, Kara Moser, Adrian Paskey, Jason Caravas, Benjamin Rambo-Martin, Shatavia Morrison, Christopher Gulvick, Scott Sammons, Yvette Unoarumhi, Darlene Wagner, Matthew Schmerer, Cyndi Clark, Patrick Campbell, Rob Case, Vikramsinha Ghorpade, Holly Houdeshell, Ola Kvalvaag, Dillon Nall, Ethan Sanders, Alec Vest, Shaun Westlund, Matthew Hardison, Clinton R. Paden, Duncan MacCannell
hCoV-19/Switzerland/ZH-ETHZ-590478/2021	B.1.1.7	EPI_ISL_1913967	D364N, N501Y	Europe / Switzerland / Zurich	2021-04-20	Viollier AG	Department of Biosystems Science and Engineering, ETH Zurich	Chaoran Chen, Sarah Nadeau, Catharine Aquino, Ivan Topolsky, Philipp Jablonski, Lara Fuhrmann, David Dreifuss, Katharina Jahn, Andreia Cabral de Gouvea, Maria Domenica Moccia, Simon Gruter, Timothy Sykes, Lennart Opitz, Griffin White, Laura Neff, Doris

								Popovic, Andrea Patrignani, Jay Tracy, Ralph Schlapbach, Christiane Beckmann, Maurice Redondo, Olivier Kobel, Christoph Noppen, Sophie Seidel, Noemie Santamaria de Souza, Niko Beerenwinkel, Tanja Stadler
hCoV-19/Spain/MD-IBV-98018319/2021	B.1.177.32	EPI_ISL_1423805	D364N	Europe / Spain / Madrid / Madrid	2021-02-04	Servicio de Microbiología. Hospital Ramón y Cajal. (CIBERESP)	SeqCOVID-SPAIN consortium/IBV(CSIC)	Jose Mª González-Alba, L. Olavarrieta, Val Fernández, Juan C Galán and SeqCOVID-SPAIN consortium
hCoV-19/USA/TX-CDC-STM-000027887/2021	B.1.2	EPI_ISL_1298087	D364N	North America / USA / Texas	2021-03-03	Helix/Illumina	Centers for Disease Control and Prevention Division of Viral Diseases, Pathogen Discovery	Peter W. Cook, Dakota Howard, Dhwani Batra, Ben L. Rambo-Martin, Eileen de Feo, Jan Antico, Christine Tran, Matthew Tolentino, Shannon Wickline, Kim Gietzen, Brad Sickler, Jingtao Liu, Eric Allen, Phil Febbo, Summer Galloway, Nicole L. Washington, Simon White, Geraint Levan, Kelly Schiabor Barrett, Elizabeth Cirulli, Alexandre Bolze, Ary Ascencio, Charlotte Rivera-Garcia, Ryan Cho, Jason Nguyen, Sherry Wang, Jimmy Ramirez, Tyler Cassens, Efren Sandoval, Magnus Isaksson, William Lee, David Becker, Marc Laurent, James Lu, Clinton R. Paden, Suxiang Tong, Duncan MacCannell
hCoV-19/USA/TX-CDC-STM-000027815/2021	B.1.2	EPI_ISL_1298083	D364N	North America / USA / Texas	2021-03-03	Helix/Illumina	Centers for Disease Control and Prevention Division of Viral Diseases, Pathogen Discovery	Peter W. Cook, Dakota Howard, Dhwani Batra, Ben L. Rambo-Martin, Eileen de Feo, Jan Antico, Christine Tran, Matthew Tolentino, Shannon Wickline, Kim Gietzen, Brad Sickler, Jingtao Liu, Eric Allen, Phil Febbo, Summer Galloway, Nicole L. Washington, Simon White, Geraint Levan, Kelly Schiabor Barrett, Elizabeth Cirulli, Alexandre Bolze, Ary Ascencio, Charlotte Rivera-Garcia, Ryan Cho, Jason Nguyen, Sherry Wang, Jimmy Ramirez, Tyler Cassens, Efren Sandoval, Magnus Isaksson, William Lee, David Becker, Marc Laurent, James Lu, Clinton R.

								Paden, Suxiang Tong, Duncan MacCannell
hCoV-19/USA/WA-UW-58951/2021	B.1.234	EPI_ISL_1040008	D364N	North America / USA / Washington	2021-02-01	UW Virology Lab	UW Virology Lab	Pavitra Roychoudhury, Hong Xie, Lasata Shrestha, Shah Mohamed Bakhash, Robert Livingston, Michelle Lin, Noah Baker, Meei-Li Huang, Keith R Jerome, Alexander Greninger
hCoV-19/USA/WA-S4464/2021	B.1.234	EPI_ISL_1015807	D364N	North America / USA / Washington / Snohomish County	2021-01-14	Altius Institute for Biomedical Sciences	Seattle Flu Study	Deborah A. Nickerson, Chris D. Frazar, Jover Lee, Benjamin Pelle, Erica Ryke, Matthew Richardson, Amanda Adler, Elisabeth Brandstetter, Peter D. Han, Kairsten Fay, Misja Ilcisin, Kirsten Lacombe, Thomas R. Sibley, Melissa Truong, Caitlin R. Wolf, Ryan Alexander, Daniel Bates, Rebecca Bruders, Stephanie DeBaun, Clem Green, Muhammad Halimun, Jessica Halow, Kneshay Harper, Matt Hartman, Andrew Meuser, Alex Nguyen, Truong Nguyen, Sofia Olsson, Sadie Patraw, Hannah Petersen, Tobias Ragoczy, Joshua Richards, Jacob Rodriguez, John Stamatoyannopoulos, Julia Wald, Olivia Waltner, Michael Boeckh, Janet A. Englund, Michael Famulare, Barry R. Lutz, Mark J. Rieder, Lea M. Starita, Matthew Thompson, Helen Y. Chu, Jay Shendure, Trevor Bedford
hCoV-19/England/QEUH-B9EF78/2020	B.1.177	EPI_ISL_719737	D364N	Europe / United Kingdom / England	2020-11-18	Lighthouse Lab in Glasgow	Wellcome Sanger Institute for the COVID-19 Genomics UK (COG-UK) Consortium	Harper VanSteenhouse, Yumi Kasai, David Gray, Carol Clugston, Anna Dominiczak and Alex Alderton, Roberto Amato, Sonia Goncalves, Ewan Harrison, David K. Jackson, Ian Johnston, Dominic Kwiatkowski, Cordelia Langford, John Sillitoe on behalf of the

								Wellcome Sanger Institute COVID-19 Surveillance Team
hCoV-19/England/QEUH-B7ED56/2020	B.1.177	EPI_ISL_661810	D364N	Europe / United Kingdom / England	2020-11-14	Lighthouse Lab in Glasgow	Wellcome Sanger Institute for the COVID-19 Genomics UK (COG-UK) Consortium	Harper VanSteenhouse, Yumi Kasai, David Gray, Carol Clugston, Anna Dominiczak and Alex Alderton, Roberto Amato, Sonia Goncalves, Ewan Harrison, David K. Jackson, Ian Johnston, Dominic Kwiatkowski, Cordelia Langford, John Sillitoe on behalf of the Wellcome Sanger Institute COVID-19 Surveillance Team
hCoV-19/England/QEUH-990163/2020	B.1.1	EPI_ISL_539923	D364N	hCoV-19/England/QEUH-990163/2020	2020-08-26	Lighthouse Lab in Glasgow	Wellcome Sanger Institute for the COVID-19 Genomics UK (COG-UK) consortium	Harper VanSteenhouse, Yumi Kasai, David Gray, Carol Clugston, Anna Dominiczak and Alex Alderton, Roberto Amato, Sonia Goncalves, Ewan Harrison, David K. Jackson, Ian Johnston, Dominic Kwiatkowski, Cordelia Langford, John Sillitoe on behalf of the Wellcome Sanger Institute COVID-19 Surveillance Team

List of GISAID accession numbers for SARS-CoV-2 variants containing RBD mutations that would introduce an N-linked glycan on RBD position D364 by creating a sequon (N-X-S/T, in which X is any amino acid other than proline) (*89*). We gratefully acknowledge the listed authors from the originating laboratories responsible for obtaining the specimens and the submitting laboratories where genetic sequence data were generated and shared through the GISAID Initiative, on which this research is based.

References and Notes

1. W. T. Harvey, A. M. Carabelli, B. Jackson, R. K. Gupta, E. C. Thomson, E. M. Harrison, C. Ludden, R. Reeve, A. Rambaut, S. J. Peacock, D. L. Robertson; COVID-19 Genomics UK (COG-UK) Consortium, SARS-CoV-2 variants, spike mutations and immune escape. *Nat. Rev. Microbiol.* **19**, 409–424 (2021). doi:10.1038/s41579-021-00573-0 Medline

2. J. Lan, J. Ge, J. Yu, S. Shan, H. Zhou, S. Fan, Q. Zhang, X. Shi, Q. Wang, L. Zhang, X. Wang, Structure of the SARS-CoV-2 spike receptor-binding domain bound to the ACE2 receptor. *Nature* **581**, 215–220 (2020). doi:10.1038/s41586-020-2180-5 Medline

3. J. Shang, G. Ye, K. Shi, Y. Wan, C. Luo, H. Aihara, Q. Geng, A. Auerbach, F. Li, Structural basis of receptor recognition by SARS-CoV-2. *Nature* **581**, 221–224 (2020). doi:10.1038/s41586-020-2179-y Medline

4. L. Piccoli, Y.-J. Park, M. A. Tortorici, N. Czudnochowski, A. C. Walls, M. Beltramello, C. Silacci-Fregni, D. Pinto, L. E. Rosen, J. E. Bowen, O. J. Acton, S. Jaconi, B. Guarino, A. Minola, F. Zatta, N. Sprugasci, J. Bassi, A. Peter, A. De Marco, J. C. Nix, F. Mele, S. Jovic, B. F. Rodriguez, S. V. Gupta, F. Jin, G. Piumatti, G. Lo Presti, A. F. Pellanda, M. Biggiogero, M. Tarkowski, M. S. Pizzuto, E. Cameroni, C. Havenar-Daughton, M. Smithey, D. Hong, V. Lepori, E. Albanese, A. Ceschi, E. Bernasconi, L. Elzi, P. Ferrari, C. Garzoni, A. Riva, G. Snell, F. Sallusto, K. Fink, H. W. Virgin, A. Lanzavecchia, D. Corti, D. Veesler, Mapping neutralizing and immunodominant sites on the SARS-CoV-2 spike receptor-binding domain by structure-guided high-resolution serology. *Cell* **183**, 1024–1042.e21 (2020). doi:10.1016/j.cell.2020.09.037 Medline

5. A. J. Greaney, A. N. Loes, L. E. Gentles, K. H. D. Crawford, T. N. Starr, K. D. Malone, H. Y. Chu, J. D. Bloom, Antibodies elicited by mRNA-1273 vaccination bind more broadly to the receptor binding domain than do those from SARS-CoV-2 infection. *Sci. Transl. Med.* **13**, eabi9915 (2021). doi:10.1126/scitranslmed.abi9915 Medline

6. K. R. McCarthy, L. J. Rennick, S. Nambulli, L. R. Robinson-McCarthy, W. G. Bain, G. Haidar, W. P. Duprex, Recurrent deletions in the SARS-CoV-2 spike glycoprotein drive antibody escape. *Science* **371**, 1139–1142 (2021). doi:10.1126/science.abf6950 Medline

7. M. McCallum, A. De Marco, F. A. Lempp, M. A. Tortorici, D. Pinto, A. C. Walls, M. Beltramello, A. Chen, Z. Liu, F. Zatta, S. Zepeda, J. di Iulio, J. E. Bowen, M. Montiel-Ruiz, J. Zhou, L. E. Rosen, S. Bianchi, B. Guarino, C. S. Fregni, R. Abdelnabi, S. C. Foo, P. W. Rothlauf, L.-M. Bloyet, F. Benigni, E. Cameroni, J. Neyts, A. Riva, G. Snell, A. Telenti, S. P. J. Whelan, H. W. Virgin, D. Corti, M. S. Pizzuto, D. Veesler, N-terminal domain antigenic mapping reveals a site of vulnerability for SARS-CoV-2. *Cell* **184**, 2332–2347.e16 (2021). doi:10.1016/j.cell.2021.03.028 Medline

8. P. Supasa, D. Zhou, W. Dejnirattisai, C. Liu, A. J. Mentzer, H. M. Ginn, Y. Zhao, H. M. E. Duyvesteyn, R. Nutalai, A. Tuekprakhon, B. Wang, G. C. Paesen, J. Slon-Campos, C. López-Camacho, B. Hallis, N. Coombes, K. R. Bewley, S. Charlton, T. S. Walter, E. Barnes, S. J. Dunachie, D. Skelly, S. F. Lumley, N. Baker, I. Shaik, H. E. Humphries, K. Godwin, N. Gent, A. Sienkiewicz, C. Dold, R. Levin, T. Dong, A. J. Pollard, J. C. Knight, P. Klenerman, D. Crook, T. Lambe, E. Clutterbuck, S. Bibi, A. Flaxman, M. Bittaye, S. Belij-Rammerstorfer, S. Gilbert, D. R. Hall, M. A. Williams, N. G. Paterson, W. James, M. W. Carroll, E. E. Fry, J. Mongkolsapaya, J. Ren, D. I. Stuart, G. R.

Screaton, Reduced neutralization of SARS-CoV-2 B.1.1.7 variant by convalescent and vaccine sera. *Cell* **184**, 2201–2211.e7 (2021). doi:10.1016/j.cell.2021.02.033 Medline

9. W. Dejnirattisai, D. Zhou, P. Supasa, C. Liu, A. J. Mentzer, H. M. Ginn, Y. Zhao, H. M. E. Duyvesteyn, A. Tuekprakhon, R. Nutalai, B. Wang, C. López-Camacho, J. Slon-Campos, T. S. Walter, D. Skelly, S. A. Costa Clemens, F. G. Naveca, V. Nascimento, F. Nascimento, C. Fernandes da Costa, P. C. Resende, A. Pauvolid-Correa, M. M. Siqueira, C. Dold, R. Levin, T. Dong, A. J. Pollard, J. C. Knight, D. Crook, T. Lambe, E. Clutterbuck, S. Bibi, A. Flaxman, M. Bittaye, S. Belij-Rammerstorfer, S. C. Gilbert, M. W. Carroll, P. Klenerman, E. Barnes, S. J. Dunachie, N. G. Paterson, M. A. Williams, D. R. Hall, R. J. G. Hulswit, T. A. Bowden, E. E. Fry, J. Mongkolsapaya, J. Ren, D. I. Stuart, G. R. Screaton, Antibody evasion by the P.1 strain of SARS-CoV-2. *Cell* **184**, 2939–2954.e9 (2021). doi:10.1016/j.cell.2021.03.055 Medline
10. D. Planas, T. Bruel, L. Grzelak, F. Guivel-Benhassine, I. Staropoli, F. Porrot, C. Planchais, J. Buchrieser, M. M. Rajah, E. Bishop, M. Albert, F. Donati, M. Prot, S. Behillil, V. Enouf, M. Maquart, M. Smati-Lafarge, E. Varon, F. Schortgen, L. Yahyaoui, M. Gonzalez, J. De Sèze, H. Péré, D. Veyer, A. Sève, E. Simon-Lorière, S. Fafi-Kremer, K. Stefic, H. Mouquet, L. Hocqueloux, S. van der Werf, T. Prazuck, O. Schwartz, Sensitivity of infectious SARS-CoV-2 B.1.1.7 and B.1.351 variants to neutralizing antibodies. *Nat. Med.* **27**, 917–924 (2021). doi:10.1038/s41591-021-01318-5 Medline
11. P. Wang, R. G. Casner, M. S. Nair, M. Wang, J. Yu, G. Cerutti, L. Liu, P. D. Kwong, Y. Huang, L. Shapiro, D. D. Ho, Increased resistance of SARS-CoV-2 variant P.1 to antibody neutralization. *Cell Host Microbe* **29**, 747–751.e4 (2021). doi:10.1016/j.chom.2021.04.007 Medline
12. P. Wang, M. S. Nair, L. Liu, S. Iketani, Y. Luo, Y. Guo, M. Wang, J. Yu, B. Zhang, P. D. Kwong, B. S. Graham, J. R. Mascola, J. Y. Chang, M. T. Yin, M. Sobieszczyk, C. A. Kyratsous, L. Shapiro, Z. Sheng, Y. Huang, D. D. Ho, Antibody resistance of SARS-CoV-2 variants B.1.351 and B.1.1.7. *Nature* **593**, 130–135 (2021). doi:10.1038/s41586-021-03398-2 Medline
13. W. F. Garcia-Beltran, E. C. Lam, K. St. Denis, A. D. Nitido, Z. H. Garcia, B. M. Hauser, J. Feldman, M. N. Pavlovic, D. J. Gregory, M. C. Poznansky, A. Sigal, A. G. Schmidt, A. J. Iafrate, V. Naranbhai, A. B. Balazs, Multiple SARS-CoV-2 variants escape neutralization by vaccine-induced humoral immunity. *Cell* **184**, 2523 (2021). doi:10.1016/j.cell.2021.04.006 Medline
14. S. A. Clark, L. E. Clark, J. Pan, A. Coscia, L. G. A. McKay, S. Shankar, R. I. Johnson, V. Brusic, M. C. Choudhary, J. Regan, J. Z. Li, A. Griffiths, J. Abraham, SARS-CoV-2 evolution in an immunocompromised host reveals shared neutralization escape mechanisms. *Cell* **184**, 2605–2617.e18 (2021). doi:10.1016/j.cell.2021.03.027 Medline
15. B. Choi, M. C. Choudhary, J. Regan, J. A. Sparks, R. F. Padera, X. Qiu, I. H. Solomon, H.-H. Kuo, J. Boucau, K. Bowman, U. D. Adhikari, M. L. Winkler, A. A. Mueller, T. Y.-T. Hsu, M. Desjardins, L. R. Baden, B. T. Chan, B. D. Walker, M. Lichterfeld, M. Brigl, D. S. Kwon, S. Kanjilal, E. T. Richardson, A. H. Jonsson, G. Alter, A. K. Barczak, W. P. Hanage, X. G. Yu, G. D. Gaiha, M. S. Seaman, M. Cernadas, J. Z. Li, Persistence and

evolution of SARS-CoV-2 in an immunocompromised host. *N. Engl. J. Med.* **383**, 2291–2293 (2020). doi:10.1056/NEJMc2031364 Medline

16. T. N. Starr, A. J. Greaney, A. Addetia, W. W. Hannon, M. C. Choudhary, A. S. Dingens, J. Z. Li, J. D. Bloom, Prospective mapping of viral mutations that escape antibodies used to treat COVID-19. *Science* **371**, 850–854 (2021). doi:10.1126/science.abf9302 Medline

17. A. Baum, B. O. Fulton, E. Wloga, R. Copin, K. E. Pascal, V. Russo, S. Giordano, K. Lanza, N. Negron, M. Ni, Y. Wei, G. S. Atwal, A. J. Murphy, N. Stahl, G. D. Yancopoulos, C. A. Kyratsous, Antibody cocktail to SARS-CoV-2 spike protein prevents rapid mutational escape seen with individual antibodies. *Science* **369**, 1014–1018 (2020). doi:10.1126/science.abd0831 Medline

18. J. Hansen, A. Baum, K. E. Pascal, V. Russo, S. Giordano, E. Wloga, B. O. Fulton, Y. Yan, K. Koon, K. Patel, K. M. Chung, A. Hermann, E. Ullman, J. Cruz, A. Rafique, T. Huang, J. Fairhurst, C. Libertiny, M. Malbec, W. Y. Lee, R. Welsh, G. Farr, S. Pennington, D. Deshpande, J. Cheng, A. Watty, P. Bouffard, R. Babb, N. Levenkova, C. Chen, B. Zhang, A. Romero Hernandez, K. Saotome, Y. Zhou, M. Franklin, S. Sivapalasingam, D. C. Lye, S. Weston, J. Logue, R. Haupt, M. Frieman, G. Chen, W. Olson, A. J. Murphy, N. Stahl, G. D. Yancopoulos, C. A. Kyratsous, Studies in humanized mice and convalescent humans yield a SARS-CoV-2 antibody cocktail. *Science* **369**, 1010–1014 (2020). doi:10.1126/science.abd0827 Medline

19. M. Yuan, H. Liu, N. C. Wu, C. D. Lee, X. Zhu, F. Zhao, D. Huang, W. Yu, Y. Hua, H. Tien, T. F. Rogers, E. Landais, D. Sok, J. G. Jardine, D. R. Burton, I. A. Wilson, Structural basis of a shared antibody response to SARS-CoV-2. *Science* **369**, 1119–1123 (2020). doi:10.1126/science.abd2321 Medline

20. T. F. Rogers, F. Zhao, D. Huang, N. Beutler, A. Burns, W. T. He, O. Limbo, C. Smith, G. Song, J. Woehl, L. Yang, R. K. Abbott, S. Callaghan, E. Garcia, J. Hurtado, M. Parren, L. Peng, S. Ramirez, J. Ricketts, M. J. Ricciardi, S. A. Rawlings, N. C. Wu, M. Yuan, D. M. Smith, D. Nemazee, J. R. Teijaro, J. E. Voss, I. A. Wilson, R. Andrabi, B. Briney, E. Landais, D. Sok, J. G. Jardine, D. R. Burton, Isolation of potent SARS-CoV-2 neutralizing antibodies and protection from disease in a small animal model. *Science* **369**, 956–963 (2020). doi:10.1126/science.abc7520 Medline

21. R. Shi, C. Shan, X. Duan, Z. Chen, P. Liu, J. Song, T. Song, X. Bi, C. Han, L. Wu, G. Gao, X. Hu, Y. Zhang, Z. Tong, W. Huang, W. J. Liu, G. Wu, B. Zhang, L. Wang, J. Qi, H. Feng, F.-S. Wang, Q. Wang, G. F. Gao, Z. Yuan, J. Yan, A human neutralizing antibody targets the receptor-binding site of SARS-CoV-2. *Nature* **584**, 120–124 (2020). doi:10.1038/s41586-020-2381-y Medline

22. E. Seydoux, L. J. Homad, A. J. MacCamy, K. R. Parks, N. K. Hurlburt, M. F. Jennewein, N. R. Akins, A. B. Stuart, Y.-H. Wan, J. Feng, R. E. Whaley, S. Singh, M. Boeckh, K. W. Cohen, M. J. McElrath, J. A. Englund, H. Y. Chu, M. Pancera, A. T. McGuire, L. Stamatatos, Analysis of a SARS-CoV-2-infected individual reveals development of potent neutralizing antibodies with limited somatic mutation. *Immunity* **53**, 98–105.e5 (2020). doi:10.1016/j.immuni.2020.06.001 Medline

23. D. F. Robbiani, C. Gaebler, F. Muecksch, J. C. C. Lorenzi, Z. Wang, A. Cho, M. Agudelo, C. O. Barnes, A. Gazumyan, S. Finkin, T. Hägglöf, T. Y. Oliveira, C. Viant, A. Hurley, H.-

H. Hoffmann, K. G. Millard, R. G. Kost, M. Cipolla, K. Gordon, F. Bianchini, S. T. Chen, V. Ramos, R. Patel, J. Dizon, I. Shimeliovich, P. Mendoza, H. Hartweger, L. Nogueira, M. Pack, J. Horowitz, F. Schmidt, Y. Weisblum, E. Michailidis, A. W. Ashbrook, E. Waltari, J. E. Pak, K. E. Huey-Tubman, N. Koranda, P. R. Hoffman, A. P. West Jr., C. M. Rice, T. Hatziioannou, P. J. Bjorkman, P. D. Bieniasz, M. Caskey, M. C. Nussenzweig, Convergent antibody responses to SARS-CoV-2 in convalescent individuals. *Nature* **584**, 437–442 (2020). doi:10.1038/s41586-020-2456-9 Medline

24. S. Du, Y. Cao, Q. Zhu, P. Yu, F. Qi, G. Wang, X. Du, L. Bao, W. Deng, H. Zhu, J. Liu, J. Nie, Y. Zheng, H. Liang, R. Liu, S. Gong, H. Xu, A. Yisimayi, Q. Lv, B. Wang, R. He, Y. Han, W. Zhao, Y. Bai, Y. Qu, X. Gao, C. Ji, Q. Wang, N. Gao, W. Huang, Y. Wang, X. S. Xie, X. D. Su, J. Xiao, C. Qin, Structurally resolved SARS-CoV-2 antibody shows high efficacy in severely infected hamsters and provides a potent cocktail pairing strategy. *Cell* **183**, 1013–1023.e13 (2020). doi:10.1016/j.cell.2020.09.035 Medline

25. Y. Wu, F. Wang, C. Shen, W. Peng, D. Li, C. Zhao, Z. Li, S. Li, Y. Bi, Y. Yang, Y. Gong, H. Xiao, Z. Fan, S. Tan, G. Wu, W. Tan, X. Lu, C. Fan, Q. Wang, Y. Liu, C. Zhang, J. Qi, G. F. Gao, F. Gao, L. Liu, A noncompeting pair of human neutralizing antibodies block COVID-19 virus binding to its receptor ACE2. *Science* **368**, 1274–1278 (2020). doi:10.1126/science.abc2241 Medline

26. T. N. Starr, A. J. Greaney, A. S. Dingens, J. D. Bloom, Complete map of SARS-CoV-2 RBD mutations that escape the monoclonal antibody LY-CoV555 and its cocktail with LY-CoV016. *Cell Rep. Med.* **2**, 100255 (2021). doi:10.1016/j.xcrm.2021.100255 Medline

27. S. Elbe, G. Buckland-Merrett, Data, disease and diplomacy: GISAID's innovative contribution to global health. *Glob. Chall.* **1**, 33–46 (2017). doi:10.1002/gch2.1018 Medline

28. Y. Weisblum, F. Schmidt, F. Zhang, J. DaSilva, D. Poston, J. C. C. Lorenzi, F. Muecksch, M. Rutkowska, H.-H. Hoffmann, E. Michailidis, C. Gaebler, M. Agudelo, A. Cho, Z. Wang, A. Gazumyan, M. Cipolla, L. Luchsinger, C. D. Hillyer, M. Caskey, D. F. Robbiani, C. M. Rice, M. C. Nussenzweig, T. Hatziioannou, P. D. Bieniasz, Escape from neutralizing antibodies by SARS-CoV-2 spike protein variants. *eLife* **9**, e61312 (2020). doi:10.7554/eLife.61312 Medline

29. D. Wrapp, N. Wang, K. S. Corbett, J. A. Goldsmith, C.-L. Hsieh, O. Abiona, B. S. Graham, J. S. McLellan, Cryo-EM structure of the 2019-nCoV spike in the prefusion conformation. *Science* **367**, 1260–1263 (2020). doi:10.1126/science.abb2507 Medline

30. K. Liu, X. Pan, L. Li, F. Yu, A. Zheng, P. Du, P. Han, Y. Meng, Y. Zhang, L. Wu, Q. Chen, C. Song, Y. Jia, S. Niu, D. Lu, C. Qiao, Z. Chen, D. Ma, X. Ma, S. Tan, X. Zhao, J. Qi, G. F. Gao, Q. Wang, Binding and molecular basis of the bat coronavirus RaTG13 virus to ACE2 in humans and other species. *Cell* **184**, 3438–3451.e10 (2021). doi:10.1016/j.cell.2021.05.031 Medline

31. K. Suryamohan, D. Diwanji, E. W. Stawiski, R. Gupta, S. Miersch, J. Liu, C. Chen, Y.-P. Jiang, F. A. Fellouse, J. F. Sathirapongsasuti, P. K. Albers, T. Deepak, R. Saberianfar, A. Ratan, G. Washburn, M. Mis, D. Santhosh, S. Somasekar, G. H. Hiranjith, D. Vargas, S. Mohan, S. Phalke, B. Kuriakose, A. Antony, M. Ustav Jr., S. C. Schuster, S. Sidhu, J. R. Junutula, N. Jura, S. Seshagiri, Human ACE2 receptor polymorphisms and altered

susceptibility to SARS-CoV-2. *Commun. Biol.* **4**, 475 (2021). doi:10.1038/s42003-021-02030-3 Medline

32. A. R. Mehdipour, G. Hummer, Dual nature of human ACE2 glycosylation in binding to SARS-CoV-2 spike. *Proc. Natl. Acad. Sci. U.S.A.* **118**, e2100425118 (2021). doi:10.1073/pnas.2100425118 Medline

33. X. Zhu, D. Mannar, S. S. Srivastava, A. M. Berezuk, J.-P. Demers, J. W. Saville, K. Leopold, W. Li, D. S. Dimitrov, K. S. Tuttle, S. Zhou, S. Chittori, S. Subramaniam, Cryo-electron microscopy structures of the N501Y SARS-CoV-2 spike protein in complex with ACE2 and 2 potent neutralizing antibodies. *PLOS Biol.* **19**, e3001237 (2021). doi:10.1371/journal.pbio.3001237 Medline

34. R. E. Chen, X. Zhang, J. B. Case, E. S. Winkler, Y. Liu, L. A. VanBlargan, J. Liu, J. M. Errico, X. Xie, N. Suryadevara, P. Gilchuk, S. J. Zost, S. Tahan, L. Droit, J. S. Turner, W. Kim, A. J. Schmitz, M. Thapa, D. Wang, A. C. M. Boon, R. M. Presti, J. A. O'Halloran, A. H. J. Kim, P. Deepak, D. Pinto, D. H. Fremont, J. E. Crowe Jr., D. Corti, H. W. Virgin, A. H. Ellebedy, P.-Y. Shi, M. S. Diamond, Resistance of SARS-CoV-2 variants to neutralization by monoclonal and serum-derived polyclonal antibodies. *Nat. Med.* **27**, 717–726 (2021). doi:10.1038/s41591-021-01294-w Medline

35. A. J. Greaney, A. N. Loes, K. H. D. Crawford, T. N. Starr, K. D. Malone, H. Y. Chu, J. D. Bloom, Comprehensive mapping of mutations in the SARS-CoV-2 receptor-binding domain that affect recognition by polyclonal human plasma antibodies. *Cell Host Microbe* **29**, 463–476.e6 (2021). doi:10.1016/j.chom.2021.02.003 Medline

36. C. O. Barnes, C. A. Jette, M. E. Abernathy, K. A. Dam, S. R. Esswein, H. B. Gristick, A. G. Malyutin, N. G. Sharaf, K. E. Huey-Tubman, Y. E. Lee, D. F. Robbiani, M. C. Nussenzweig, A. P. West Jr., P. J. Bjorkman, SARS-CoV-2 neutralizing antibody structures inform therapeutic strategies. *Nature* **588**, 682–687 (2020). doi:10.1038/s41586-020-2852-1 Medline

37. B. E. Jones, P. L. Brown-Augsburger, K. S. Corbett, K. Westendorf, J. Davies, T. P. Cujec, C. M. Wiethoff, J. L. Blackbourne, B. A. Heinz, D. Foster, R. E. Higgs, D. Balasubramaniam, L. Wang, Y. Zhang, E. S. Yang, R. Bidshahri, L. Kraft, Y. Hwang, S. Žentelis, K. R. Jepson, R. Goya, M. A. Smith, D. W. Collins, S. J. Hinshaw, S. A. Tycho, D. Pellacani, P. Xiang, K. Muthuraman, S. Sobhanifar, M. H. Piper, F. J. Triana, J. Hendle, A. Pustilnik, A. C. Adams, S. J. Berens, R. S. Baric, D. R. Martinez, R. W. Cross, T. W. Geisbert, V. Borisevich, O. Abiona, H. M. Belli, M. de Vries, A. Mohamed, M. Dittmann, M. I. Samanovic, M. J. Mulligan, J. A. Goldsmith, C.-L. Hsieh, N. V. Johnson, D. Wrapp, J. S. McLellan, B. C. Barnhart, B. S. Graham, J. R. Mascola, C. L. Hansen, E. Falconer, The neutralizing antibody, LY-CoV555, protects against SARS-CoV-2 infection in nonhuman primates. *Sci. Transl. Med.* **13**, eabf1906 (2021). doi:10.1126/scitranslmed.abf1906 Medline

38. D. Planas, D. Veyer, A. Baidaliuk, I. Staropoli, F. Guivel-Benhassine, M. M. Rajah, C. Planchais, F. Porrot, N. Robillard, J. Puech, M. Prot, F. Gallais, P. Gantner, A. Velay, J. Le Guen, N. Kassis-Chikhani, D. Edriss, L. Belec, A. Seve, L. Courtellemont, H. Péré, L. Hocqueloux, S. Fafi-Kremer, T. Prazuck, H. Mouquet, T. Bruel, E. Simon-Lorière, F. A.

Rey, O. Schwartz, Reduced sensitivity of SARS-CoV-2 variant Delta to antibody neutralization. *Nature* **596**, 276–280 (2021). doi:10.1038/s41586-021-03777-9 Medline

39. R. E. Chen, E. S. Winkler, J. B. Case, I. D. Aziati, T. L. Bricker, A. Joshi, T. L. Darling, B. Ying, J. M. Errico, S. Shrihari, L. A. VanBlargan, X. Xie, P. Gilchuk, S. J. Zost, L. Droit, Z. Liu, S. Stumpf, D. Wang, S. A. Handley, W. B. Stine Jr., P.-Y. Shi, M. E. Davis-Gardner, M. S. Suthar, M. G. Knight, R. Andino, C. Y. Chiu, A. H. Ellebedy, D. H. Fremont, S. P. J. Whelan, J. E. Crowe Jr., L. Purcell, D. Corti, A. C. M. Boon, M. S. Diamond, In vivo monoclonal antibody efficacy against SARS-CoV-2 variant strains. *Nature* **596**, 103–108 (2021). doi:10.1038/s41586-021-03720-y Medline

40. M. McCallum, J. Bassi, A. De Marco, A. Chen, A. C. Walls, J. Di Iulio, M. A. Tortorici, M.-J. Navarro, C. Silacci-Fregni, C. Saliba, K. R. Sprouse, M. Agostini, D. Pinto, K. Culap, S. Bianchi, S. Jaconi, E. Cameroni, J. E. Bowen, S. W. Tilles, M. S. Pizzuto, S. B. Guastalla, G. Bona, A. F. Pellanda, C. Garzoni, W. C. Van Voorhis, L. E. Rosen, G. Snell, A. Telenti, H. W. Virgin, L. Piccoli, D. Corti, D. Veesler, SARS-CoV-2 immune evasion by the B.1.427/B.1.429 variant of concern. *Science* **373**, 648–654 (2021). doi:10.1126/science.abi7994 Medline

41. E. C. Thomson, L. E. Rosen, J. G. Shepherd, R. Spreafico, A. da Silva Filipe, J. A. Wojcechowskyj, C. Davis, L. Piccoli, D. J. Pascall, J. Dillen, S. Lytras, N. Czudnochowski, R. Shah, M. Meury, N. Jesudason, A. De Marco, K. Li, J. Bassi, A. O'Toole, D. Pinto, R. M. Colquhoun, K. Culap, B. Jackson, F. Zatta, A. Rambaut, S. Jaconi, V. B. Sreenu, J. Nix, I. Zhang, R. F. Jarrett, W. G. Glass, M. Beltramello, K. Nomikou, M. Pizzuto, L. Tong, E. Cameroni, T. I. Croll, N. Johnson, J. Di Iulio, A. Wickenhagen, A. Ceschi, A. M. Harbison, D. Mair, P. Ferrari, K. Smollett, F. Sallusto, S. Carmichael, C. Garzoni, J. Nichols, M. Galli, J. Hughes, A. Riva, A. Ho, M. Schiuma, M. G. Semple, P. J. M. Openshaw, E. Fadda, J. K. Baillie, J. D. Chodera, S. J. Rihn, S. J. Lycett, H. W. Virgin, A. Telenti, D. Corti, D. L. Robertson, G. Snell; ISARIC4C Investigators; COVID-19 Genomics UK (COG-UK) Consortium, Circulating SARS-CoV-2 spike N439K variants maintain fitness while evading antibody-mediated immunity. *Cell* **184**, 1171–1187.e20 (2021). doi:10.1016/j.cell.2021.01.037 Medline

42. L. Wang, T. Zhou, Y. Zhang, E. S. Yang, C. A. Schramm, W. Shi, A. Pegu, O. K. Oloniniyi, A. R. Henry, S. Darko, S. R. Narpala, C. Hatcher, D. R. Martinez, Y. Tsybovsky, E. Phung, O. M. Abiona, A. Antia, E. M. Cale, L. A. Chang, M. Choe, K. S. Corbett, R. L. Davis, A. T. DiPiazza, I. J. Gordon, S. H. Hait, T. Hermanus, P. Kgagudi, F. Laboune, K. Leung, T. Liu, R. D. Mason, A. F. Nazzari, L. Novik, S. O'Connell, S. O'Dell, A. S. Olia, S. D. Schmidt, T. Stephens, C. D. Stringham, C. A. Talana, I.-T. Teng, D. A. Wagner, A. T. Widge, B. Zhang, M. Roederer, J. E. Ledgerwood, T. J. Ruckwardt, M. R. Gaudinski, P. L. Moore, N. A. Doria-Rose, R. S. Baric, B. S. Graham, A. B. McDermott, D. C. Douek, P. D. Kwong, J. R. Mascola, N. J. Sullivan, J. Misasi, Ultrapotent antibodies against diverse and highly transmissible SARS-CoV-2 variants. *Science* **373**, eabh1766 (2021). doi:10.1126/science.abh1766 Medline

43. M. Yuan, D. Huang, C. D. Lee, N. C. Wu, A. M. Jackson, X. Zhu, H. Liu, L. Peng, M. J. van Gils, R. W. Sanders, D. R. Burton, S. M. Reincke, H. Prüss, J. Kreye, D. Nemazee, A. B. Ward, I. A. Wilson, Structural and functional ramifications of antigenic drift in recent

SARS-CoV-2 variants. *Science* **373**, 818–823 (2021). doi:10.1126/science.abh1139 Medline

44. D. Pinto, Y.-J. Park, M. Beltramello, A. C. Walls, M. A. Tortorici, S. Bianchi, S. Jaconi, K. Culap, F. Zatta, A. De Marco, A. Peter, B. Guarino, R. Spreafico, E. Cameroni, J. B. Case, R. E. Chen, C. Havenar-Daughton, G. Snell, A. Telenti, H. W. Virgin, A. Lanzavecchia, M. S. Diamond, K. Fink, D. Veesler, D. Corti, Cross-neutralization of SARS-CoV-2 by a human monoclonal SARS-CoV antibody. *Nature* **583**, 290–295 (2020). doi:10.1038/s41586-020-2349-y Medline

45. C. G. Rappazzo, L. V. Tse, C. I. Kaku, D. Wrapp, M. Sakharkar, D. Huang, L. M. Deveau, T. J. Yockachonis, A. S. Herbert, M. B. Battles, C. M. O'Brien, M. E. Brown, J. C. Geoghegan, J. Belk, L. Peng, L. Yang, Y. Hou, T. D. Scobey, D. R. Burton, D. Nemazee, J. M. Dye, J. E. Voss, B. M. Gunn, J. S. McLellan, R. S. Baric, L. E. Gralinski, L. M. Walker, Broad and potent activity against SARS-like viruses by an engineered human monoclonal antibody. *Science* **371**, 823–829 (2021). doi:10.1126/science.abf4830 Medline

46. C. Liu, H. M. Ginn, W. Dejnirattisai, P. Supasa, B. Wang, A. Tuekprakhon, R. Nutalai, D. Zhou, A. J. Mentzer, Y. Zhao, H. M. E. Duyvesteyn, C. López-Camacho, J. Slon-Campos, T. S. Walter, D. Skelly, S. A. Johnson, T. G. Ritter, C. Mason, S. A. Costa Clemens, F. Gomes Naveca, V. Nascimento, F. Nascimento, C. Fernandes da Costa, P. C. Resende, A. Pauvolid-Correa, M. M. Siqueira, C. Dold, N. Temperton, T. Dong, A. J. Pollard, J. C. Knight, D. Crook, T. Lambe, E. Clutterbuck, S. Bibi, A. Flaxman, M. Bittaye, S. Belij-Rammerstorfer, S. C. Gilbert, T. Malik, M. W. Carroll, P. Klenerman, E. Barnes, S. J. Dunachie, V. Baillie, N. Serafin, Z. Ditse, K. Da Silva, N. G. Paterson, M. A. Williams, D. R. Hall, S. Madhi, M. C. Nunes, P. Goulder, E. E. Fry, J. Mongkolsapaya, J. Ren, D. I. Stuart, G. R. Screaton, Reduced neutralization of SARS-CoV-2 B.1.617 by vaccine and convalescent serum. *Cell* **184**, 4220–4236.e13 (2021). doi:10.1016/j.cell.2021.06.020 Medline

47. L. R. Baden, H. M. El Sahly, B. Essink, K. Kotloff, S. Frey, R. Novak, D. Diemert, S. A. Spector, N. Rouphael, C. B. Creech, J. McGettigan, S. Khetan, N. Segall, J. Solis, A. Brosz, C. Fierro, H. Schwartz, K. Neuzil, L. Corey, P. Gilbert, H. Janes, D. Follmann, M. Marovich, J. Mascola, L. Polakowski, J. Ledgerwood, B. S. Graham, H. Bennett, R. Pajon, C. Knightly, B. Leav, W. Deng, H. Zhou, S. Han, M. Ivarsson, J. Miller, T. Zaks; COVE Study Group, Efficacy and safety of the mRNA-1273 SARS-CoV-2 vaccine. *N. Engl. J. Med.* **384**, 403–416 (2021). doi:10.1056/NEJMoa2035389 Medline

48. F. P. Polack, S. J. Thomas, N. Kitchin, J. Absalon, A. Gurtman, S. Lockhart, J. L. Perez, G. Pérez Marc, E. D. Moreira, C. Zerbini, R. Bailey, K. A. Swanson, S. Roychoudhury, K. Koury, P. Li, W. V. Kalina, D. Cooper, R. W. Frenck Jr., L. L. Hammitt, Ö. Türeci, H. Nell, A. Schaefer, S. Ünal, D. B. Tresnan, S. Mather, P. R. Dormitzer, U. Şahin, K. U. Jansen, W. C. Gruber; C4591001 Clinical Trial Group, Safety and efficacy of the BNT162b2 mRNA covid-19 vaccine. *N. Engl. J. Med.* **383**, 2603–2615 (2020). doi:10.1056/NEJMoa2034577 Medline

49. L. A. Jackson, E. J. Anderson, N. G. Rouphael, P. C. Roberts, M. Makhene, R. N. Coler, M. P. McCullough, J. D. Chappell, M. R. Denison, L. J. Stevens, A. J. Pruijssers, A. McDermott, B. Flach, N. A. Doria-Rose, K. S. Corbett, K. M. Morabito, S. O'Dell, S. D.

Schmidt, P. A. Swanson 2nd, M. Padilla, J. R. Mascola, K. M. Neuzil, H. Bennett, W. Sun, E. Peters, M. Makowski, J. Albert, K. Cross, W. Buchanan, R. Pikaart-Tautges, J. E. Ledgerwood, B. S. Graham, J. H. Beigel; mRNA-1273 Study Group, An mRNA vaccine against SARS-CoV-2 — Preliminary report. *N. Engl. J. Med.* **383**, 1920–1931 (2020). doi:10.1056/NEJMoa2022483 Medline

50. T. G. Ksiazek, D. Erdman, C. S. Goldsmith, S. R. Zaki, T. Peret, S. Emery, S. Tong, C. Urbani, J. A. Comer, W. Lim, P. E. Rollin, S. F. Dowell, A.-E. Ling, C. D. Humphrey, W.-J. Shieh, J. Guarner, C. D. Paddock, P. Rota, B. Fields, J. DeRisi, J.-Y. Yang, N. Cox, J. M. Hughes, J. W. LeDuc, W. J. Bellini, L. J. Anderson; SARS Working Group, A novel coronavirus associated with severe acute respiratory syndrome. *N. Engl. J. Med.* **348**, 1953–1966 (2003). doi:10.1056/NEJMoa030781 Medline

51. Z. Chen, L. Zhang, C. Qin, L. Ba, C. E. Yi, F. Zhang, Q. Wei, T. He, W. Yu, J. Yu, H. Gao, X. Tu, A. Gettie, M. Farzan, K. Y. Yuen, D. D. Ho, Recombinant modified vaccinia virus Ankara expressing the spike glycoprotein of severe acute respiratory syndrome coronavirus induces protective neutralizing antibodies primarily targeting the receptor binding region. *J. Virol.* **79**, 2678–2688 (2005). doi:10.1128/JVI.79.5.2678-2688.2005 Medline

52. Y. Zhu, D. Yu, Y. Han, H. Yan, H. Chong, L. Ren, J. Wang, T. Li, Y. He, Cross-reactive neutralization of SARS-CoV-2 by serum antibodies from recovered SARS patients and immunized animals. *Sci. Adv.* **6**, eabc9999 (2020). doi:10.1126/sciadv.abc9999 Medline

53. H. Lv, N. C. Wu, O. T.-Y. Tsang, M. Yuan, R. A. P. M. Perera, W. S. Leung, R. T. Y. So, J. M. C. Chan, G. K. Yip, T. S. H. Chik, Y. Wang, C. Y. C. Choi, Y. Lin, W. W. Ng, J. Zhao, L. L. M. Poon, J. S. M. Peiris, I. A. Wilson, C. K. P. Mok, Cross-reactive antibody response between SARS-CoV-2 and SARS-CoV infections. *Cell Rep.* **31**, 107725 (2020). doi:10.1016/j.celrep.2020.107725

54. J. Pallesen, N. Wang, K. S. Corbett, D. Wrapp, R. N. Kirchdoerfer, H. L. Turner, C. A. Cottrell, M. M. Becker, L. Wang, W. Shi, W.-P. Kong, E. L. Andres, A. N. Kettenbach, M. R. Denison, J. D. Chappell, B. S. Graham, A. B. Ward, J. S. McLellan, Immunogenicity and structures of a rationally designed prefusion MERS-CoV spike antigen. *Proc. Natl. Acad. Sci. U.S.A.* **114**, E7348–E7357 (2017). doi:10.1073/pnas.1707304114 Medline

55. M. Yuan, N. C. Wu, X. Zhu, C. D. Lee, R. T. Y. So, H. Lv, C. K. P. Mok, I. A. Wilson, A highly conserved cryptic epitope in the receptor binding domains of SARS-CoV-2 and SARS-CoV. *Science* **368**, 630–633 (2020). doi:10.1126/science.abb7269 Medline

56. M. A. Tortorici, N. Czudnochowski, T. N. Starr, R. Marzi, A. C. Walls, F. Zatta, J. E. Bowen, S. Jaconi, J. Di Iulio, Z. Wang, A. De Marco, S. K. Zepeda, D. Pinto, Z. Liu, M. Beltramello, I. Bartha, M. P. Housley, F. A. Lempp, L. E. Rosen, E. Dellota Jr., H. Kaiser, M. Montiel-Ruiz, J. Zhou, A. Addetia, B. Guarino, K. Culap, N. Sprugasci, C. Saliba, E. Vetti, I. Giacchetto-Sasselli, C. S. Fregni, R. Abdelnabi, S. C. Foo, C. Havenar-Daughton, M. A. Schmid, F. Benigni, E. Cameroni, J. Neyts, A. Telenti, H. W. Virgin, S. P. J. Whelan, G. Snell, J. D. Bloom, D. Corti, D. Veesler, M. S. Pizzuto, Broad sarbecovirus neutralization by a human monoclonal antibody. *Nature* **597**, 103–108 (2021). doi:10.1038/s41586-021-03817-4 Medline

57. H. Liu, N. C. Wu, M. Yuan, S. Bangaru, J. L. Torres, T. G. Caniels, J. van Schooten, X. Zhu, C. D. Lee, P. J. M. Brouwer, M. J. van Gils, R. W. Sanders, A. B. Ward, I. A. Wilson, Cross-neutralization of a SARS-CoV-2 antibody to a functionally conserved site is mediated by avidity. *Immunity* **53**, 1272–1280.e5 (2020). doi:10.1016/j.immuni.2020.10.023 Medline

58. Z. Lv, Y.-Q. Deng, Q. Ye, L. Cao, C.-Y. Sun, C. Fan, W. Huang, S. Sun, Y. Sun, L. Zhu, Q. Chen, N. Wang, J. Nie, Z. Cui, D. Zhu, N. Shaw, X.-F. Li, Q. Li, L. Xie, Y. Wang, Z. Rao, C.-F. Qin, X. Wang, Structural basis for neutralization of SARS-CoV-2 and SARS-CoV by a potent therapeutic antibody. *Science* **369**, 1505–1509 (2020). doi:10.1126/science.abc5881 Medline

59. D. Li, R. J. Edwards, K. Manne, D. R. Martinez, A. Schäfer, S. M. Alam, K. Wiehe, X. Lu, R. Parks, L. L. Sutherland, T. H. Oguin 3rd, C. McDanal, L. G. Perez, K. Mansouri, S. M. C. Gobeil, K. Janowska, V. Stalls, M. Kopp, F. Cai, E. Lee, A. Foulger, G. E. Hernandez, A. Sanzone, K. Tilahun, C. Jiang, L. V. Tse, K. W. Bock, M. Minai, B. M. Nagata, K. Cronin, V. Gee-Lai, M. Deyton, M. Barr, T. Von Holle, A. N. Macintyre, E. Stover, J. Feldman, B. M. Hauser, T. M. Caradonna, T. D. Scobey, W. Rountree, Y. Wang, M. A. Moody, D. W. Cain, C. T. DeMarco, T. N. Denny, C. W. Woods, E. W. Petzold, A. G. Schmidt, I.-T. Teng, T. Zhou, P. D. Kwong, J. R. Mascola, B. S. Graham, I. N. Moore, R. Seder, H. Andersen, M. G. Lewis, D. C. Montefiori, G. D. Sempowski, R. S. Baric, P. Acharya, B. F. Haynes, K. O. Saunders, In vitro and in vivo functions of SARS-CoV-2 infection-enhancing and neutralizing antibodies. *Cell* **184**, 4203–4219.e32 (2021). doi:10.1016/j.cell.2021.06.021 Medline

60. T. N. Starr, N. Czudnochowski, Z. Liu, F. Zatta, Y.-J. Park, A. Addetia, D. Pinto, M. Beltramello, P. Hernandez, A. J. Greaney, R. Marzi, W. G. Glass, I. Zhang, A. S. Dingens, J. E. Bowen, M. A. Tortorici, A. C. Walls, J. A. Wojcechowskyj, A. De Marco, L. E. Rosen, J. Zhou, M. Montiel-Ruiz, H. Kaiser, J. R. Dillen, H. Tucker, J. Bassi, C. Silacci-Fregni, M. P. Housley, J. di Iulio, G. Lombardo, M. Agostini, N. Sprugasci, K. Culap, S. Jaconi, M. Meury, E. Dellota Jr., R. Abdelnabi, S. C. Foo, E. Cameroni, S. Stumpf, T. I. Croll, J. C. Nix, C. Havenar-Daughton, L. Piccoli, F. Benigni, J. Neyts, A. Telenti, F. A. Lempp, M. S. Pizzuto, J. D. Chodera, C. M. Hebner, H. W. Virgin, S. P. J. Whelan, D. Veesler, D. Corti, J. D. Bloom, G. Snell, SARS-CoV-2 RBD antibodies that maximize breadth and resistance to escape. *Nature* **597**, 97–102 (2021). doi:10.1038/s41586-021-03807-6 Medline

61. Y. Watanabe, Z. T. Berndsen, J. Raghwani, G. E. Seabright, J. D. Allen, O. G. Pybus, J. S. McLellan, I. A. Wilson, T. A. Bowden, A. B. Ward, M. Crispin, Vulnerabilities in coronavirus glycan shields despite extensive glycosylation. *Nat. Commun.* **11**, 2688 (2020). doi:10.1038/s41467-020-16567-0 Medline

62. O. C. Grant, D. Montgomery, K. Ito, R. J. Woods, Analysis of the SARS-CoV-2 spike protein glycan shield reveals implications for immune recognition. *Sci. Rep.* **10**, 14991 (2020). doi:10.1038/s41598-020-71748-7 Medline

63. M. F. Boni, P. Lemey, X. Jiang, T. T.-Y. Lam, B. W. Perry, T. A. Castoe, A. Rambaut, D. L. Robertson, Evolutionary origins of the SARS-CoV-2 sarbecovirus lineage responsible for the COVID-19 pandemic. *Nat. Microbiol.* **5**, 1408–1417 (2020). doi:10.1038/s41564-020-0771-4 Medline

64. N. K. Hurlburt, E. Seydoux, Y.-H. Wan, V. V. Edara, A. B. Stuart, J. Feng, M. S. Suthar, A. T. McGuire, L. Stamatatos, M. Pancera, Structural basis for potent neutralization of SARS-CoV-2 and role of antibody affinity maturation. *Nat. Commun.* **11**, 5413 (2020). doi:10.1038/s41467-020-19231-9 Medline

65. L. Kang, G. He, A. K. Sharp, X. Wang, A. M. Brown, P. Michalak, J. Weger-Lucarelli, A selective sweep in the Spike gene has driven SARS-CoV-2 human adaptation. *Cell* **184**, 4392–4400.e4 (2021). doi:10.1016/j.cell.2021.07.007 Medline

66. A. M. Harbison, C. A. Fogarty, T. Phung, A. Satheesan, B. L. Schulz, E. Fadda, Fine-tuning the Spike: Role of the nature and topology of the glycan shield in the structure and dynamics of SARS-CoV-2 S. *Chem. Sci.* 10.1039/D1SC04832E (2021). doi:10.1039/D1SC04832E

67. J. D. Bloom, L. I. Gong, D. Baltimore, Permissive secondary mutations enable the evolution of influenza oseltamivir resistance. *Science* **328**, 1272–1275 (2010). doi:10.1126/science.1187816 Medline

68. L. Yurkovetskiy, X. Wang, K. E. Pascal, C. Tomkins-Tinch, T. P. Nyalile, Y. Wang, A. Baum, W. E. Diehl, A. Dauphin, C. Carbone, K. Veinotte, S. B. Egri, S. F. Schaffner, J. E. Lemieux, J. B. Munro, A. Rafique, A. Barve, P. C. Sabeti, C. A. Kyratsous, N. V. Dudkina, K. Shen, J. Luban, Structural and functional analysis of the D614G SARS-CoV-2 spike protein variant. *Cell* **183**, 739–751.e8 (2020). doi:10.1016/j.cell.2020.09.032 Medline

69. W. Kabsch, *XDS*. *Acta Crystallogr. D* **66**, 125–132 (2010). doi:10.1107/S0907444909047337 Medline

70. P. R. Evans, G. N. Murshudov, How good are my data and what is the resolution? *Acta Crystallogr. D* **69**, 1204–1214 (2013). doi:10.1107/S0907444913000061 Medline

71. G. Bricogne, E. Blanc, M. Brandl, C. Flensburg, P. Keller, W. Paciorek, P. Roversi, A. Sharff, O. S. Smart, C. Vonrhein, T. O. Womack, BUSTER version 2.10.3 (Global Phasing Ltd., 2017).

72. P. Emsley, B. Lohkamp, W. G. Scott, K. Cowtan, Features and development of *Coot*. *Acta Crystallogr. D* **66**, 486–501 (2010). doi:10.1107/S0907444910007493 Medline

73. P. D. Adams, P. V. Afonine, G. Bunkóczi, V. B. Chen, I. W. Davis, N. Echols, J. J. Headd, L.-W. Hung, G. J. Kapral, R. W. Grosse-Kunstleve, A. J. McCoy, N. W. Moriarty, R. Oeffner, R. J. Read, D. C. Richardson, J. S. Richardson, T. C. Terwilliger, P. H. Zwart, *PHENIX*: A comprehensive Python-based system for macromolecular structure solution. *Acta Crystallogr. D* **66**, 213–221 (2010). doi:10.1107/S0907444909052925 Medline

74. S. Q. Zheng, E. Palovcak, J.-P. Armache, K. A. Verba, Y. Cheng, D. A. Agard, MotionCor2: Anisotropic correction of beam-induced motion for improved cryo-electron microscopy. *Nat. Methods* **14**, 331–332 (2017). doi:10.1038/nmeth.4193 Medline

75. A. Rohou, N. Grigorieff, CTFFIND4: Fast and accurate defocus estimation from electron micrographs. *J. Struct. Biol.* **192**, 216–221 (2015). doi:10.1016/j.jsb.2015.08.008 Medline

76. T. Wagner, F. Merino, M. Stabrin, T. Moriya, C. Antoni, A. Apelbaum, P. Hagel, O. Sitsel, T. Raisch, D. Prumbaum, D. Quentin, D. Roderer, S. Tacke, B. Siebolds, E. Schubert, T. R. Shaikh, P. Lill, C. Gatsogiannis, S. Raunser, SPHIRE-crYOLO is a fast and accurate fully automated particle picker for cryo-EM. *Commun. Biol.* **2**, 218 (2019). doi:10.1038/s42003-019-0437-z Medline

77. A. Punjani, J. L. Rubinstein, D. J. Fleet, M. A. Brubaker, cryoSPARC: Algorithms for rapid unsupervised cryo-EM structure determination. *Nat. Methods* **14**, 290–296 (2017). doi:10.1038/nmeth.4169 Medline

78. A. C. Walls, Y.-J. Park, M. A. Tortorici, A. Wall, A. T. McGuire, D. Veesler, Structure, function, and antigenicity of the SARS-CoV-2 spike glycoprotein. *Cell* **181**, 281–292.e6 (2020). doi:10.1016/j.cell.2020.02.058 Medline

79. S. Zhang, S. Qiao, J. Yu, J. Zeng, S. Shan, L. Tian, J. Lan, L. Zhang, X. Wang, Bat and pangolin coronavirus spike glycoprotein structures provide insights into SARS-CoV-2 evolution. *Nat. Commun.* **12**, 1607 (2021). doi:10.1038/s41467-021-21767-3 Medline

80. T. Xiao, J. Lu, J. Zhang, R. I. Johnson, L. G. A. McKay, N. Storm, C. L. Lavine, H. Peng, Y. Cai, S. Rits-Volloch, S. Lu, B. D. Quinlan, M. Farzan, M. S. Seaman, A. Griffiths, B. Chen, A trimeric human angiotensin-converting enzyme 2 as an anti-SARS-CoV-2 agent. *Nat. Struct. Mol. Biol.* **28**, 202–209 (2021). doi:10.1038/s41594-020-00549-3 Medline

81. J. Harcourt, A. Tamin, X. Lu, S. Kamili, S. K. Sakthivel, J. Murray, K. Queen, Y. Tao, C. R. Paden, J. Zhang, Y. Li, A. Uehara, H. Wang, C. Goldsmith, H. A. Bullock, L. Wang, B. Whitaker, B. Lynch, R. Gautam, C. Schindewolf, K. G. Lokugamage, D. Scharton, J. A. Plante, D. Mirchandani, S. G. Widen, K. Narayanan, S. Makino, T. G. Ksiazek, K. S. Plante, S. C. Weaver, S. Lindstrom, S. Tong, V. D. Menachery, N. J. Thornburg, Severe acute respiratory syndrome coronavirus 2 from patient with coronavirus disease, United States. *Emerg. Infect. Dis.* **26**, 1266–1273 (2020). doi:10.3201/eid2606.200516 Medline

82. A. R. Aricescu, W. Lu, E. Y. Jones, A time- and cost-efficient system for high-level protein production in mammalian cells. *Acta Crystallogr. D* **62**, 1243–1250 (2006). doi:10.1107/S0907444906029799 Medline

83. C. L. Hsieh, J. A. Goldsmith, J. M. Schaub, A. M. DiVenere, H.-C. Kuo, K. Javanmardi, K. C. Le, D. Wrapp, A. G. Lee, Y. Liu, C.-W. Chou, P. O. Byrne, C. K. Hjorth, N. V. Johnson, J. Ludes-Meyers, A. W. Nguyen, J. Park, N. Wang, D. Amengor, J. J. Lavinder, G. C. Ippolito, J. A. Maynard, I. J. Finkelstein, J. S. McLellan, Structure-based design of prefusion-stabilized SARS-CoV-2 spikes. *Science* **369**, 1501–1505 (2020). doi:10.1126/science.abd0826 Medline

84. J. Misasi, M. S. A. Gilman, M. Kanekiyo, M. Gui, A. Cagigi, S. Mulangu, D. Corti, J. E. Ledgerwood, A. Lanzavecchia, J. Cunningham, J. J. Muyembe-Tamfun, U. Baxa, B. S. Graham, Y. Xiang, N. J. Sullivan, J. S. McLellan, Structural and molecular basis for Ebola virus neutralization by protective human antibodies. *Science* **351**, 1343–1346 (2016). doi:10.1126/science.aad6117 Medline

85. X. Brochet, M. P. Lefranc, V. Giudicelli, IMGT/V-QUEST: The highly customized and integrated system for IG and TR standardized V-J and V-D-J sequence analysis. *Nucleic Acids Res.* **36** (suppl. 2), W503–W508 (2008). doi:10.1093/nar/gkn316 Medline

86. A. J. McCoy, R. W. Grosse-Kunstleve, P. D. Adams, M. D. Winn, L. C. Storoni, R. J. Read, Phaser crystallographic software. *J. Appl. Crystallogr.* **40**, 658–674 (2007). doi:10.1107/S0021889807021206 Medline

87. T. A. Jones, J. Y. Zou, S. W. Cowan, M. Kjeldgaard, Improved methods for building protein models in electron density maps and the location of errors in these models. *Acta Crystallogr. A* **47**, 110–119 (1991). doi:10.1107/S0108767390010224 Medline

88. C. J. Williams, J. J. Headd, N. W. Moriarty, M. G. Prisant, L. L. Videau, L. N. Deis, V. Verma, D. A. Keedy, B. J. Hintze, V. B. Chen, S. Jain, S. M. Lewis, W. B. Arendall 3rd, J. Snoeyink, P. D. Adams, S. C. Lovell, J. S. Richardson, D. C. Richardson, MolProbity: More and better reference data for improved all-atom structure validation. *Protein Sci.* **27**, 293–315 (2018). doi:10.1002/pro.3330 Medline

89. D. D. Pless, W. J. Lennarz, Enzymatic conversion of proteins to glycoproteins. *Proc. Natl. Acad. Sci. U.S.A.* **74**, 134–138 (1977). doi:10.1073/pnas.74.1.134 Medline

90. Y. Watanabe, J. D. Allen, D. Wrapp, J. S. McLellan, M. Crispin, Site-specific glycan analysis of the SARS-CoV-2 spike. *Science* **369**, 330–333 (2020). doi:10.1126/science.abb9983 Medline

91. J. Zivanov, T. Nakane, B. O. Forsberg, D. Kimanius, W. J. H. Hagen, E. Lindahl, S. H. W. Scheres, New tools for automated high-resolution cryo-EM structure determination in RELION-3. *eLife* **7**, e42166 (2018). doi:10.7554/eLife.42166 Medline

92. T. Zhou, Y. Tsybovsky, J. Gorman, M. Rapp, G. Cerutti, G.-Y. Chuang, P. S. Katsamba, J. M. Sampson, A. Schön, J. Bimela, J. C. Boyington, A. Nazzari, A. S. Olia, W. Shi, M. Sastry, T. Stephens, J. Stuckey, I.-T. Teng, P. Wang, S. Wang, B. Zhang, R. A. Friesner, D. D. Ho, J. R. Mascola, L. Shapiro, P. D. Kwong, Cryo-EM structures of SARS-CoV-2 spike without and with ACE2 reveal a pH-dependent switch to mediate endosomal positioning of receptor-binding domains. *Cell Host Microbe* **28**, 867–879.e5 (2020). doi:10.1016/j.chom.2020.11.004 Medline

93. S. Mitternacht, FreeSASA: An open source C library for solvent accessible surface area calculations. *F1000Res.* **5**, 189 (2016). doi:10.12688/f1000research.7931.1 Medline

94. B. Lee, F. M. Richards, The interpretation of protein structures: Estimation of static accessibility. *J. Mol. Biol.* **55**, 379–400 (1971). doi:10.1016/0022-2836(71)90324-X Medline

95. J. Novotný, C. Auffray, A program for prediction of protein secondary structure from nucleotide sequence data: Application to histocompatibility antigens. *Nucleic Acids Res.* **12**, 243–255 (1984). doi:10.1093/nar/12.1Part1.243 Medline

96. A. Pavlova, Z. Zhang, A. Acharya, D. L. Lynch, Y. T. Pang, Z. Mou, J. M. Parks, C. Chipot, J. C. Gumbart, Machine learning reveals the critical interactions for SARS-CoV-2 spike protein binding to ACE2. *J. Phys. Chem. Lett.* **12**, 5494–5502 (2021). doi:10.1021/acs.jpclett.1c01494 Medline

97. F. Li, W. Li, M. Farzan, S. C. Harrison, Structure of SARS coronavirus spike receptor-binding domain complexed with receptor. *Science* **309**, 1864–1868 (2005). doi:10.1126/science.1116480 Medline

98. M. Mirdita, S. Ovchinnikov, M. Steinegger, ColabFold - Making protein folding accessible to all. bioRxiv 2021.2008.2015.456425 [Preprint] (2021). https://doi.org/10.1101/2021.08.15.456425.

99. M. A. Tortorici, M. Beltramello, F. A. Lempp, D. Pinto, H. V. Dang, L. E. Rosen, M. McCallum, J. Bowen, A. Minola, S. Jaconi, F. Zatta, A. De Marco, B. Guarino, S. Bianchi, E. J. Lauron, H. Tucker, J. Zhou, A. Peter, C. Havenar-Daughton, J. A. Wojcechowskyj, J. B. Case, R. E. Chen, H. Kaiser, M. Montiel-Ruiz, M. Meury, N. Czudnochowski, R. Spreafico, J. Dillen, C. Ng, N. Sprugasci, K. Culap, F. Benigni, R. Abdelnabi, S. C. Foo, M. A. Schmid, E. Cameroni, A. Riva, A. Gabrieli, M. Galli, M. S. Pizzuto, J. Neyts, M. S. Diamond, H. W. Virgin, G. Snell, D. Corti, K. Fink, D. Veesler, Ultrapotent human antibodies protect against SARS-CoV-2 challenge via multiple mechanisms. *Science* **370**, 950–957 (2020). doi:10.1126/science.abe3354 Medline

100. Z. Ke, J. Oton, K. Qu, M. Cortese, V. Zila, L. McKeane, T. Nakane, J. Zivanov, C. J. Neufeldt, B. Cerikan, J. M. Lu, J. Peukes, X. Xiong, H.-G. Kräusslich, S. H. W. Scheres, R. Bartenschlager, J. A. G. Briggs, Structures and distributions of SARS-CoV-2 spike proteins on intact virions. *Nature* **588**, 498–502 (2020). doi:10.1038/s41586-020-2665-2 Medline
